# [2,2] Paracyclophanes-based double helicates for constructing artificial light-harvesting systems and white LED device

**DOI:** 10.1038/s41467-023-38405-9

**Published:** 2023-05-12

**Authors:** Zhe Lian, Jing He, Lin Liu, Yanqing Fan, Xuebo Chen, Hua Jiang

**Affiliations:** College of Chemistry, Beijing Normal University, Beijing, 100875 PR China

**Keywords:** Self-assembly, Supramolecular polymers, Organic molecules in materials science

## Abstract

The construction of efficient artificial light-harvesting systems (ALHSs) is of vital importance in utilizing solar energy. Herein, we report the non-covalent syntheses of double helicates **PCP-TPy1/2** and ***R*p**,***R*p-PCP-TPy1/2** by metal-coordination interaction and their applications in ALHSs and white light-emitting diode (LED) device. All double helicates exhibit significant aggregation-induced emission in tetrahydrofuran/water (1:9, v/v) solvent. The aggregated double helicates can be used to construct one-step or sequential ALHSs with fluorescent dyes Eosin Y (EsY) and Nile red (NiR) with the energy transfer efficiency up to 89.3%. Impressively, the PMMA film of **PCP-TPy1** shows white-light emission when doped 0.075% NiR, the solid of double helicates (***R*p**,***R*p-**) **PCP-TPy2** can be used as the additive of a blue LED bulb to achieve white-light emission. In this work, we provided a general method for the preparation of novel double helicates and explored their applications in ALHSs and fluorescent materials, which will promote future construction and application of helicates as emissive devices.

## Abstract (web summary)

The construction of efficient artificial lightharvesting (ALH) systems is of vital importance in utilizing solar energy. Here, the authors report the non-covalent syntheses of double helicates by metalcoordination interaction and their applications in ALHSs and white LED device

## Introduction

During photosynthesis in plants and green bacteria, light energy is captured and efficiently converted into chemical energy for life activities through a large array of chlorophyll^1–3^. Inspired by natural photosynthesis, scientists have devoted intensive efforts to design and construct efficient artificial light-harvesting systems (ALHSs) so as to understand the key aspects of the natural counterparts^4–7^. So far, various artificial objects, including dendrimers^8–11^, porphyrin arrays^12–14^, aggregates^15,16^ and so on^17–21^ have been designed and synthesized. However, multistep synthesis of those ALHSs precluded their scalability and widespread applications. Therefore, developing a novel ALHS and expanding its application are still challenging.

Self-assemblies provide more opportunities to construct ALHSs owing to their easy synthetic access, solution processability, and tunable photophysical properties. Hydrogen bonding-driven self-assembly is an efficient approach to fabricate organic nanoparticles for constructing ALHSs due to their simple synthesis^8,22–24^. On the other hand, self-assemblies driven by coordination bonds^25–28^, including metal-organic frameworks (MOFs)^29–31^, and supramolecular coordination complexes (SCCs)^32–39^, are recognized as excellent platforms for constructing ALHSs and increasingly become one of the most important themes in supramolecular materials during the past three decades. Many attractive SCCs^40–42^ have been successfully developed for guest encapsulation, catalysis, sensing and stabilizing reactive intermediates^43–45^. Compared with other non-covalent interactions, metal-coordination interaction has several advantages. Firstly, metal-coordination interaction can not only precisely control the size and shape of the coordination complexes, but also allow further functionalization of final coordination complex^46–51^. Secondly, it can be still effective at micromolar or even nanomolar concentrations than other weak interactions^52^. Therefore, metal-coordination interaction shows great significance for constructing ALHSs, especially because the study of ALHSs should be performed at the level of micromolar or even nanomolar concentrations. Although SCCs obtained by metal-coordination interaction have good stability and solubility, their applications in efficient ALHSs remain largely unexplored^37^ due to the quenching of emission by transition metals^53^.

In order to solve the quenching of SCCs, a few elegant ALHSs^54–59^ from metallacycles and metallacages have been constructed by taking advantage of the concept of aggregation-induced emission (AIE) proposed by Tang^60,61^. For example, Mukherjee and Stang reported fluorescent hexagonal Pt(II) metallacycles as a novel platform for fabricating ALHSs, which showed aggregation-induced emission enhancement and high energy transfer efficiency^62^. Yang and coworkers reported a supramolecular dual-donor ALHS with an efficient visible light-harvesting capacity from a tetraphenylethylene (TPE) platinum(II) metallacycle^63^. Recently, Zhang and coworkers reported TPE-based tetragonal prismatic platinum(II) metallacages and its applications in ALHSs for photocatalytic cross-coupling hydrogen evolution reaction^64^. Although those SCCs with AIE units exhibited excellent and fascinating properties, there is still a huge demand for developing efficient ALHSs based on novel SCCs.

**Fig. 1:**
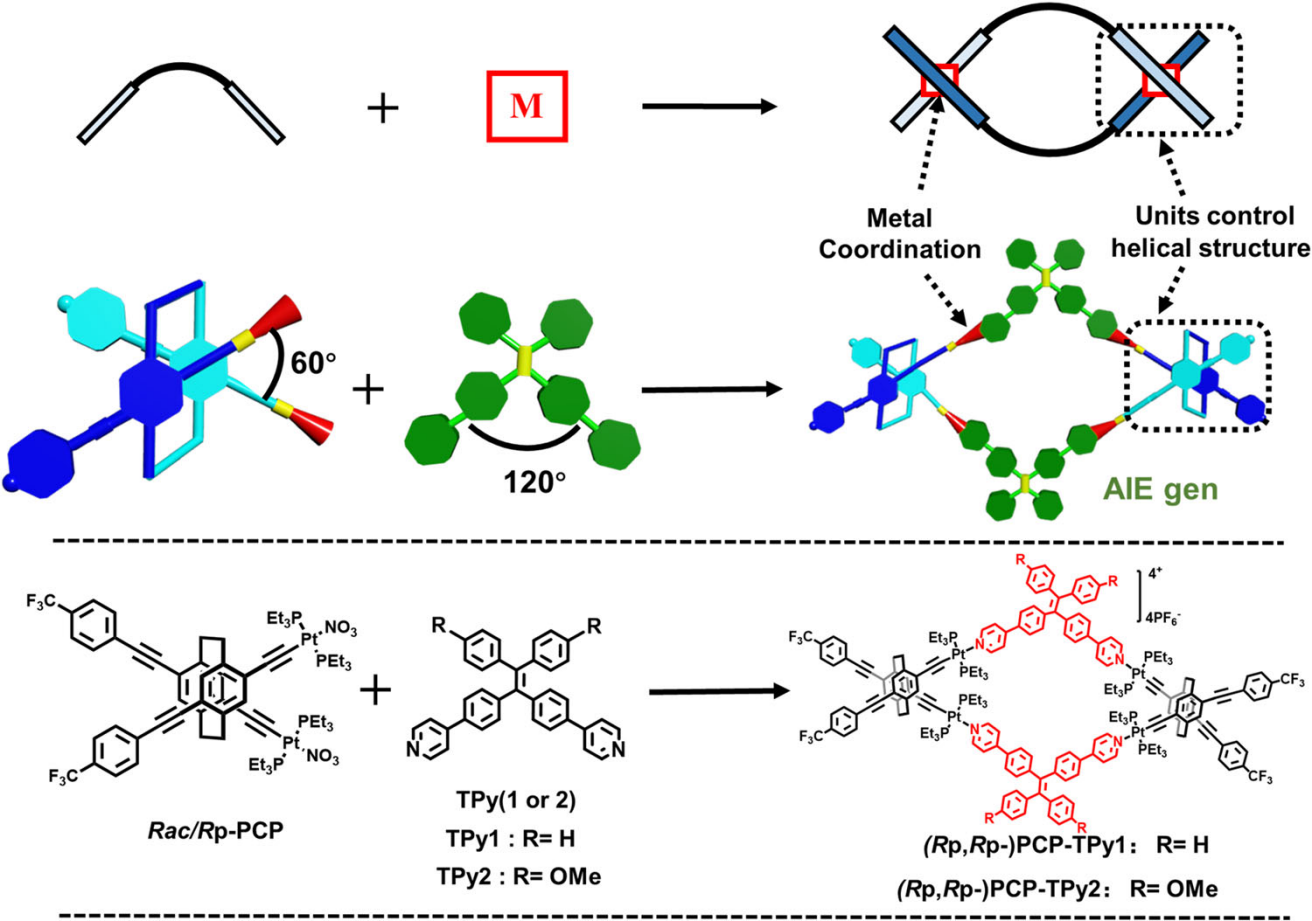
Construction strategy of novel double helicates. Cartoon illustrations of self-assembly of conventional double helicate and novel double helicate in the present work (up) and the non-covalent synthesis of discrete double helicates **PCP-TPy1, PCP-TPy2,** ***R*p,*R*p-PCP-TPy1** and ***R*p,*R*p-PCP-TPy2** from **rac/*R*p-PCP** and AIE units. ((***R*p,*R*p-**) **PCP-TPy1/2** stand for ***R*p,*R*p-PCP-TPy1/2** and **PCP-TPy1/2**, the latter may contain one pair of enantiomers ***R*p,*R*p-PCP-TPy1/2** and ***S*p,*S*p-PCP-TPy1/2** and one mesomer ***R*p,*S*p-PCP-TPy1/2**.).

Double helicate is one of versatile supramolecular complexes resulting from coordination between metals and two organic strands^65–68^. As a consequence of metal coordination, the helical structure of double helicate is mainly governed by the configuration of organic strands at the metal centers. [2.2]Paracyclophane (PCP), which displays diverse planar chiral structures due to its pseudo-*para*, *meta* and *ortho* orientations, has been widely utilized as chiral auxiliaries and ligands^69–71^ for diverse applications from asymmetric synthesis to supramolecular assembly^72^, polymer chemistry^73^ and material science^74^. Recently, Chujo and coworkers constructed one-handed double helices based on planar chiral PCP^75,76^. Encouraged by these significant progresses on PCP, we took advantage of the unique 3-dimensional (3D), planar chiral structure of tetrasubstituted [2.2]PCP as a key building block to design novel double helicates (Fig. 1). Specifically, the *pseudo-ortho-* (*po-*) orientation of 4,12,-bis(ethynyl) [2,2]PCP derivative provides a chance for us to design and synthesize racemic and chiral 60° diplatinum(II) complexes (***rac/Rp-*PCP**) (Fig. 1). When ***rac/Rp-*PCP** interacted with 120° dipyridyl TPE ligands (TPy1/TPy2) featuring AIE property^77–79^ and thus forms double helicates **PCP-Tpy1** and **PCP-Tpy2** and enantiomeric pure double helicates (***R*p,*R*p-PCP-Tpy1** and ***R*p,*R*p-PCP-Tpy2**) with a rhomboidal cavity, respectively, in which the helical structure is dominated by 3D planar chiral [2.2]PCP units rather than Pt-N metal-coordination centers (Fig. 1). Notably, these two double helicates **PCP-Tpy1** and **PCP-Tpy2** can be further assembled into nanoaggregates in THF/water (1:9, v/v) mixed solution and exhibit the excellent characteristic of AIE. By taking advantage of the AIE properties and spherical morphology of double helicates in THF/water (1:9, v/v), one-step and sequential ALHSs were successfully fabricated by adding fluorescent dyes (EsY and NiR) with the excellent energy transfer efficiency up to 89.3%. Moreover, chiral double helicates ***R*p,*R*p-PCP-Tpy1** and ***R*p,*R*p-PCP-Tpy2** also exhibited the similar optical properties to racemic counterparts. Interestingly, **PCP-TPy1** can further be used to construct white-light fluorescent film with NiR, while **PCP-TPy2** can be integrated with a 460 nm LED chip to fabricate white LED devices.

## Results and discussion

### Structural characterization of the double helicates

The synthetic procedures of *rac*/*Rp*− 60° *pseudo-ortho-*bis(ethynyl) [2.2]PCP diplatinum(II) complexes (***rac/Rp*-PCP**) and 120° dipyridyl TPE (**TPy**) were depicted in Supplementary Figs. 1 and 2. Chiral double helicates ***R*p,*R*p-PCP-TPy1** and ***R*p,*R*p-PCP-TPy2** were prepared in nearly quantitative yield by stirring diplatinum(II) complex ***R*p-PCP** with corresponding **TPy1** or **TPy2** in the mixed solvents of dichloromethane and acetone at 60 °C overnight. Similarly, double helicates **PCP-TPy1** and **PCP-TPy2** were also prepared in nearly quantitative yields by stirring diplatinum(II) complex ***rac-*PCP** with corresponding **TPy1** or **TPy2** under identical conditions. These all double helicates were characterized by various analytic techniques, including ^1^H NMR, ^31^P{^1^H} NMR, and 2D diffusion-ordered ^1^H NMR spectroscopy (DOSY) and electrospray ionization time-of-flight mass spectrometry (ESI-TOF-MS).

**Fig. 2:**
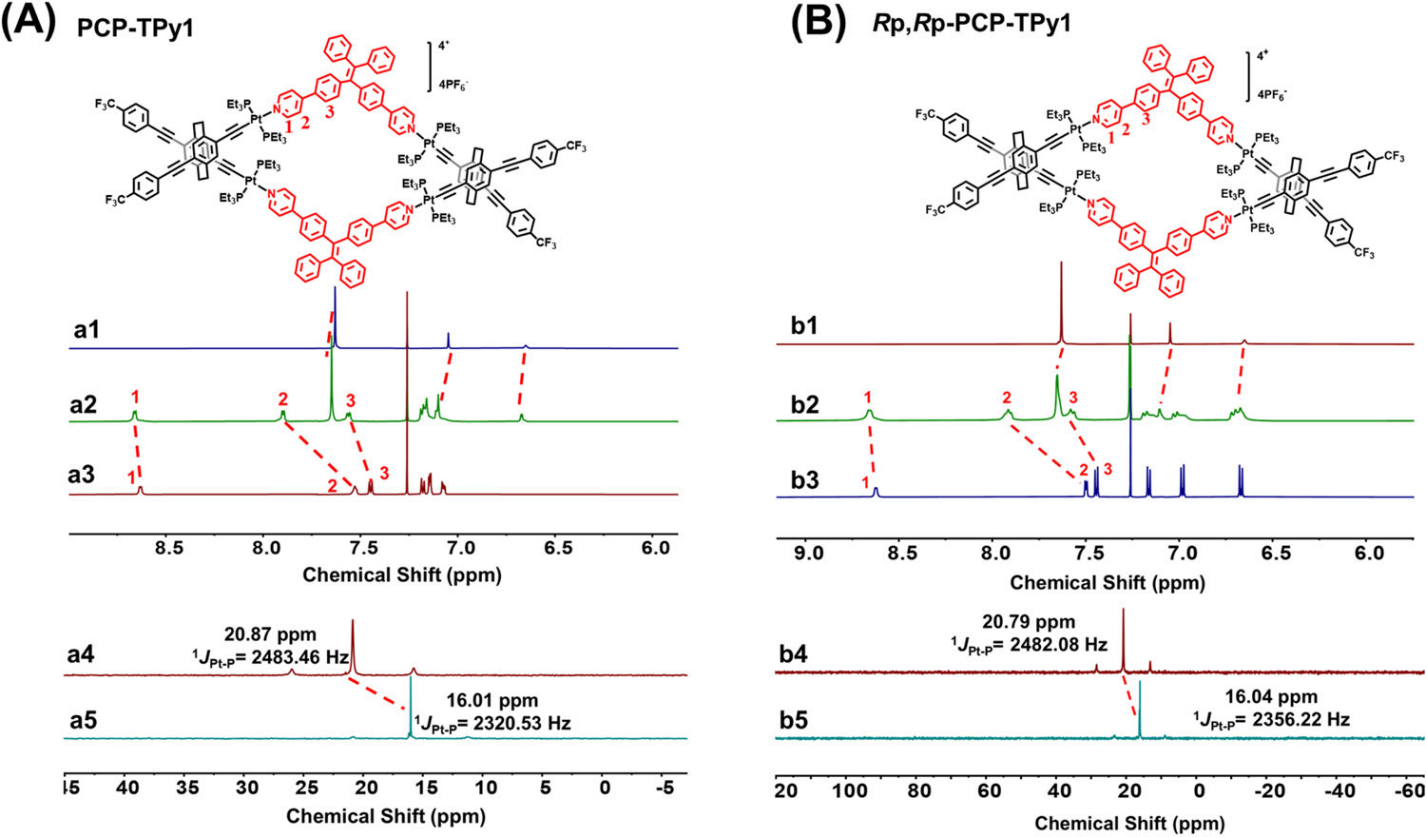
NMR spectra. **A** Partial ^1^H NMR spectra (600 M, CDCl_3_, 298 K) of double helicate **PCP-TPy1**. (a1) ***rac*-PCP**, (a2) **PCP-TPy1**, (a3) **TPy1**. ^31^P NMR spectra (242 M, CDCl_3_, 298 K) of double helicate **PCP-TPy1**. (a4) ***rac*-PCP**, (a5) **PCP-TPy1**. **B** Partial ^1^H NMR spectra (400 M, CDCl_3_, 298 K) of double helicate ***R*p,*R*p-PCP-TPy1**. (b1) ***R*p-PCP**, (b2) ***R*p,*R*p-PCP-TPy1**, (b3) **TPy1**. ^31^P NMR spectra (162 M, CDCl3, 298 K) of ***R*p*.R*p-PCP-TPy1**. (b4) ***R*p-PCP**, (b5) ***R*p*,R*p-PCP-TPy1**.

^1^H NMR spectrum of **PCP-TPy1** clearly shows a set of peaks (Fig. 2A). Downfield chemical shifts were observed for α-pyridyl protons H_1_, β-pyridyl protons H_2_ and phenyl protons H_3_ with Δ*δ* = 0.02, 0.37 and 0.12 ppm, respectively, owing to the coordination of the pyridyl moieties with the platinum atoms. In the ^31^P{1H} NMR spectra (Fig. 2A), the singlet peak displays an obvious upfield shift from 20.87 to 16.01 ppm for **PCP-TPy1**, which is a characteristic feature for the formation of metal-coordination bonds. The similar changes in the ^1^H NMR and ^31^P{1H} NMR spectra were also observed for ***R*p,*R*p-PCP-TPy1, PCP-TPy2** and ***R*p*,R*p-PCP-TPy2** (Fig. 2B and Supplementary Figs 3 and 4).

The mass of **PCP-TPy1** and **PCP-TPy2** were determined by ESI-TOF-MS analysis. The mass peaks at *m/z* 2084.61, 1341.42 and 969.82 were found for **PCP-TPy1**, corresponding to the charge states of [M-2PF_6_^−^]^2+^, [M-3PF_6_^−^]^3+^ and [M-4PF_6_^−^]^4+^, respectively (Supplementary Fig. 5). Similarly, the mass peaks at *m/z* 2144.64, 1381.76 and 999.83 were observed for **PCP-TPy2**, corresponding to the charge states of [M-2PF_6_^−^]^2+^, [M-3PF_6_^−^]^3+^ and [M-4PF_6_^−^]^4+^, respectively (Supplementary Fig. 6). The similar mass peaks were also found for chiral ***R*p*,R*p-PCP-TPy1** and ***R*p*,R*p-PCP-TPy2** (Supplementary Figs. 7 and 8). Moreover, ^1^H DOSY experiment revealed that all the proton signals possessed the same diffusion coefficient (*D*) with *D* = 6.3 × 10^−10^ m^2^ s^−1^ for **PCP-TPy1** and *D* = 3.6 × 10^−10^ m^2^ s^−1^ for **PCP-TPy2** in CDCl_3_, respectively, suggesting the formation of single discrete structure (Supplementary Figs. 9 and 10). The diffusion coefficients of 7.9 × 10^−10^ m^2^ s^−1^ and 3.9 × 10^−10^ m^2^ s^−1^ were also determined for ***R*p*,R*p-PCP-TPy1** and ***R*p*,R*p-PCP-TPy2**, respectively, (Supplementary Figs. 11 and 12). The diffusion coefficients of **PCP-TPy1/2** and their enantiomeric pure counterparts are comparable, which tends to indicate that their sizes are very similar.

The above characterizations clearly show the formations of double helicates, however, one may expected that double helicates **PCP-TPy1/2** contained one pair of enantiomers (***S*p,*S*p-PCP-TPy1/2** and ***R*p,*R*p-PCP-TPy1/2**) and one mesomer (***meso*-PCP-TPy1/2**). Therefore, we optimized the double helical structures of ***R*p,*R*p-PCP-TPy1** and ***meso*-PCP-TPy1** by the density functional theory (DFT) method at the B3LYP-D3/6-31G(d) level (Supplementary Figs. 13 and 14). The computational results indicate that the Gibbs free energies (G) of ***meso*-PCP-TPy1** and ***R*p,*R*p-PCP-TPy1** are almost same (Supplementary Table 1), thus implying that social and narcissistic chiral self-sorting may occur during the formations of double helicates **PCP-TPy1/2** even though all their characterization data are the same as those of their chiral counterparts. However, as demonstrated in following investigations, we found that double helicates **PCP-TPy1/2** exhibited the excellent properties including AIE and energy transfer similar to those of the chiral ones, thus suggesting that the presence of *meso*-double helicates did not display the detectable effect on the properties of double helicates.

### AIE properties and assembly of double helicates

Next, the photophysical properties of these double helicates in pure THF were investigated by UV/Vis absorption and fluorescence spectroscopies. **PCP-TPy1** and **PCP-TPy2** were found to be completely soluble in pure THF and showed pronounced absorption peaks at 360 nm with the molar absorption coefficients (*ε*) of 1.50 × 10^5^ and at 375 nm with *ε* of 1.80 × 10^5^ M^−1^ cm^−1^, respectively (Supplementary Fig. 15). The fluorescence investigations revealed that **PCP-TPy1** showed a main emission peak at 475 nm and a shoulder emission peak at 450 nm in pure THF upon excitation at 360 nm, while **PCP-TPy2** showed a similar emission contour with peaks at 530 and 450 nm under the same conditions. The UV/Vis absorption and fluorescence spectra of chiral double helicates ***R*p*,R*p-PCP-TPy1/2** were similar to those of **PCP-TPy1/2** in pure THF (Supplementary Figs. 16 and 17).

**Fig. 3:**
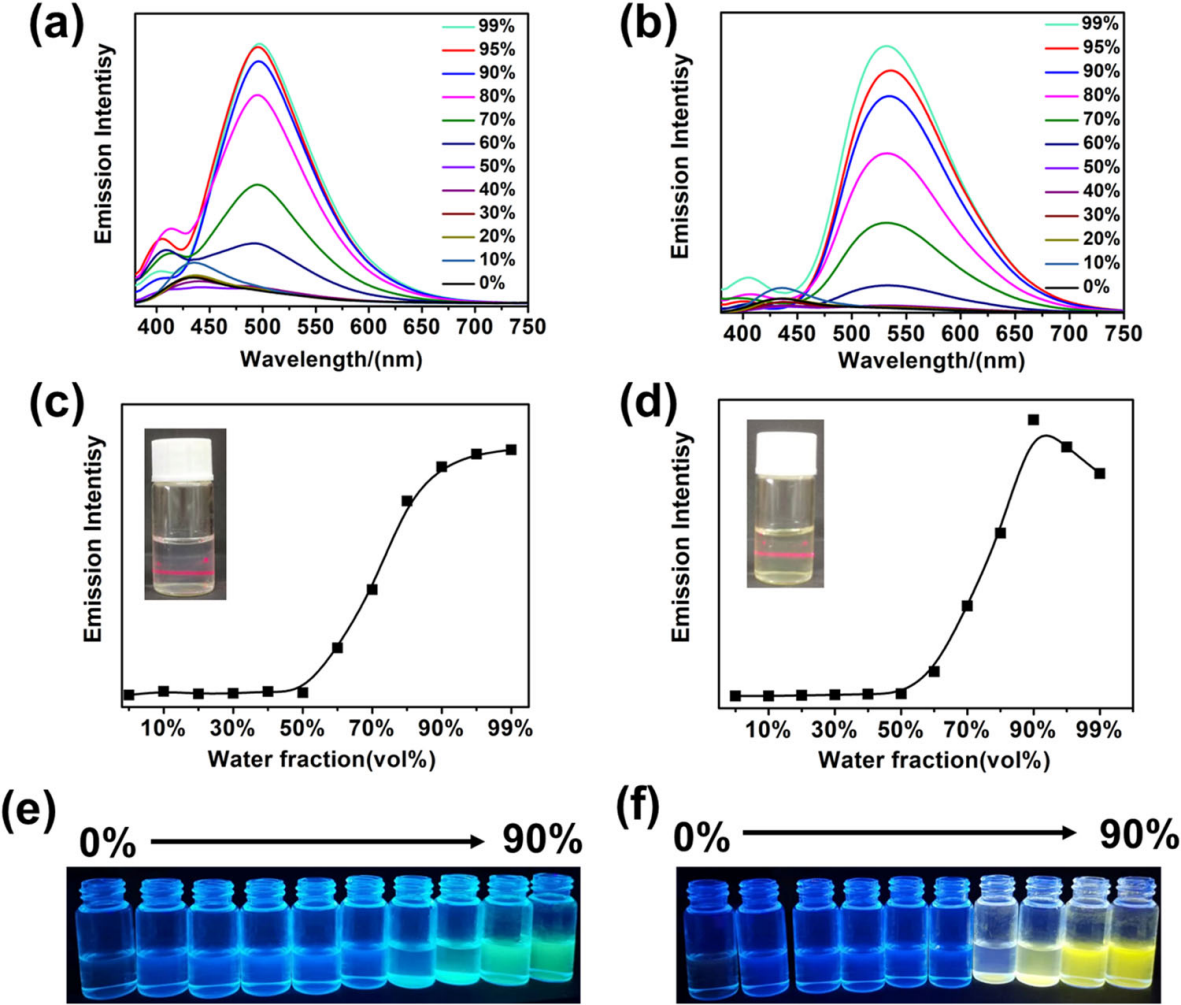
The AIE properties of novel double helicates. Fluorescence emission spectra and the emission intensity curves of **PCP-TPy1** (**a**, **c**) and **PCP-TPy2** (**b**, **d**) with different water fraction in THF solvent (λ_ex_ = 360 nm). The changes in fluorescence color of **PCP-TPy1** (**e**) and **PCP-TPy2** (**f**) with different water fraction in THF solvent under 365 nm UV light. The inserted pictures in c and d are the Tyndall phenomena of **PCP-TPy1** and **PCP-TPy2**, respectively. All concentrations are 1 × 10^−5^ M.

The AIE properties of **PCP-TPy1** and **PCP-TPy2** in THF/water mixtures were studied by UV/Vis absorption and fluorescence spectroscopies. **PCP-TPy1** and **PCP-TPy2** showed a broad absorption band at 360 nm and 375 nm in the mixture of THF/water solvent, respectively. (Supplementary Fig. 18). The emission intensities of double helicates showed slight enhancement with increasing amount of water up to 60% in THF (Fig. 3a, b). However, when the water fractions were over 60%, a new, stronger emission peak at 495 for **PCP-TPy1** or at 530 nm for **PCP-TPy2** was observed. The significant enhancement of fluorescent emission can be accounted for by AIE effect, that is, the free rotations of the phenyl groups in the **TPy** units are strictly restrained due to molecular aggregation, which significantly reduces the non-radiative decay and thus enhances the emissions of the double helicates. It is worth noting that there is an obvious difference in the maximum emission wavelengths between **PCP-TPy1** and **PCP-TPy2** in aggregated states, which is attributed to the presence of the methoxyl groups in **PCP-TPy2**. The significant red-shifts in the fluorescence emission peaks from 435 to 495 nm for **PCP-TPy1** or to 530 nm for **PCP-TPy2** are due to the change of emission entities from single molecules to their aggregation states. In addition, **PCP-TPy1** experienced a change of emission color from blue to green with the increase of water fractions (Fig. 3e). However, the emission color of **PCP-TPy2** changed from blue to yellow (Fig. 3f). Furthermore, when the water content reached 80%, the solutions of double helicates exhibited a clear Tyndall effect (Fig. 3c, d), as the clear evidence for the existence of nanoaggregates. We further examined the AIE properties of ***R*p,*R*p-PCP-TPy1** and ***R*p,*R*p-PCP-TPy2** with the same procedures as performed for **PCP-TPy1/2**. The UV/Vis absorption and fluorescence investigations revealed that ***R*p,*R*p-PCP-TPy1/2** exhibit the same optical properties as **PCP-TPy1/2** under the same conditions (Supplementary Figs. 16–22).

The measurements of quantum yields (*ϕ*_F_) (Supplementary Fig. 24) in different water content further support the AIE effects of double helicates. The *ϕ*_F_ values of **PCP-TPy1** and **PCP-Tpy2** in pure THF were measured to be 0.7% and 0.5%, respectively. The *ϕ*_F_ values increased to 1.3% and 2.8%, when the water fraction got to 60%. At the water fraction increased to 90%, the *ϕ*_F_ values of **PCP-TPy1** and **PCP-TPy2** increased to 4.9% and 5.0%, respectively. Furthermore, the *ϕ*_F_ values of **PCP-TPy1** and **PCP-Tpy2** in the mixture of CH_2_Cl_2_/PE were measured to be 5.2% and 13.2%, respectively. (Supplementary Figs. 25 and 26, Supplementary Table 2)

**Fig. 4:**
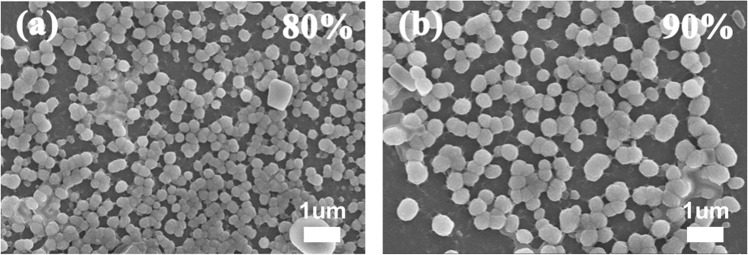
The SEM images of PCP-TPy1 in the mixture of THF/water (**a** 2:8, v/v. **b** 1:9, v/v).

The size and shape of these nanoaggregates of **PCP-TPy1** and **PCP-TPy2** in THF/water (1:9, v/v) were further studied by scanning electron microscopy (SEM) (Fig. 4a, b and Supplementary Fig. 27). The SEM investigations demonstrated that **PCP-TPy1** and **PCP-TPy2** formed well-defined spherical nanoaggregates. The hydrodynamic diameters of **PCP-TPy1** nanoaggregates were found to be 20 nm and 130 nm by dynamic light scattering (DLS) measurement (Supplementary Fig. 28). Those of **PCP-Tpy2** nanoaggregates were found to be 32 nm and 277 nm by DLS measurement (Supplementary Fig. 28). Chiral double helicates ***R*p,*R*p-PCP-TPy1/2** also exhibited the similar morphologies to their racemic counterparts as revealed by SEM and DLS (Supplementary Figs. 29–31). These observations combined with the optical properties suggest that the aggregates of double helicates **PCP-TPy1/2** and ***R*p,*R*p-PCP-TPy1/2** in THF/water (1:9, v/v) have very similar optical properties and self-assembly behaviors, which can be used as a new class of excellent platforms for fabricating ALHSs.

### Novel ALHSs based on double helicates

**Fig. 5:**
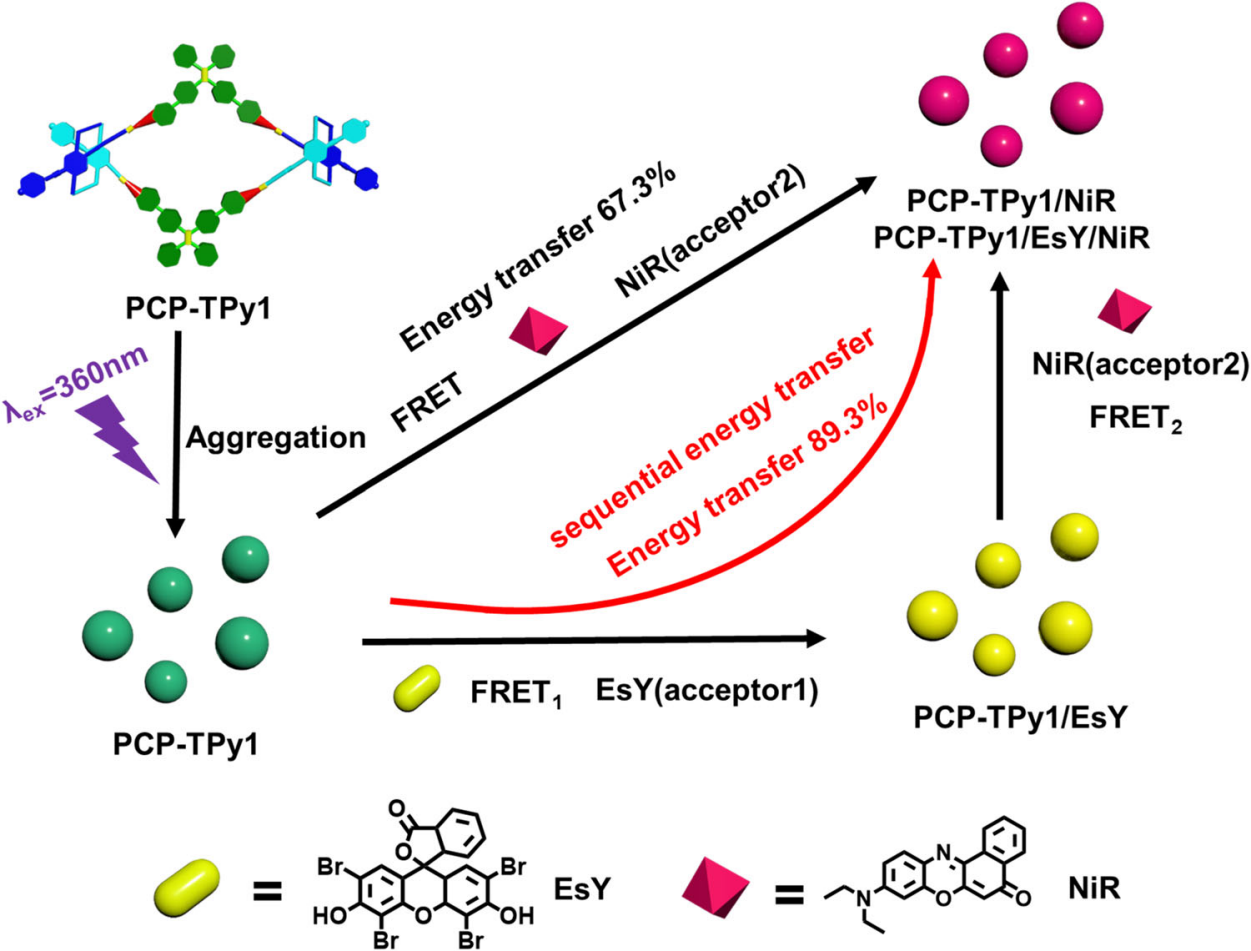
Construction strategy of ALHSs based on double helicate PCP-TPy1.

It is well-known that the absorption band of the energy acceptor should efficiently overlap with the emission band of the energy donor for constructing ALHSs. In the present case, the absorption spectra of energy acceptors Eosin Y (EsY) and Nile red (NiR) overlap well with the emission spectrum of the energy donor **PCP-TPy1** (Supplementary Figs. 32 and 34). Similar overlap was also observed for the absorption band of NiR and the emission band of **PCP-TPy2** (Supplementary Figs. 32 and 37). The light-harvesting behaviors of double helicates and fluorescent dye were investigated in pure THF firstly. Unfortunately, no energy transfer between double helicates and EsY/NiR was detected in pure THF because the dye molecules were not in a well-organized array (Supplementary Fig. 33). Then, the light-harvesting behaviors of double helicates and fluorescent dyes were investigated in THF/water (1:9, v/v) solvent. The construction strategy of ALHSs is presented in Fig. 5.

**Fig. 6:**
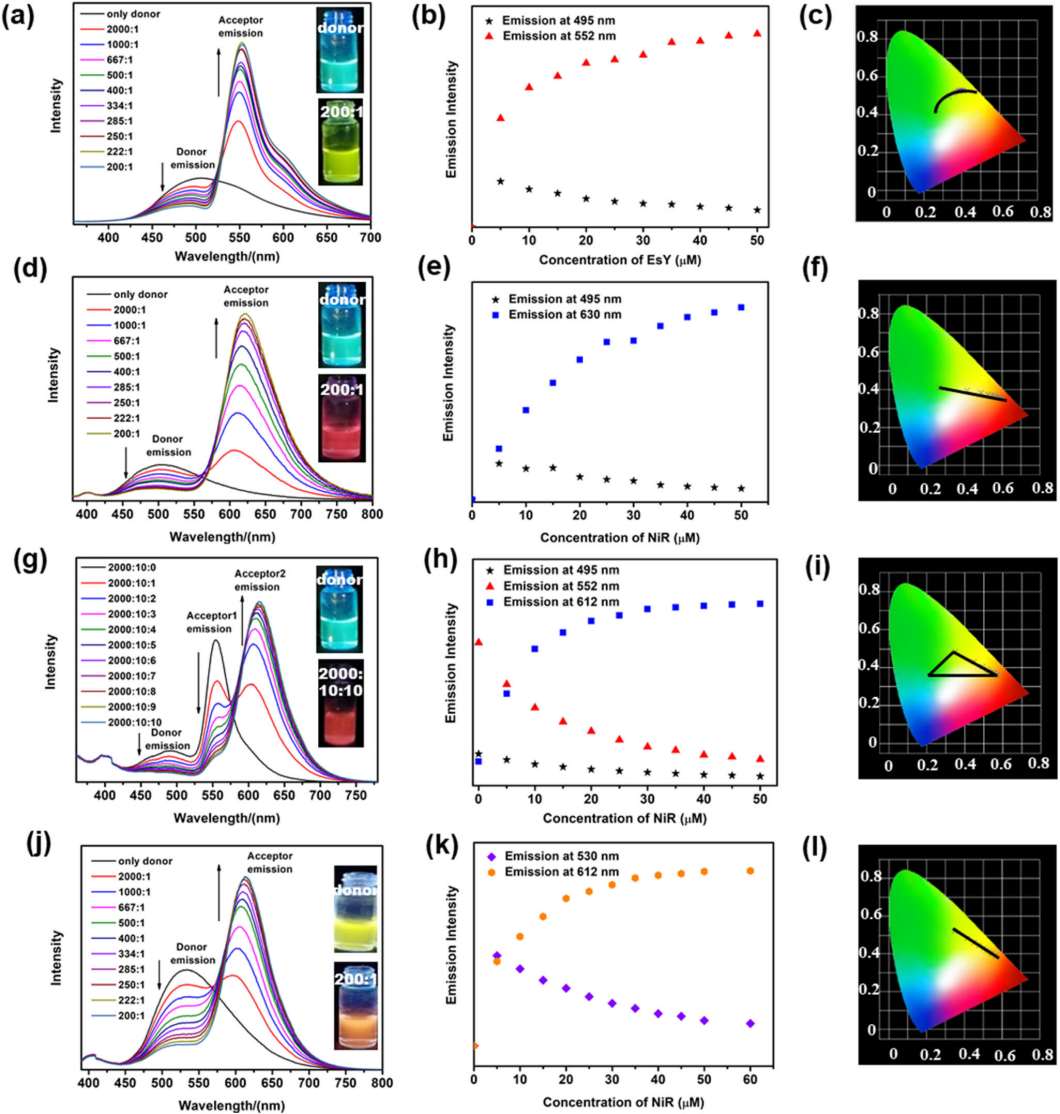
ALHSs based on novel double helicates. Fluorescence emission spectra (**a**) of **PCP-TPy1** (1 × 10^−5^ M) with different concentrations of EsY(1 × 10^−7^ M), Fluorescent intensity changes (**b**) at 495 and 552 nm, Fluorescence emission of **PCP-TPy1/EsY** in the CIE (**c**), Fluorescence emission spectra (**d**) of **PCP-TPy1** (1 × 10^−5^ M) with different concentrations of NiR(1 × 10^−7^ M), Fluorescent intensity changes (**e**) at 495 and 630 nm. Fluorescence emission of **PCP-TPy1/NiR** in the CIE (**f**), Fluorescence emission spectra (**g**) of **PCP-TPy1/EsY** (1 × 10^−5^ M) with different concentrations of NiR (1 × 10^−7^ M), Fluorescent intensity changes (**h**) at 495, 552 and 612 nm, Fluorescence emission of **PCP-TPy1/EsY/NiR** in the CIE (**i**), Fluorescence emission spectra (**j**) of **PCP-TPy2** (1 × 10^−5^ M) with different concentrations of NiR(1 × 10^−7^ M), Fluorescent intensity changes (**k**) at 530 and 612 nm, Fluorescence emission of **PCP-TPy2/NiR** in the CIE (**l**). All experiments were performed in the mixture of THF/water (1:9, v/v), λ_ex_ = 360 nm, slit widths: ex = 5 nm, em = 5 nm.

**Fig. 7:**
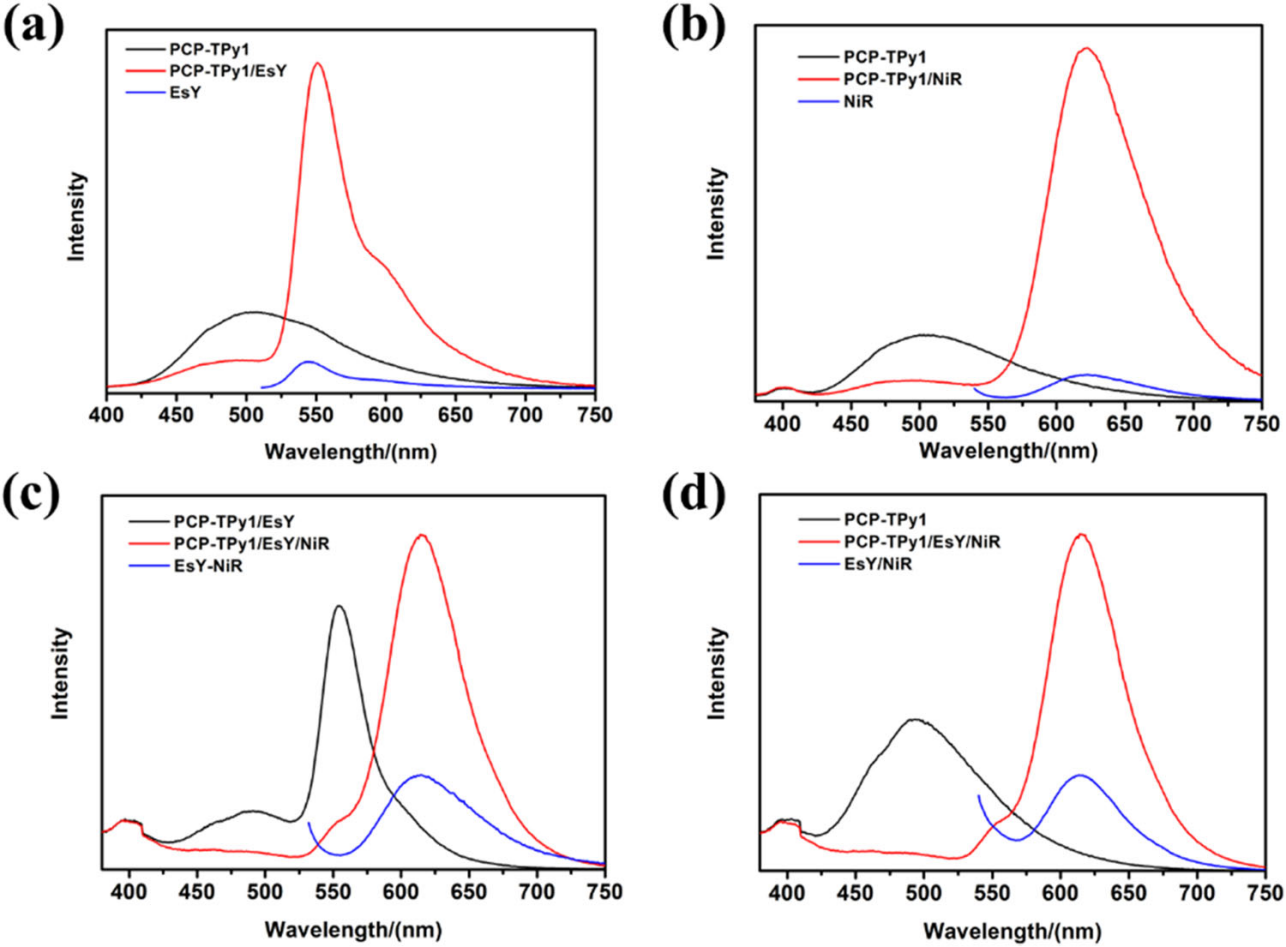
The energy transfer efficiency of ALHSs. (**a**) Fluorescence spectra of **PCP-TPy1/EsY** in THF/water, blue line (acceptor emission, λ_ex_ = 500 nm), the black line represents the fluorescence spectrum of **PCP-TPy1**, which was normalized according to the fluorescence intensity at 552 nm of the red line, (**b**) Fluorescence spectra of **PCP-TPy1/NiR** in THF/water, blue line (acceptor emission, λ_ex_ = 530 nm), the black line represents the fluorescence spectrum of **PCP-TPy1**, which was normalized according to the fluorescence intensity at 630 nm of the red line, (**c**) Fluorescence spectra of **PCP-TPy1/EsY/NiR** in THF/Water, blue line (acceptor emission, λ_ex_ = 530 nm), the black line represents the fluorescence spectrum of **PCP-TPy1/EsY**, which was normalized according to the fluorescence intensity at 630 nm of the red line, (**d**) Fluorescence spectra of **PCP-TPy1/EsY/NiR** in THF/Water, blue line (acceptor emission, λ_ex_ = 530 nm), the black line represents the fluorescence spectrum of **PCP-TPy1**, which was normalized according to the fluorescence intensity at 630 nm of the red line. All experiments were performed in the mixture of THF/water (1:9, v/v), **PCP-TPy1** (1 × 10^−5^ M)**, EsY** (1 × 10^−7^ M), NiR (1 × 10^−7^ M), λ_ex_ = 360 nm, slit widths: ex = 5 nm, em = 5 nm.

The light-harvesting behavior of **PCP-TPy1** and energy acceptor EsY was investigated firstly. As shown in Fig. 6a, as the gradual addition of EsY into the solution (THF/water=1:9, v/v) of **PCP-TPy1**, the emission intensity of the energy donor **PCP-TPy1** at 495 nm receded gradually, that of the energy acceptor EsY at 552 nm increased as the system was excited at 360 nm. When the ratio of the energy donor **PCP-TPy1** and the energy acceptor EsY reached 2000:1, an obvious antenna effect was observed. Moreover, the fluorescence intensity remained almost unchanged when the ratio of the **PCP-TPy1** and EsY reached 200:1 (Fig. 6b). Based on the 1931 Commission Internationale de L’Eclairage (CIE) chromaticity diagram, the emission of this ALHS changed from green region to yellow one (Fig. 6c). These observed phenomena indicated that the energy transfer from **PCP-TPy1** to EsY took place indeed, and a supramolecular ALHS was constructed successfully. To further evaluate these ALHS, the energy transfer efficiency and antenna effect were calculated to be 63.5% and 12.8, respectively, for the system of **PCP-TPy1** and EsY at the ratio of 200:1 excited at 360 nm. (Fig. 7a and Supplementary Table 3).

The ALHS of **PCP-TPy1** and NiR was also constructed. As shown in Fig. 6d, with the addition of NiR into the solution (THF/water=1:9, v/v) of **PCP-TPy1**, the emission of the **PCP-TPy1** (495 nm) decreased and that of the NiR (630 nm) increased concomitantly. This system also experienced a change of emission color from green to pink as the increase of NiR fractions (Fig. 6d). The energy transfer efficiency and antenna effect were calculated to be 67.3% and 12.2, respectively, for the system of **PCP-TPy1** and NiR at the ratio of 200:1 when excited at 360 nm (Fig. 7b and Supplementary Table 3). To further improve the energy transfer efficiency, an ALHS with sequential energy transfer (SET) constructed from **PCP-TPy1**, EsY and NiR was investigated further. With the addition of NiR into the ALHS **PCP-TPy1/EsY**, the emission band at 612 nm assignable to NiR increased gradually, while the emission peaks of **PCP-TPy1** at 495 nm and EsY at 552 nm decreased concurrently, accompanying by the visual fluorescent color changes from green to pink (Fig. 6g). These phenomena showed that the energy transfer from **PCP-TPy1** to EsY and finally to NiR took place, a sequential energy transfer system has been constructed successfully. Based on the 1931 Commission Internationale de L’Eclairage (CIE) chromaticity diagram, the emission of this ALHS changed from green to yellow and red region (Fig. 6i). In the **PCP-TPy1/EsY/NiR** system, the energy transfer efficiency were determined to be 89.3%, when the ratio of **PCP-TPy1/EsY** and NiR reached 2000:10:10 (Fig. 7d), which is much higher than those of the reported one-step or other sequential energy transfer systems^80–89^ (Supplementary Table 3 and 4).

Similarly, ***R*p,*R*p-PCP-Tpy1** was also used to construct one-step or sequential ALHS with EsY and NiR. ***R*p,*R*p-PCP-Tpy1** exhibits a similar energy transfer ability to **PCP-TPy1** (Supplementary Fig. 35). The energy transfer efficiency and antenna effect were calculated to be 83.8% and 10.9, respectively, for the system of ***R*p,*R*p-PCP-Tpy1** and EsY at the ratio of 200:1 excited at 360 nm (Supplementary Fig. 36). In ***R*p,*R*p-PCP-Tpy1/NiR** system, the energy transfer efficiency and antenna effect were calculated to be 73.3% and 15.4, respectively, for the system of ***R*p,*R*p-PCP-Tpy1** and NiR under the same conditions (Supplementary Fig. 36 and Supplementary Table 3). In sequential energy transfer system, the energy transfer efficiency of ***R*p,*R*p-PCP-TPy1/EsY/NiR** was calculated to be 87.8%, when the ratio of ***R*p-PCP-TPy1/EsY** and NiR reached 2000:10:10 (Supplementary Fig. 36 and Supplementary Table 4). All the evidences indicated that ***R*p,*R*p-PCP-Tpy1** displayed the similar energy transfer ability to **PCP-TPy1**.

The double helicate **PCP-TPy2** was also utilized as an energy donor to build ALHS. As shown in Fig. 6j, with the addition of NiR into the solution (THF/water=1:9, v/v) of **PCP-TPy2**, the emission of **PCP-TPy2** (530 nm) decreased and that of NiR (612 nm) increased. When the ratio between **PCP-TPy2** and NiR reached 200:1, the emission intensity of this system was almost constant (Fig. 6k). This system experienced a change of emission color from yellow to orange as the increase of NiR fractions (Fig. 6l). Similarly, ***R*p,*R*p-PCP-Tpy2** and NiR also could construct a one-step ALHS (Supplementary Fig. 38). These ALHSs showed excellent energy transfer efficiency (74.8% and 79.9%) and perfect antenna effect (13.5 and 18.9) when the ratio of donor (**PCP-Tpy2** and ***R*p,*R*p-PCP-Tpy2**) and acceptor (NiR) reached 200:1 (Supplementary Figs. 37 and 38, Supplementary Table 3). Additionally, SEM and DLS measurements demonstrated that the introduction of energy accepters into the ALHS did not obviously change the morphologies and sized of nanoaggregates (Supplementary Figs. 40–45).

**Fig. 8:**
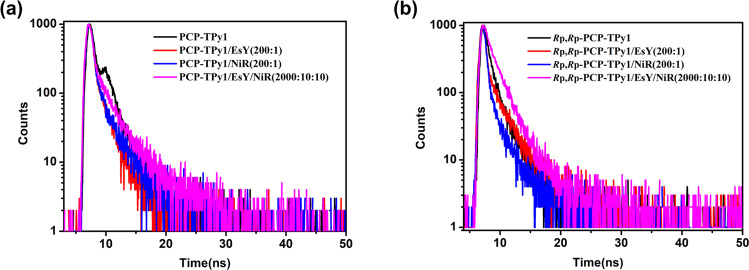
Fluorescence decay experiments. (**a**) Changes in the fluorescence decay profiles of the **PCP-TPy1, PCP-TPy1/EsY (200:1), PCP-TPy1/NiR (200:1)** and **PCP-TPy1/EsY/NiR (2000:10:10)** in THF/Water (1:9; v/v), (**b**) Changes in the fluorescence decay profiles of the ***R*p,*R*p-PCP-TPy1,** ***R*p,*R*p-PCP-TPy1/EsY (200:1),** ***R*p,*R*p-PCP-TPy1/NiR (200:1)** and ***R*p,*R*p-PCP-TPy1/EsY/NiR (2000:10:10)** in THF/Water (1:9; v/v).

The energy transfer process was further confirmed by fluorescence decay experiments. The fluorescence lifetimes were summarized in Table 1 The double helicate **PCP-TPy1** showed a fluorescence lifetime of *τ* = 2.09 ns at 495 nm, which was changed to *τ* = 2.30 and 1.80 ns in the presence of EsY and NiR, respectively (Fig. 8a and Table 1). The lifetime of **PCP-TPy1** in the sequential energy transfer system **PCP-TPy1/EsY/NiR** was found to be 2.16 ns (Fig. 8a and Table 1). The fluorescence lifetimes at 530 nm of the double helicate **PCP-TPy2** were detected to be τ = 1.25 and 0.64 ns in the absence and presence of NiR, respectively (Supplementary Fig. 46 and Supplementary Table 5). The fluorescence decays of ALHSs constructed by chiral double helicates ***R*p,*R*p-PCP-Tpy1/2** similar to ALHSs built by **PCP-TPy1/2** (Fig. 8b and Supplementary Fig. 46, Table 1 and Supplementary Table 5). The obvious changes on the fluorescence lifetimes clearly demonstrate that the energy is successfully transferred from the donor to acceptor, therefore, one can conclude that supramolecular ALHSs in the present studies are constructed successfully.

**Table. 1:** Fluorescence lifetimes of PCP-TPy1 and *R*p,*R*p-PCP-TPy2 in aggregated states and their ALHSs

Systems	τ/ns	Systems	τ/ns
PCP-TPy1	2.09 ± 0.23	*R*p,*R*p-PCP-TPy1	1.82 ± 0.22
PCP-TPy1/EsY (200:1)	2.30 ± 0.20	*R*p,*R*p-PCP-TPy1/EsY (200:1)	2.32 ± 0.19
PCP-TPy1/NiR (200:1)	1.80 ± 0.21	*R*p,*R*p-PCP-TPy1/NiR (200:1)	1.75 ± 0.25
PCP-TPy1/EsY/NiR (2000:10:10)	2.16 ± 0.23	*R*p,*R*p-PCP-TPy1/EsY/NiR (2000:10:10)	1.98 ± 0.33

In order to shed light on the vital role of double helicates in constructing ALHSs, the building block **TPy1** or **TPy2** itself was used to fabricate ALHSs. Although **TPy1** and **TPy2** also display AIE properties, their fluorescence intensities in the aggregation state are much weaker than those of double helicates **PCP-Tpy1 and PCP-TPy2** under the same conditions (Supplementary Figs. 47–49). Unfortunately, our attempts to construct ALHSs from the building blocks **TPy1** or **TPy2** and fluorescence dyes failed because no efficient energy transfer from aggregated **TPy1** and **TPy2** to dyes including EsY and NiR was observed (Supplementary Fig. 50). These results clearly demonstrate that the formation of double helicate is the key to construct ALHSs.

### Chiroptical properties of double helicates

The chiroptical properties of chiral double helicates ***R*p,*R*p-PCP-Tpy1** and ***R*p,*R*p-PCP-Tpy2** were further evaluated by CD and CPL spectroscopies. The CD spectra of ***R*p,*R*p-PCP-Tpy1** and ***R*p,*R*p-PCP-Tpy2** exhibit the similar evident Cotton effects at 341 and 384 nm in pure THF solvent (Supplementary Fig. 51). The absorption dissymmetry factors |*g*_*abs*_| were evaluated to be 2.0 × 10^−3^ for ***R*p,*R*p-PCP-Tpy1** and 2.4 × 10^−3^ for ***R*p,*R*p-PCP-Tpy2** (Supplementary Table 6). CPL investigations revealed that ***R*p,*R*p-PCP-Tpy1** and ***R*p,*R*p-PCP-Tpy2** emitted a weak CPL with maxima located at 450 nm (Supplementary Fig. 51). The CPL dissymmetry factors |*g*_lum_| of ***R*p,*R*p-PCP-Tpy1** and ***R*p,*R*p-PCP-Tpy2** in pure THF solvent were estimated to be 1.7 × 10^−3^ and 2.4 × 10^−3^ (Supplementary Table 6). Furthermore, the CD and CPL properties of chiral double helicates in aggregate states were also investigated. Unexpectedly, the values of |*g*_*abs*_| and |*g*_lum_| of ***R*p,*R*p-PCP-Tpy1/2** in the mixture of THF/water are comparable with those in pure THF solvent (Supplementary Fig. 52 and Supplementary Table 6). No significant increase in the dissymmetry factors were observed for the aggregate states, indicating that the chirality of the planar chiral [2.2]PCP building block in double helicates was not efficiently transferred to supramolecular chirality, which is partially accounted by the spherical morphologies in the aggregate states.

### Light-emitting device of double helicates

**Fig. 9:**
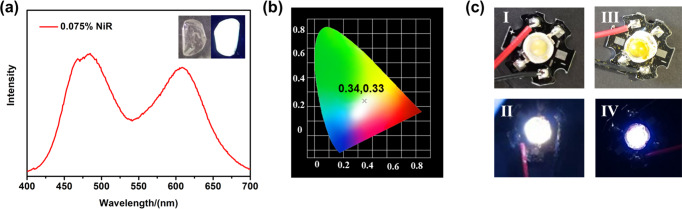
Potential application of novel double helicates. Fluorescence emission spectrum (**a**) of **PCP-TPy1** film with 0.075% molar percentages NiR (λ_ex_ = 360 nm, slit widths: ex = 5 nm, em = 5 nm); (**b**) The CIE point of **PCP-TPy1** film with 0.075% NiR; (**c**) LED device of **PCP-TPy2** (I) (II); LED device of ***R*p,*R*p-PCP-TPy2** (III) (IV).

The PMMA films of **PCP-TPy1** and **PCP-TPy2** were constructed to investigate emission in the solid state. Under a 365 nm UV light, the PMMA films of **PCP-TPy1** and **PCP-TPy2** show green and yellow emissions, respectively (Supplementary Fig. 53). Interestingly, the PMMA film of **PCP-TPy1** displays white-light emission when doped 0.075% NiR. As shown in Fig. 9a, two emission bands centered at 485 nm and 610 nm were found from the emission spectrum, which covers the visible region and locates at the white-light zone with CIE value of (0.34, 0.33) (Fig. 9b). However, the PMMA film of **PCP-TPy2** with a doping percentage of 0.075% NiR displays yellow-light emission (Supplementary Fig. 54). When doped 0.5% NiR, the PMMA films of **PCP-TPy1** and **PCP-TPy2** show pink and orange emissions, respectively (Supplementary Fig. 55). These fluorescent films may have great potential application in optoelectronic devices. Furthermore, the solid of **PCP-TPy2** exhibits a high yellow luminescence that can be excited by blue light and thus can serve as a high- performance emitter for white LED^90^. Therefore, by integrating the **PCP-TPy2** with a 460 nm LED chip, a double helicate based white LED device was fabricated. This LED chip grow bright white light, when a 3 V bias is applied (Fig. 9c). However, under the same conditions, the **PCP-TPy1** based LED still emits blue light (Supplementary Fig. 56). The solid of ***R*p,*R*p-PCP-TPy2** also could be fabricated white LED device (Fig. 9c), indicating that powder of ***R*p,*R*p-PCP-TPy2** may have potential applications in CPL-OLED light-emitting devices in the future.

In summary, two double helicates and their chiral counterparts were designed and synthesized by using 60° diplatinum(II) complex ***rac*/*R*p-PCP** and 120° dipyridyl TPE ligands TPy1/TPy2. Double helicates **PCP-TPy1** and **PCP-TPy2** display superior AIE property to their precursors **TPy1/TPy2** and can be used to construct single or sequential energy transfer system doped with EsY and NiR with excellent energy transfer efficiency (89.3%). Chiral double helicates ***R*p,*R*p-PCP-TPy1/2** also demonstrated excellent AIE performance and efficient energy transfer capacity. Noticeably, due to the solid-state emission of double helicates, **PCP-TPy1** can be made into white-light film, (chiral) **PCP-TPy2** can be loaded by a blue LED bulb to prepare white LEDs. The results indicate that these double helicates may have great potential application in optoelectronic devices. This work not only provides a unique strategy for the constructing novel double helicates and artificial ALHSs, but also offers prospects for potential applications of double helicates in solid state as fluorescent materials, in particular, chiral fluorescent materials, which is an ongoing project in our group.

## Methods

All starting chemicals were obtained from commercial sources and used without further purification, unless indicated otherwise. All reactions were performed with dry solvents under Argon in dried glassware with standard vacuum-line techniques. Anhydrous THF and dichloromethane were obtained from Solvent Purification System. Glassware used for water-free reactions were dried for 12 h at 120 °C before use. The ^1^H NMR spectra, ^13^C NMR and ^31^P NMR were recorded on JEOL Delta (400 MHz and 600 MHz) spectrometer at 298 K, using chloroform-d (CDCl_3_) as solvent. The chemical shift references were as follows: (^1^H) chloroform-*d*, 7.26 ppm; (^13^C) chloroform-*d*, 77.00 ppm (chloroform-*d*). High-resolution mass spectral (HRMS) data were obtained on an electrospray (ESI) mass spectrometer analyzing time-of-flight. UV-vis spectra were recorded on UV-2450 spectrophotometer. Fluorescence spectra were measured were obtained using and FS5 fluorescence spectrophotometer. The absolute fluorescence quantum yield was measured by using an absolute PL quantum yield spectrometer (Edinburg FLS-980 fluorescence spectrometer). Scanning electron microscopy (SEM) was performed on a Hitachi SU-8010 with an accelerating voltage of 10 kV.

### Data analysis

All plotted and calculated statistical analyses were performed on Origin 8.5.

### Synthesis of 1

Synthesis of **0** was performed according to the literature^74^. A mixture of compound **0** (100 mg, 0.16 mmol), Pd_2_(dba)_3_ (16.8 mg, 0.016 mmol), dppf (9.0 mg, 0.016 mmol), CuI (3.0 mg, 0.0016 mmol), THF (15 ml) and Et_3_N (15 ml) were placed in a round-bottom flask equipped with a magnetic stirring bar. After degassing the reaction mixture several times, 4-Iodobenzotrifluoride (160 mg, 0.64 mmol) was added to the mixture via syringe. The reaction was carried out at 65 °C for 1 d with stirring. After the reaction mixture was cooled to room temperature, precipitates were removed by filtration, and the solvent was removed with a rotary evaporator. The residue was purified by column chromatography on SiO_2_ (hexane as an eluent) to afford compound **1** as a light yellow solid (93 mg, 63%). ^1^H NMR (600 MHz, CDCl_3_, 298 K) *δ* 7.65 (s, 8H), 7.09 (s, 2H), 7.07 (s, 2H), 3.57–3.48 (m, 4H), 3.13–2.98 (m, 4H), 1.20 (q, *J* = 1.5 Hz, 42H). ^13^C NMR (150 MHz, CDCl_3_, 298 K) *δ* 142.6, 142.3, 134.8, 134.6, 131.7, 127.4, 125.9, 125.5, 125.5, 125.5, 124.3, 105.8, 96.5, 93.0, 91.5, 32.8, 32.3, 18.9, 18.9, 11.5. HR-MS (MALDI-TOF) calcd. for C_56_H_62_F_6_Si_2_ M: 904.4294, found 904.3230.

### Synthesis of 3

Compound **1** (120 mg, 0.13 mmol) was dissolved in THF (10 mL), followed by the addition of Bu_4_NF (1.0 M THF solution, 1.8 mL). The reaction was carried out at room temperature for 5 min. H_2_O was added to the reaction mixture. The organic layer was extracted three times with CH_2_Cl_2_ and washed with brine, and dried over MgSO_4_. MgSO_4_ was removed by filtration, and the solvent was removed by a rotary evaporator to get compound **2** as a dark yellow oil.

To a dichloromethane solution of compound **2** and Pd(PEt_3_)_2_I_2_ (380 mg, 0.54 mmol) was added a catalytic amount of CuI (14.2 mg, 0.080 mmol), then dry diethylamine solution was added, the reaction mixture was stirred at room temperature for 12 h. The resulting ammonium salt was removed by filtration, and the filtrate was concentrated under reduced pressure. The residue was purified by column chromatography on SiO_2_ (PE/EA = 5/1 v/v as an eluent) to obtain compound **3** as a light yellow solid (150 mg, 66%). ^1^H NMR (400 MHz, CDCl_3_, 298 K) *δ* 7.64 (s, 8H), 7.05 (s, 2H), 6.69 (s, 2H), 3.59 – 3.39 (m, 4H), 2.94 (m, 4H), 2.23 (ddq, *J* = 11.0, 7.3, 4.1 Hz, 18H), 2.12–2.03 (m, 6H), 1.23 – 1.15 (m, 36H). ^13^C NMR (100 MHz, CDCl_3_, 298 K) *δ* 142.0, 139.9, 134.3, 133.6, 131.5, 130.8, 128.0, 125.4, 125.4, 122.7, 120.6, 100.8, 92.7, 91.9, 33.0, 32.8, 17.0, 16.8, 16.6, 8.4. ^31^P NMR (148 MHZ, CDCl_3_, 298 K) *δ* 9.72 (^1^*J*_Pt-P_ = 2134.16 Hz). MS (ESI-MS): *m*/*z* calcd. for [M – 2I]^2+^: 726.2205, found: 726.0311.

### Synthesis of *rac*-PCP

A 35 mL round-bottom Schlenk flask was charged with compound **3** (60 mg, 0.035 mmol) and 10 mL of dichloromethane, then the solution was added AgNO_3_ (60 mg, 0.35 mmol) at once, resulting in a yellowish precipitate of AgI. After 12 h at room temperature, the suspension was filtered through a glass fiber and the volume of the solution reduced to 3 mL. Subsequent addition of diethyl ether resulted in the precipitation of the compound ***rac*-PCP** as a slightly yellow crystalline powder (50 mg, 90%). ^1^H NMR (600 MHz, CDCl_3_, 298 K) *δ* 7.63 (s, 8H), 7.05 (s, 2H), 6.65 (s, 2H), 3.54–3.39 (m, 4H), 2.99 (dd, *J* = 6.6, 2.5 Hz, 2H), 2.87 (dd, *J* = 6.6, 2.7 Hz, 2H), 2.01 – 1.95 (m, 24H), 1.27–1.24 (m, 36H). ^13^C NMR (100 MHz, CDCl_3_, 298 K) *δ* 141.9, 140.5, 134.5, 133.7, 131.6, 130.0, 129.7, 129.5, 127.6, 125.5, 125.5, 125.4, 125.4, 92.5, 91.9, 33.0, 32.6, 14.8, 14.6, 14.5, 7.9. ^31^P NMR (242 MHZ, CDCl_3_, 298 K) *δ* 20.87 (^1^*J*_Pt-P_ = 2483.46 Hz). MS (ESI-MS): *m/z* calcd. for [M – 2NO_3_]^2+^: 726.2205, found: 726.0843.

### Synthesis of PCP-TPy1

The Compound ***rac*-PCP** (25 mg, 0.016 mmol) and **TPy1** (8.0 mg, 0.016 mmol) were weighed accurately into a glass vial. To the vial were added 10 mL of acetone and 10 ml of dichloromethane, and the reaction solution was then stirred at 60 °C for 12 h to yield a homogeneous yellow solution. Then the addition of a saturated aqueous solution of KPF_6_ into the bottle with continuous stirring (10 min) precipitated the product. The reaction mixture was centrifuged, washed several times with water, and dried. Gray solid product of helicate **PCP-TPy1** was obtained by removing the solvent under vacuum (30 mg, 90%). ^1^H NMR (600 MHz, CDCl_3_, 298 K) *δ* 8.66 (d, *J* = 5.9 Hz, 4H), 7.90 (d, *J* = 6.2 Hz, 4H), 7.65 (s, 8H), 7.56 (dd, *J* = 8.3, 2.4 Hz, 4H), 7.20 – 7.14 (m, 10H), 7.10 (s, 6H), 6.67 (d, *J* = 3.9 Hz, 2H), 3.63–3.40 (m, 4H), 3.04 (s, 2H), 2.85 (s, 2H), 1.83 (s, 24H), 1.25–1.20 (m, 36H). ^13^C NMR (150 MHz, CDCl_3_, 298 K) *δ* 152.5, 151.2, 146.4, 142.8, 141.8, 140.3, 134.4, 134.2, 133.0, 132.5, 131.6, 131.3, 129.8, 129.2, 128.1, 127.6, 127.3, 127.0, 125.5, 125.5, 125.3, 122.0, 92.6, 91.9, 33.2, 32.7, 14.6, 14.5, 14.4, 8.8, 8.0. ^31^P NMR (242 MHZ, CDCl_3_, 298 K) *δ* 16.01(^1^*J*_Pt-P_ = 2320.53 Hz). MS (ESI-MS): *m/z* calcd. for [M–2PF_6_]^2+^: 2084.9582, found: 2084.6128; *m*/*z* calcd. for [M–3PF_6_]^3+^: 1341.6205, found: 1341.4221; *m/z* calcd. for [M–4PF_6_] ^4+^: 969.9967, found: 969.8248.

### Synthesis of PCP-TPy2

The Compound ***rac*-PCP** (26 mg, 0.016 mmol) and **TPy2** (9.0 mg, 0.016 mmol) were weighed accurately into a glass vial. To the vial were added 10 mL of acetone and 10 ml of dichloromethane, and the reaction solution was then stirred at 60 °C for 12 h to yield a homogeneous yellow solution. Then the addition of a saturated aqueous solution of KPF_6_ into the bottle with continuous stirring (10 min) precipitated the product. The reaction mixture was centrifuged, washed several times with water, and dried. Yellow solid product of helicate **PCP-TPy2** was obtained by removing the solvent under vacuum (29 mg, 82%). ^1^H NMR (600 MHz, CDCl_3_, 298 K) *δ* 8.65 (d, *J* = 5.9 Hz, 4H), 7.90 (d, *J* = 6.0 Hz, 4H), 7.65 (s, 8H), 7.56 (dd, *J* = 8.5, 2.9 Hz, 4H), 7.18 (d, *J* = 8.1 Hz, 4H), 7.10 (s, 2H), 7.01 (d, *J* = 8.6 Hz, 4H), 6.78 – 6.61 (m, 6H), 3.77 (s, 6H), 3.51 (d, *J* = 45.0 Hz, 4H), 3.04 (s, 2H), 2.86 (s, 2H), 1.84 (s, 24H), 1.23 (t, *J* = 8.4 Hz, 36H). ^13^C NMR (150 MHz, CDCl_3_, 298 K) *δ* 158.8, 152.4, 151.3, 147.1, 143.0, 141.8, 140.3, 135.4, 134.4, 134.2, 132.7, 132.6, 132.6, 131.6, 129.2, 127.6, 127.1, 125.5, 125.5, 125.2, 124.9, 123.1, 122.0, 113.4, 92.6, 91.9, 69.6, 55.2, 53.8, 33.2, 32.7, 31.8, 29.3, 14.6, 14.5, 14.4. ^1^P NMR (242 MHZ, CDCl_3_, 298 K) *δ* 16.04(^1^*J*_Pt-P_ = 2320.51 Hz). MS (ESI-MS): *m*/*z* calcd. for [M–2PF_6_]^2+^: 2145.0102, found: 2144.6418; *m*/z calcd. for [M–3PF_6_]^3+^: 1381.6852, found: 1381.7699; *m*/z calcd. for [M – 4PF_6_] ^4+^: 1000.0227, found: 999.8371.

### Method for sample preparation of double helicates in THF/H2O solution

Take a 10 ml screw bottle and add 50ul of the THF solution of **PCP-TPy1** (1 × 10^−3^ M) to it. The bottle is then drained and the calculated THF solution is added to the bottle and shaken well. Finally, the corresponding water was quickly added to the bottle, and the screw bottle was ultrasonic for 5 min. **PCP-TPy1** (1 × 10^−5^ M) samples were prepared in THF solutions with different water contents.

### Theoretical calculation

All calculations were performed using the Gaussian 16 program package. The geometries of ***meso*-PCP-TPy1** and ***R*p,*R*p-PCP-TPy1** were optimized at the B3LYP level of density functional theory (DFT). For the Pt atom, we adopted the corresponding ECP60MWB (8s7p6d)/[6s5p3d] basis set, the all-electron 6–31G* basis sets were applied for the rest atoms of C, P, N and H atoms. The corresponding pseudopotential parameters and basis sets developed by Dolg’s group are also available from the website, http://www.tc.uni-koeln.de/PP/clickpse.en.html. Harmonic vibration frequency calculations at the same level were performed to verify all stationary points as local minima (with no imaginary frequency). On the basis of optimizing the convergence structure, use PCM for single point energy correction.

## Supplementary information

Supplementary Information

## Data Availability

The data generated in this study are available within the article, Supplementary Information, and Source Data. Source data is available for Figs. 2, 3, 6, 7 and 8 and Supplementary Figs. 9–12,51 and 52 in the associated source data file. Source data are provided with this paper.

## Source data

Source Data
