# Supplementary material for: [2,2] Paracyclophanes-based double helicates for constructing artificial light-harvesting systems and white LED device
Source: Nat Commun. 2023 May 12;14:2752. doi: 10.1038/s41467-023-38405-9 (PMC10182020; doi:10.1038/s41467-023-38405-9)
Supplement: Supplementary file 1 — Supplementary Information [file 41467_2023_38405_MOESM1_ESM.pdf]

Supplementary Information

[2,2] Paracyclophanes-Based Double Helicates for Constructing Artificial Light-harvesting Systems and White LED Device

Zhe Lian[1], Jing He[1], Lin Liu[1], Yanqing Fan[1], Xuebo Chen[1], and Hua Jiang[1]*

[1]College of Chemistry, Beijing Normal University, Beijing 100875, P.R. China.

Correspondence and requests for materials should be addressed to H. Jiang. (email: jiangh@bnu.edu.cn).

Table of Contents

1. Supplementary Figures **3**

2. Supplementary Tables **36**

3. Supplementary Methods **46**

4. Supplementary References **48**

1. Supplementary Figures

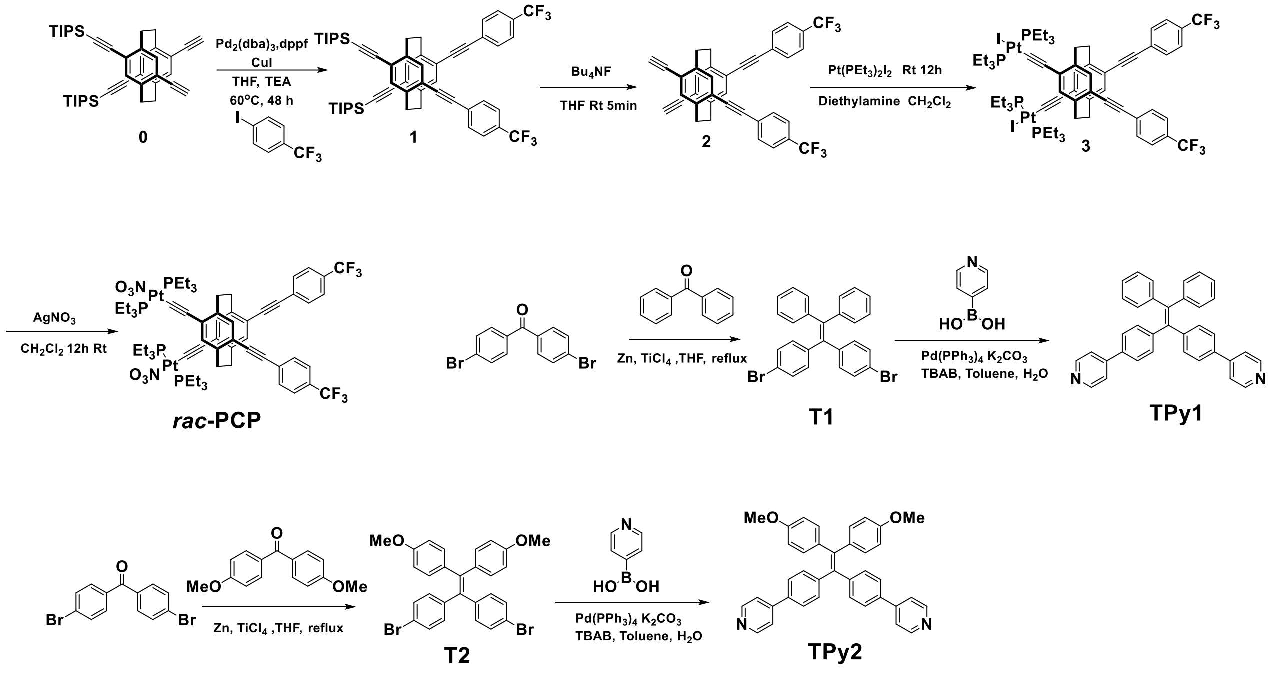

Supplementary Fig 1. Synthetic routine of *rac*-PCP, TPy1 and TPy2

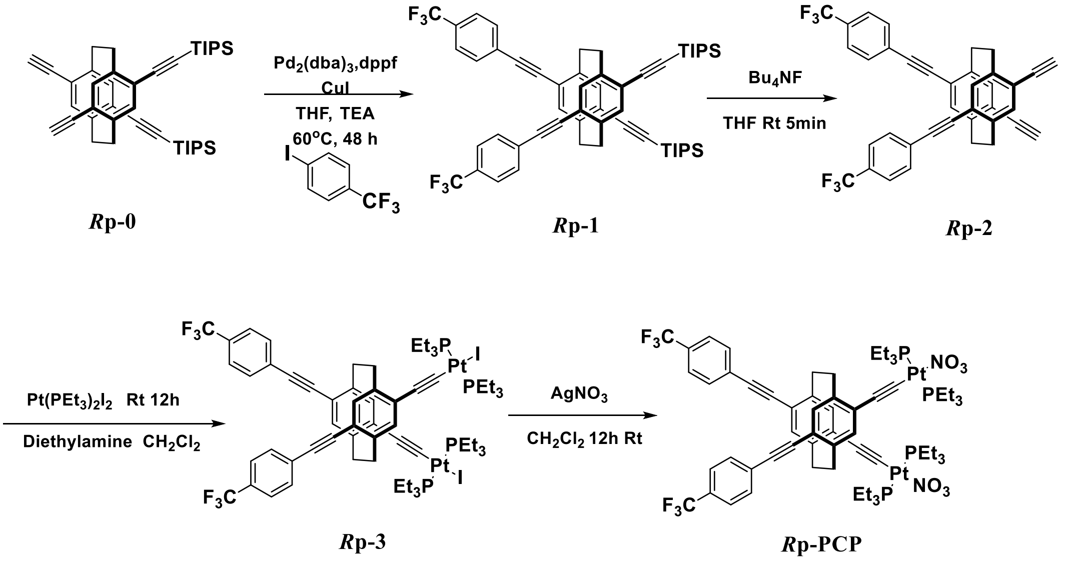

Supplementary Fig 2. Synthetic routine of *R*p-PCP

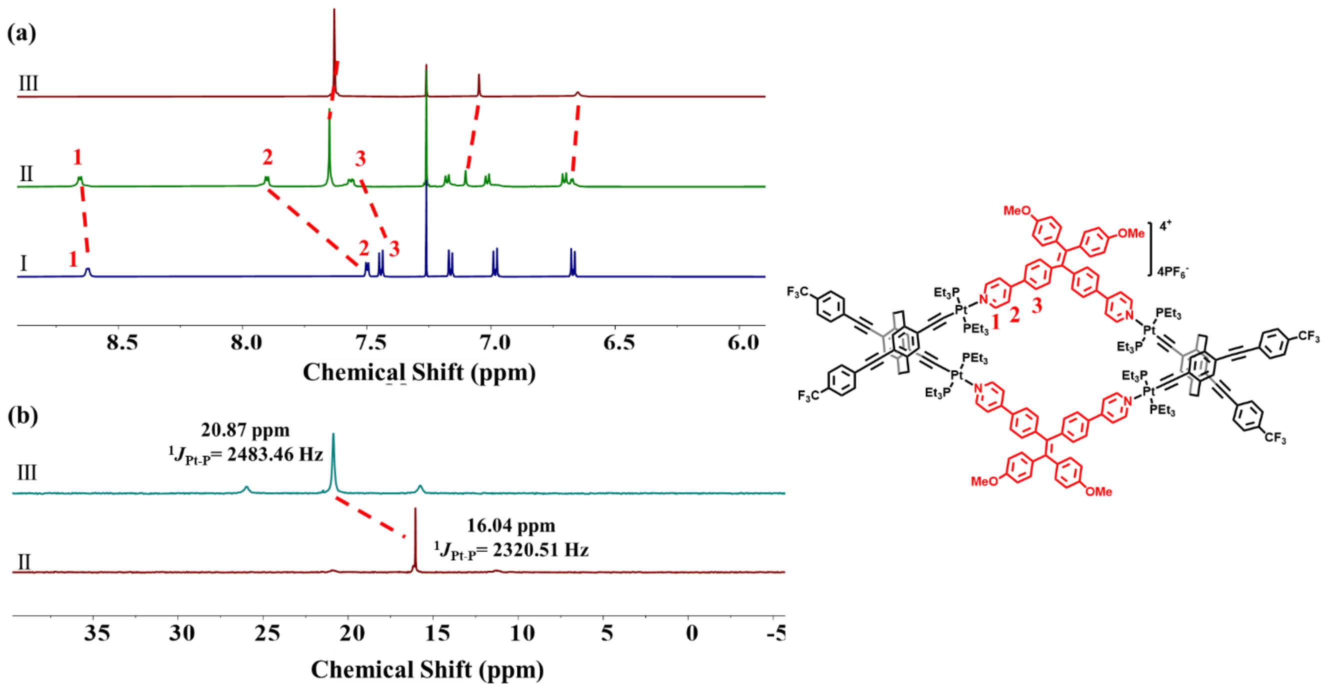

Supplementary Fig 3. Partial ^{1}H NMR spectra (600 M, $CDCl_3$, 298 K) of Tpy2 (a-I), PCP-TPy2 (a-II) and *rac*-PCP (a-III). ^{31}P NMR spectra (242 M, $CDCl_3$, 298 K) of double helicate PCP-TPy2 (b-II) and *rac*-PCP (b-III).

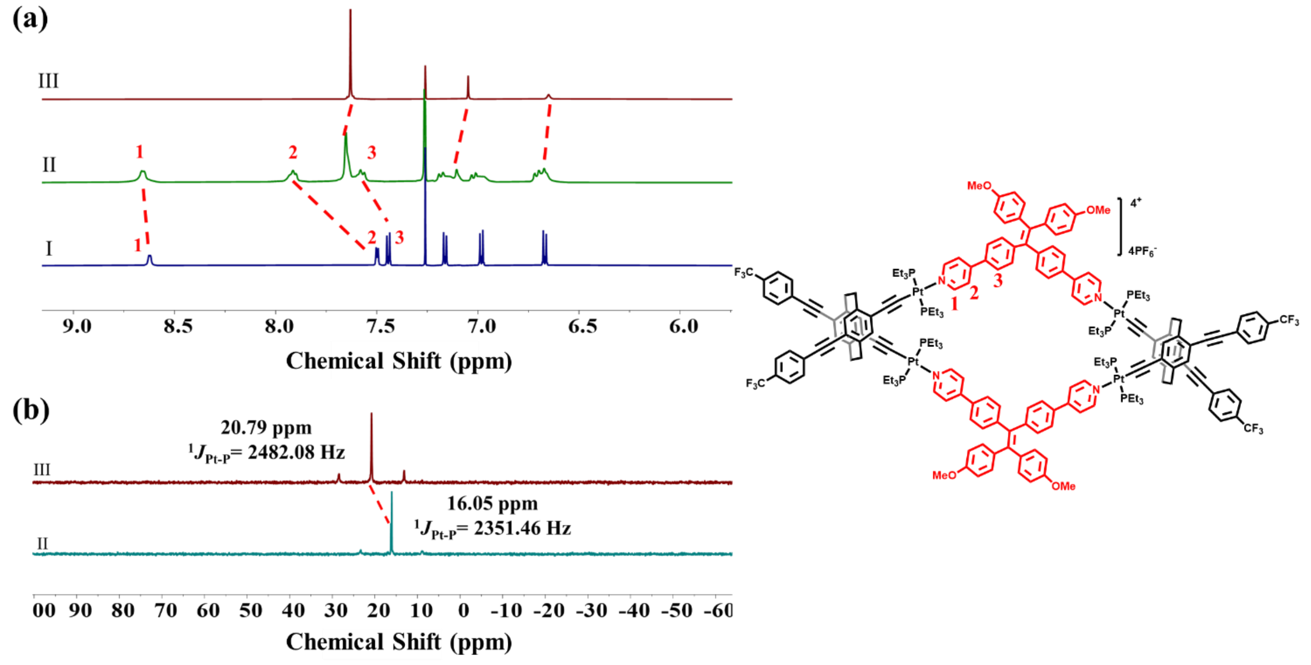

Supplementary Fig 4. Partial ^{1}H NMR spectra (400 M, $CDCl_3$, 298 K) of TPy2 (a-I), *R*p,*R*p-PCP-TPy2 (a-II) and *R*p-PCP (a-III). ^{31}P NMR spectra (162 M, $CDCl_3$, 298 K) of double helicate *R*p,*R*p-PCP-TPy2 (b-II) and *R*p-PCP (b-III).

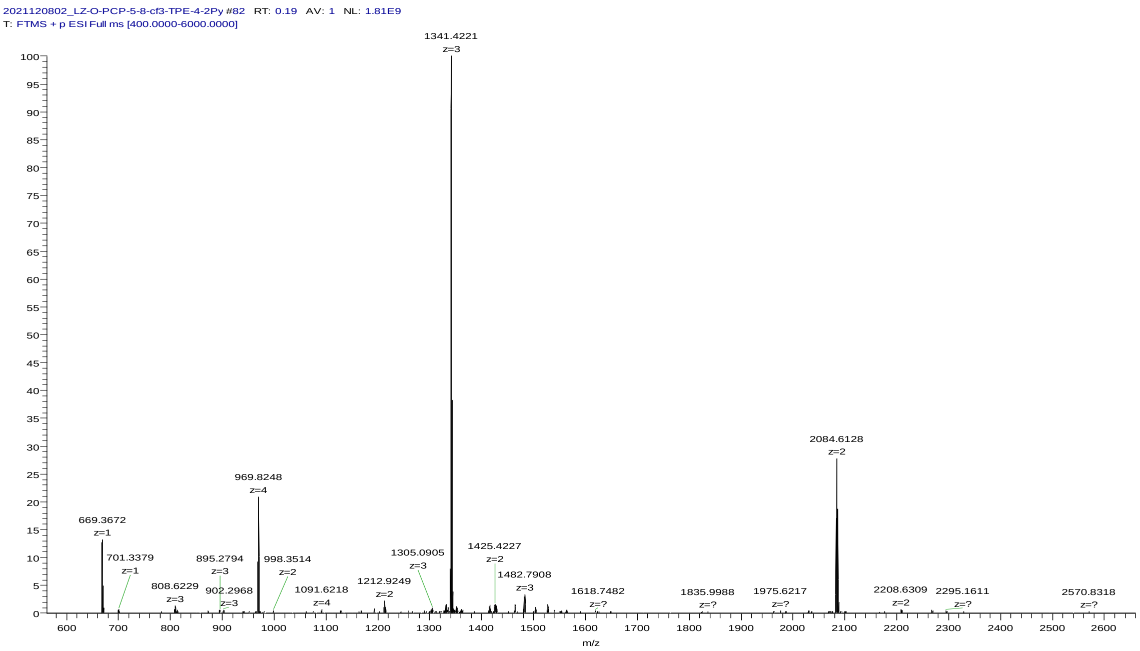

Supplementary Fig 5. Electrospray ionization mass spectrum of PCP-TPy1

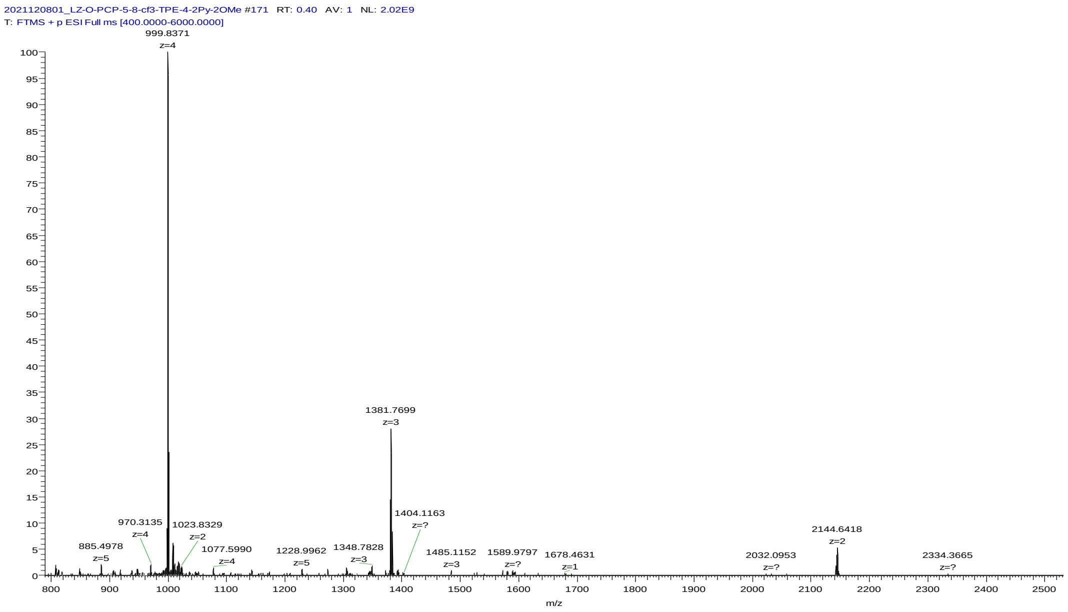

Supplementary Fig 6. Electrospray ionization mass spectrum of PCP-TPy2

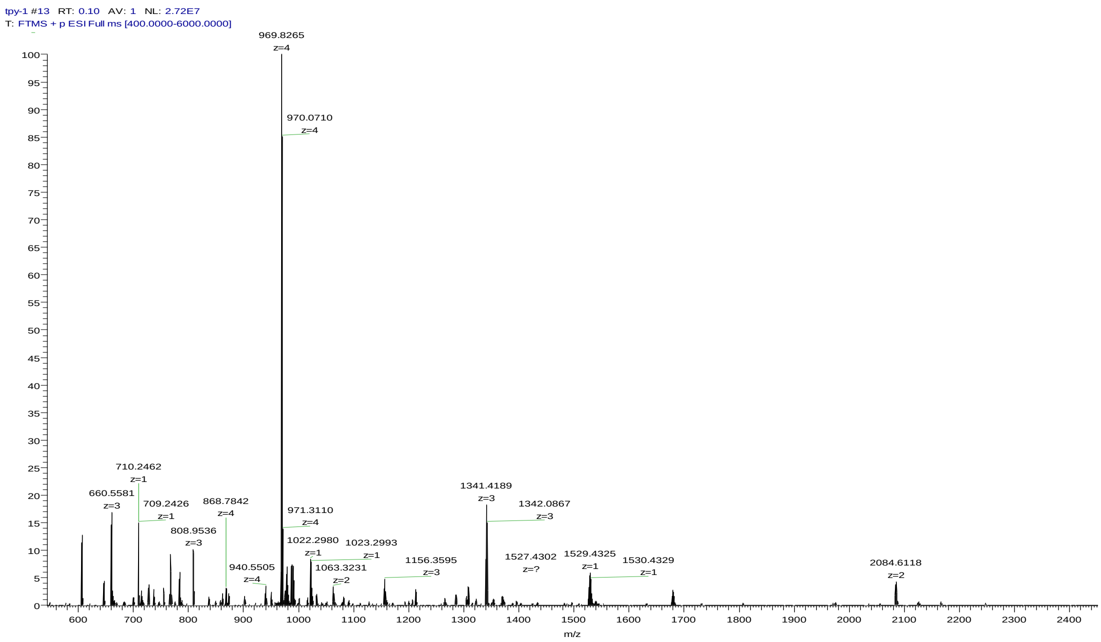

Supplementary Fig 7. Electrospray ionization mass spectrum of *R*p,*R*p-PCP-TPy1

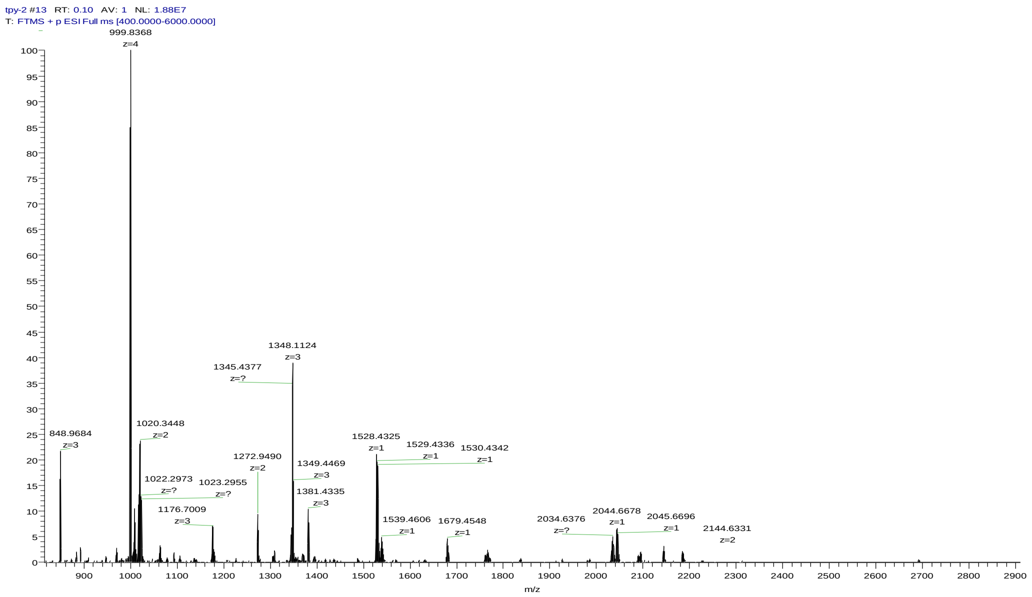

Supplementary Fig 8. Electrospray ionization mass spectrum of *R*p,*R*p-PCP-TPy2

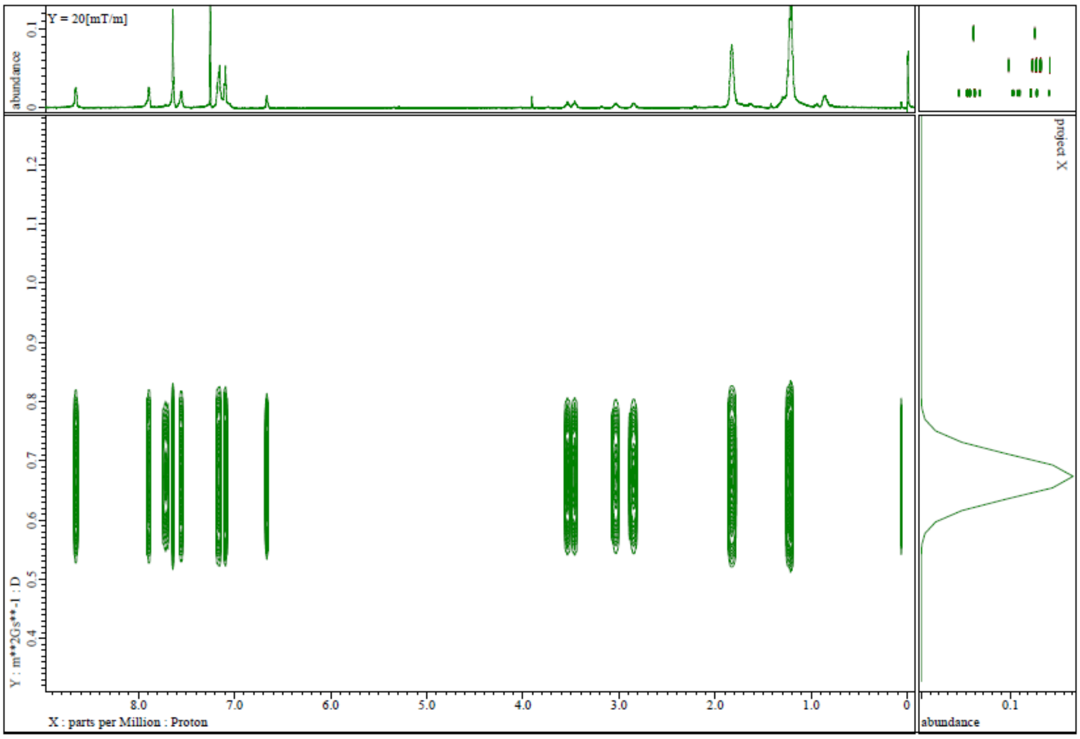

Supplementary Fig 9. 2D DOSY NMR (600 MHz, $CDCl_3$, 298 K) spectrum of double helicate PCP-TPy1

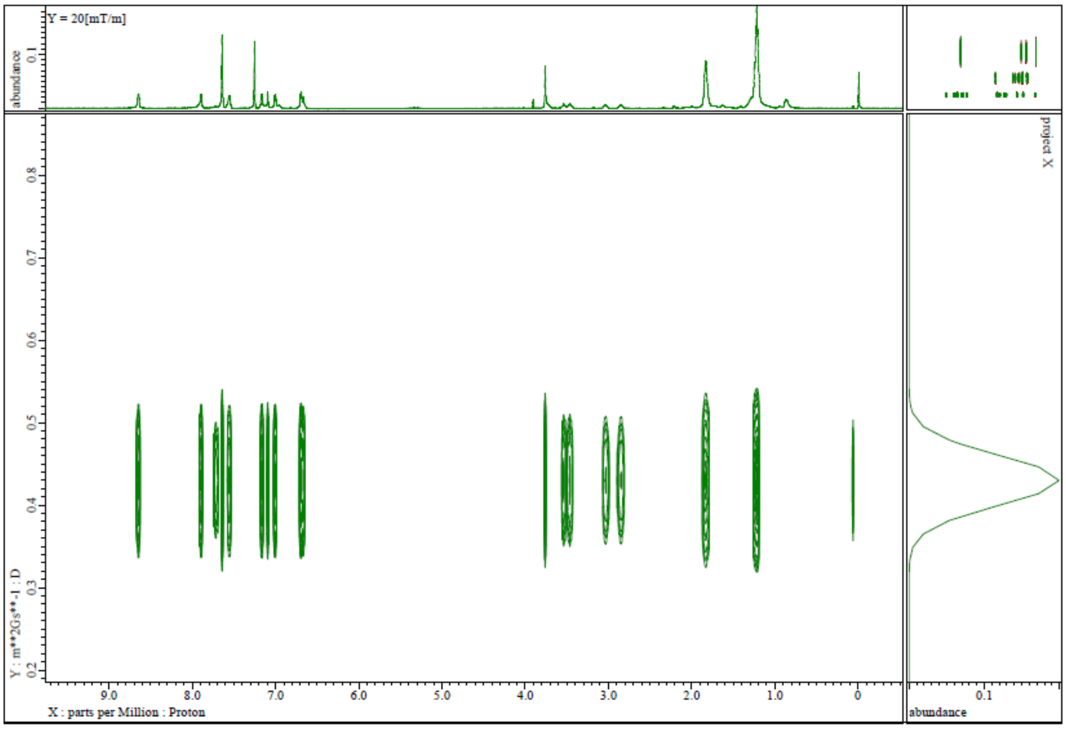

Supplementary Fig 10. 2D DOSY NMR (600 MHz, $CDCl_3$, 298 K) spectrum of double helicate PCP-TPy2

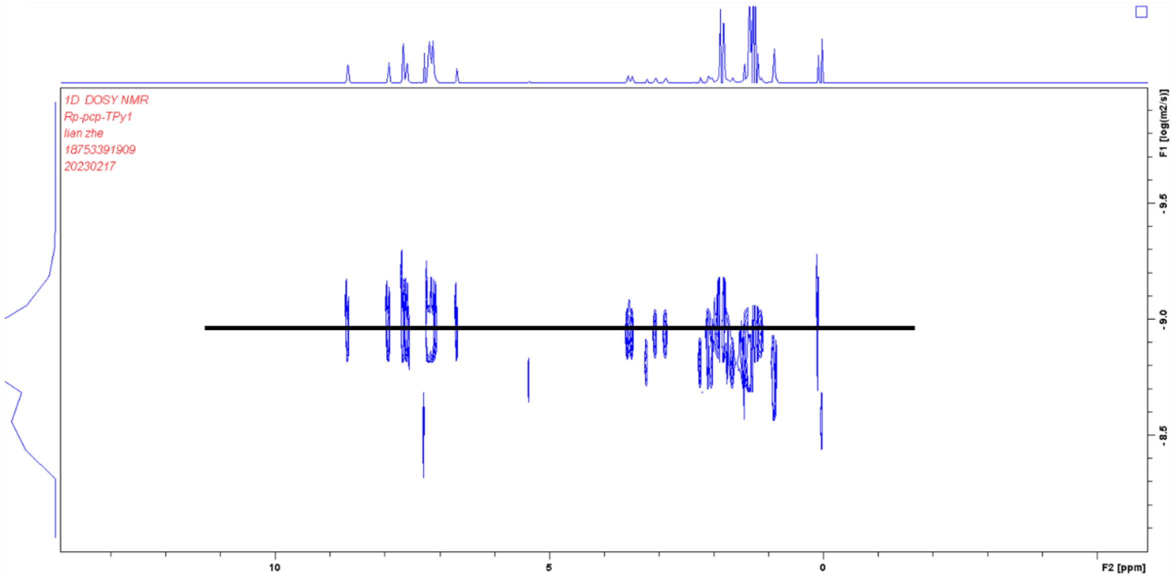

Supplementary Fig 11. 2D DOSY NMR (400 MHz, $CDCl_3$, 298 K) spectrum of double helicate *R*p,*R*p-PCP-TPy1

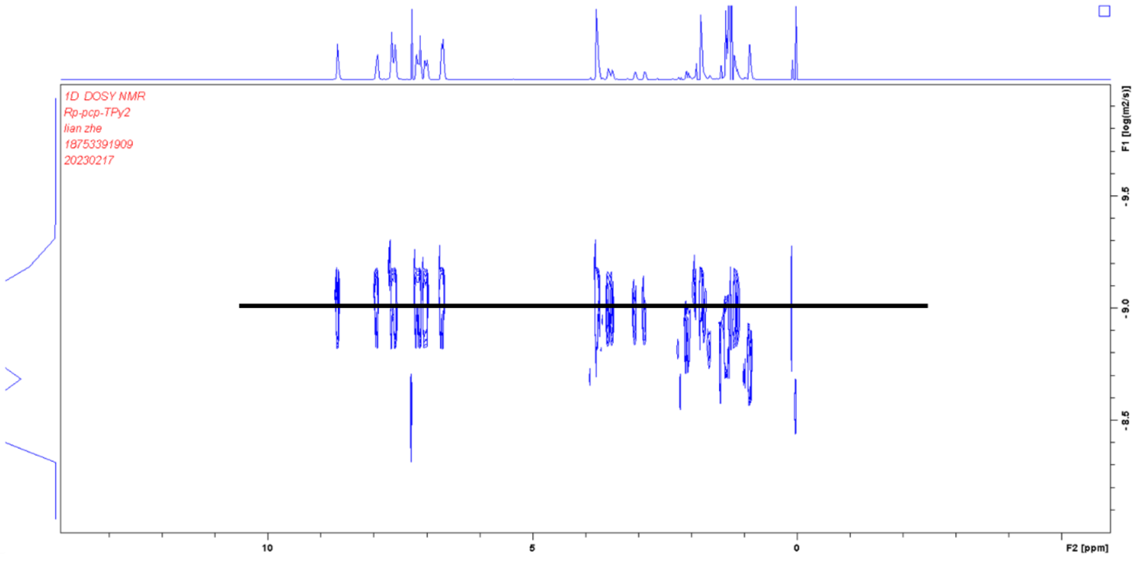

Supplementary Fig 12. 2D DOSY NMR (400 MHz, $CDCl_3$, 298 K) spectrum of double helicate *R*p,*R*p-PCP-TPy2

Theoretical calculations were performed using the Gaussian 16[5] program package. All calculations were carried out using the density functional theory (DFT) method. Harmonic vibration frequency calculations at the same level were performed to verify all stationary points as local minima (with no imaginary frequency).

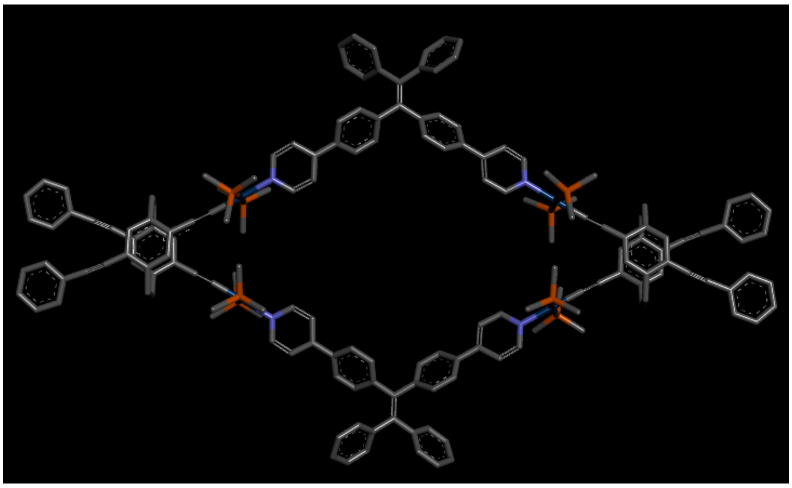

Supplementary Fig 13. Gaussian 16 optimized structure of the double helicate *meso*-PCP-TPy1

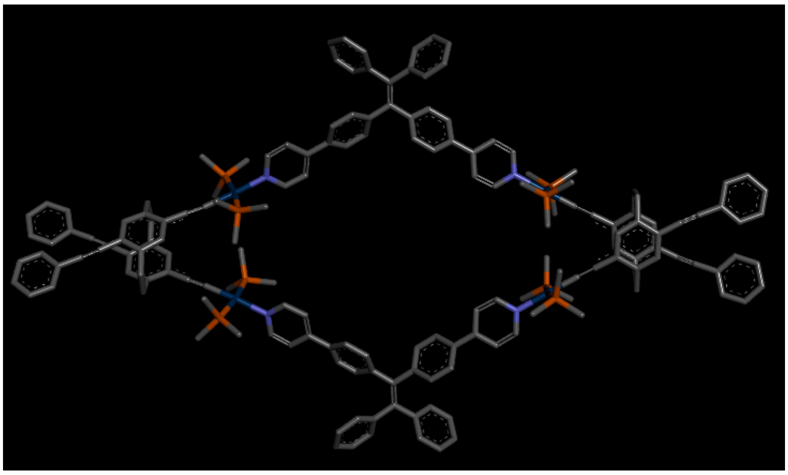

Supplementary Fig 14. Gaussian 16 optimized structure of the double helicate *R*p,*R*p-PCP-Tpy1

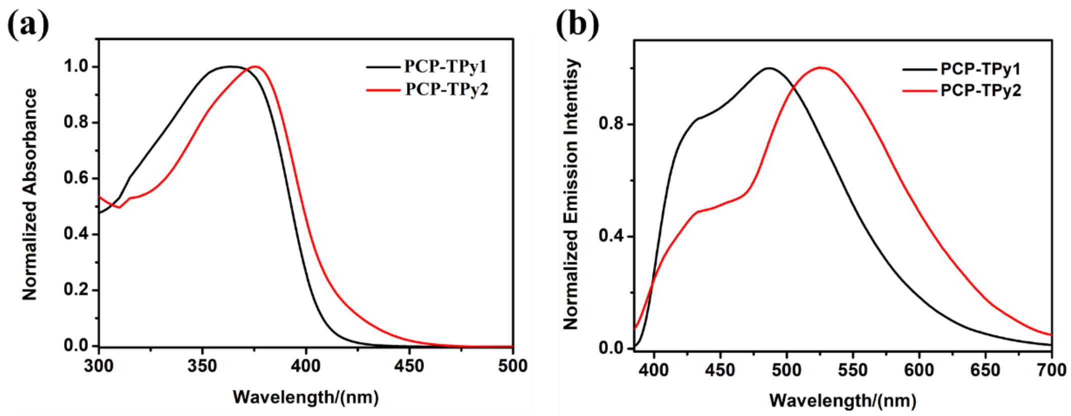

Supplementary Fig 15. UV absorption spectra and fluorescence emission spectra. (a) UV absorption spectra of **PCP-TPy1** (1×10^{-5}M) and **PCP-TPy2** (1×10^{-5}M) in the THF solvent, (b) Fluorescence emission spectra of **PCP-TPy1** (1×10^{-5}M) and **PCP-TPy2** (1×10^{-5}M) in the THF solvent (λ_{ex}=360 nm).

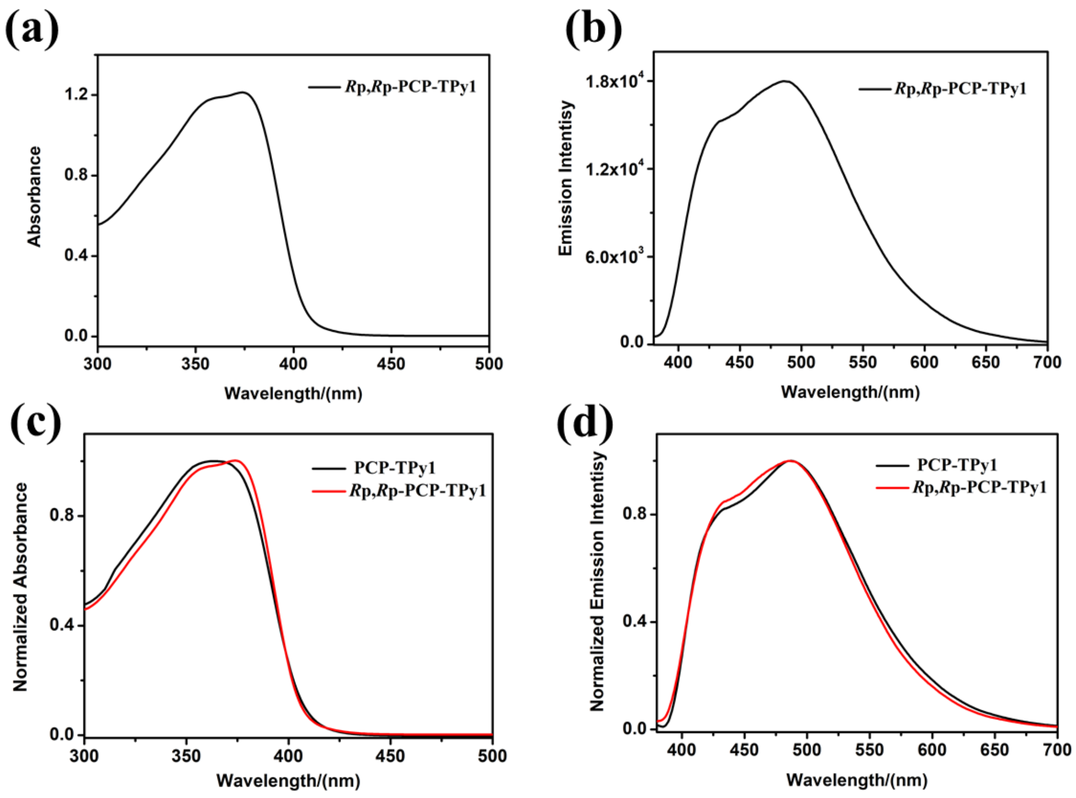

Supplementary Fig 16. UV absorption spectra and fluorescence emission spectra. UV absorption spectra (a) and fluorescence emission spectra (b) of ***R*p,*R*p-PCP-TPy1** (1×10^{-5}M) in the THF solvent (λ_{ex}=360 nm), UV absorption spectra (c) and fluorescence emission spectra (d) of **PCP-TPy1** (1×10^{-5}M) and ***R*p,*R*p-PCP-TPy1** (1×10^{-5}M) in the THF solvent (λ_{ex}=360 nm).

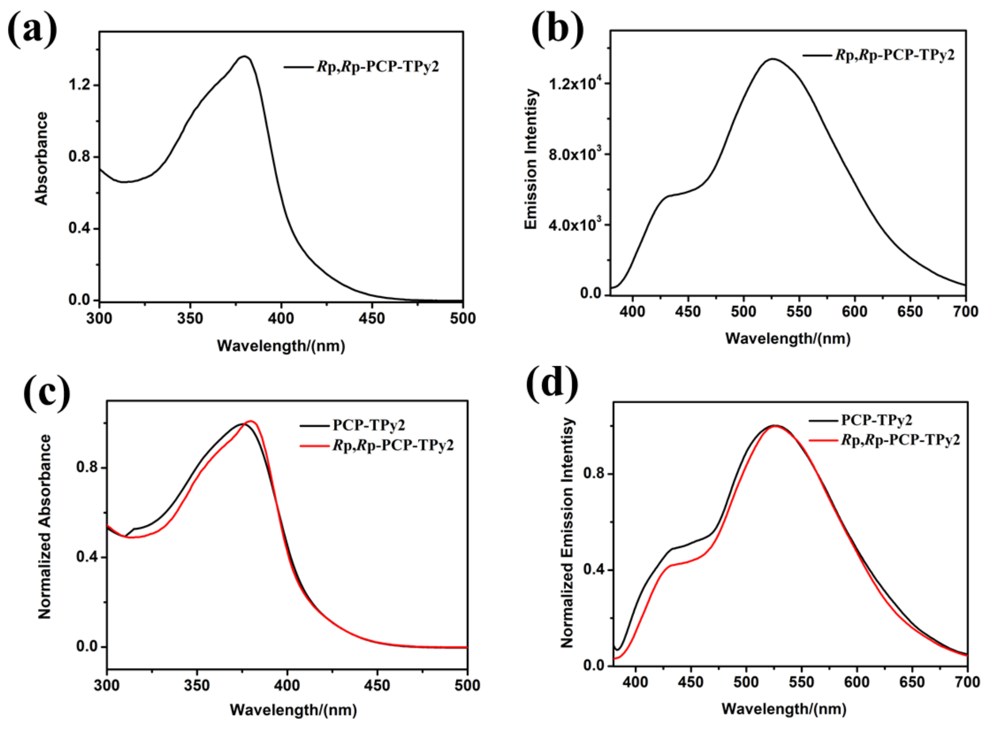

Supplementary Fig 17. UV absorption spectra and fluorescence emission spectra. UV absorption spectra (a) and fluorescence emission spectra (b) of ***R*p,*R*p-PCP-TPy2** (1×10^{-5}M) in the THF solvent (λ_{ex}=360 nm), UV absorption spectra (c) and fluorescence emission spectra (d) of **PCP-TPy2** (1×10^{-5}M) and ***R*p,*R*p-PCP-TPy2** (1×10^{-5}M) in the THF solvent (λ_{ex}=360 nm).

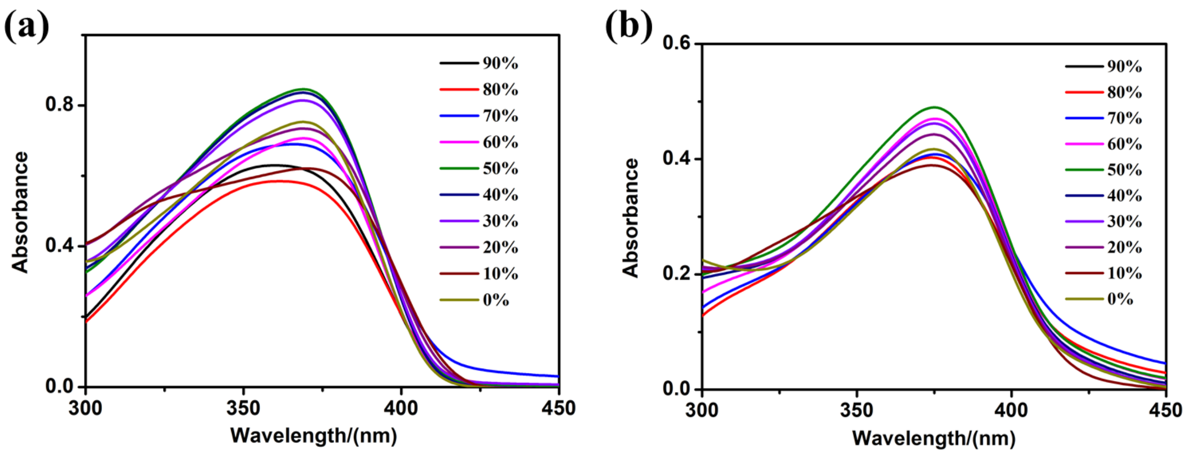

Supplementary Fig 18. UV absorption spectra. UV absorption spectra of (a) **PCP-TPy1** (0.5×10^{-5}M), (b) **PCP-TPy2** (0.5×10^{-5}M) with the different water fraction in the THF solvent

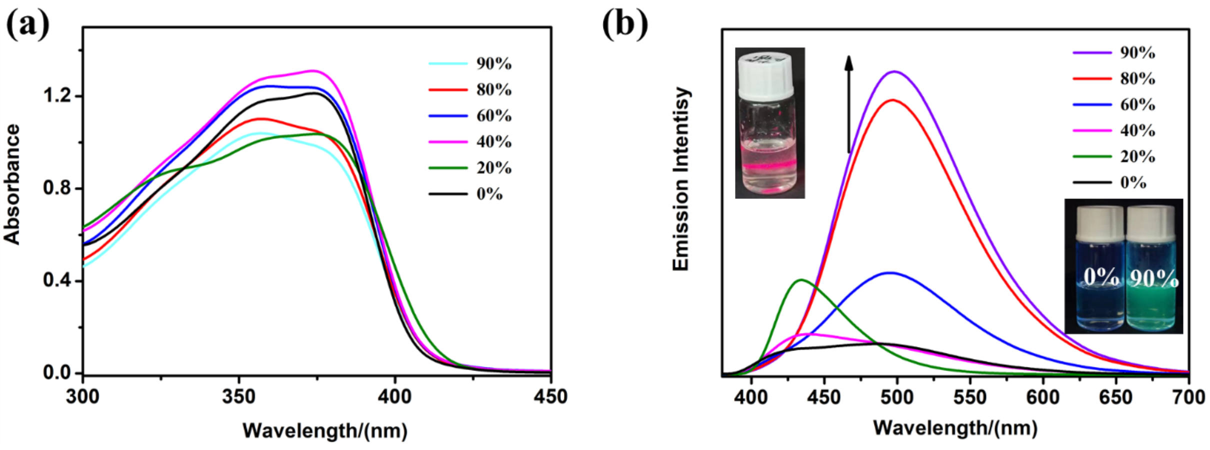

Supplementary Fig. 19 The AIE properties of novel double helicate. UV absorption spectra of (a) ***R*p,*R*p-PCP-TPy1** (1×10^{-5}M) with the different water fraction in the THF solvent, Fluorescence emission spectra (b) of ***R*p,*R*p-PCP-TPy1** (1×10^{-5}M) with different water fraction in THF solvent (λ_{ex} =360 nm). The inserted pictures in b is the Tyndall phenomena of ***R*p,*R*p-PCP-TPy1**. All concentrations are 1×10^{-5}M.

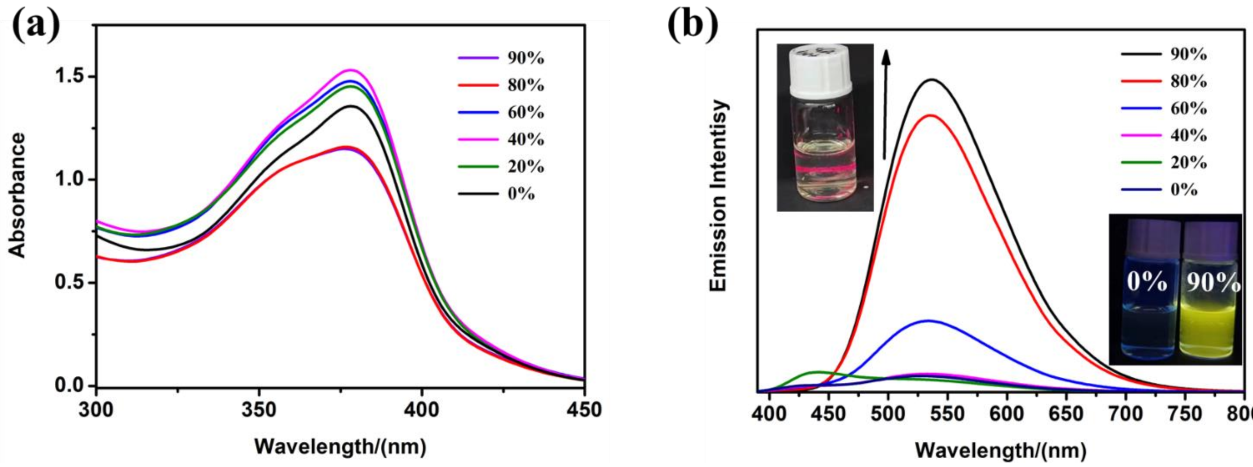

Supplementary Fig 20. The AIE properties of novel double helicate. UV absorption spectra of (a) ***R*p,*R*p-PCP-TPy2** (1×10^{-5}M) with the different water fraction in the THF solvent, Fluorescence emission spectra (b) of ***R*p,*R*p-PCP-TPy2** (1×10^{-5}M) with different water fraction in THF solvent (λ_{ex} =360 nm). The inserted pictures in b is the Tyndall phenomena of ***R*p,*R*p-PCP-TPy2**. All concentrations are 1×10^{-5}M.

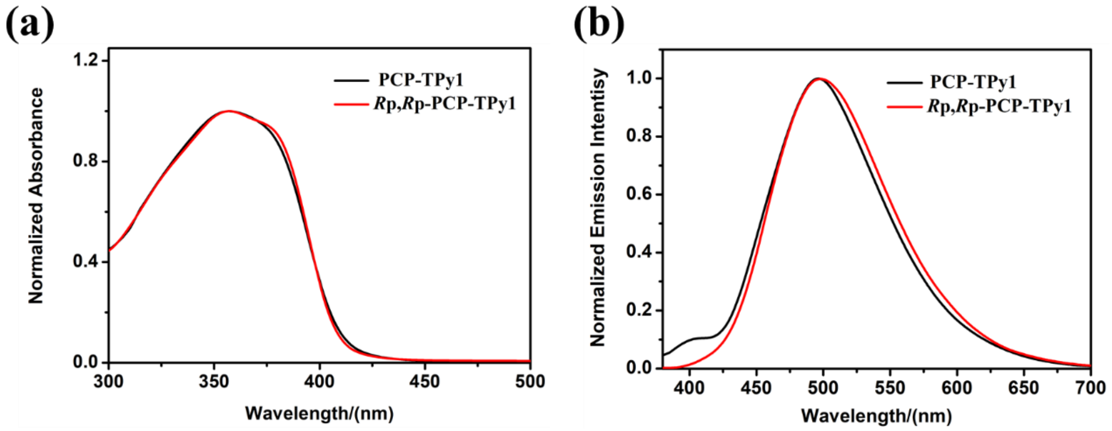

Supplementary Fig 21. UV absorption spectra and fluorescence emission spectra. UV absorption spectra (a) and fluorescence emission spectra (b) of **PCP-TPy1** (1×10^{-5}M) and ***R*p,*R*p-PCP-TPy1** (1×10^{-5}M) in the THF/Water (1:9, v/v) solvent (λ_{ex}=360 nm).

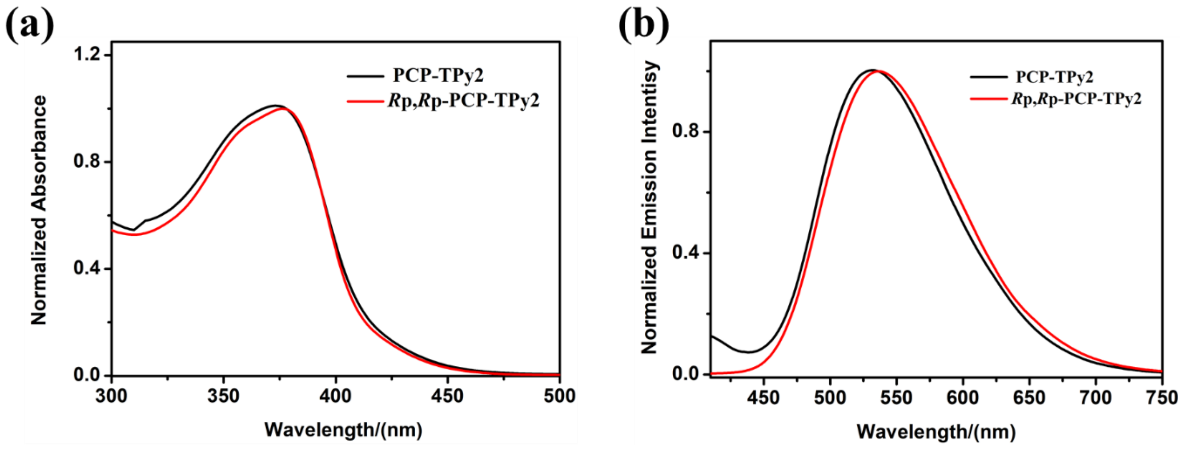

Supplementary Fig 22. UV absorption spectra and fluorescence emission spectra. UV absorption spectra (a) and fluorescence emission spectra (b) of **PCP-TPy2** (1×10^{-5}M) and ***R*p,*R*p-PCP-TPy2** (1×10^{-5}M) in the THF/Water (1:9, v/v) solvent (λ_{ex}=360 nm).

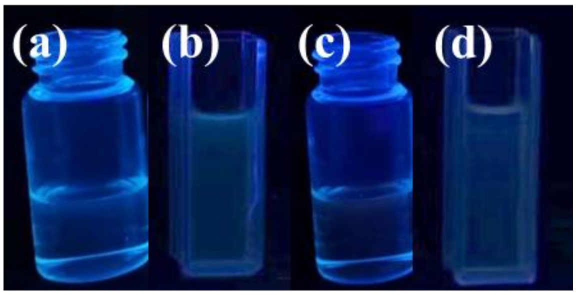

Supplementary Fig 23. Fluorescence photos in glass bottles or cuvette. Fluorescence photos of (a) **PCP-TPy1** (1×10^{-5}M), (c) **PCP-TPy2** (1×10^{-5}M) in glass bottles, Fluorescence photos of (b) **PCP-TPy1** (1×10^{-5}M), (d) **PCP-TPy2** (1×10^{-5}M) in cuvette.

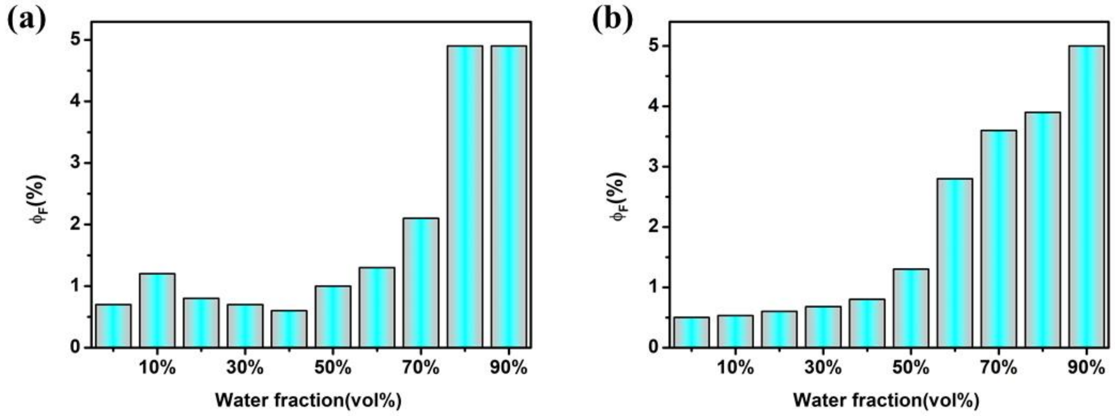

Supplementary Fig 24. UV absorption spectra and fluorescence emission spectra. Fluorescence quantum yields of (a) PCP-TPy1 (1×10^{-5}M) and (b) PCP-TPy2 (1×10^{-5}M) with different water contents in THF solvents (λ_{ex}=360 nm)

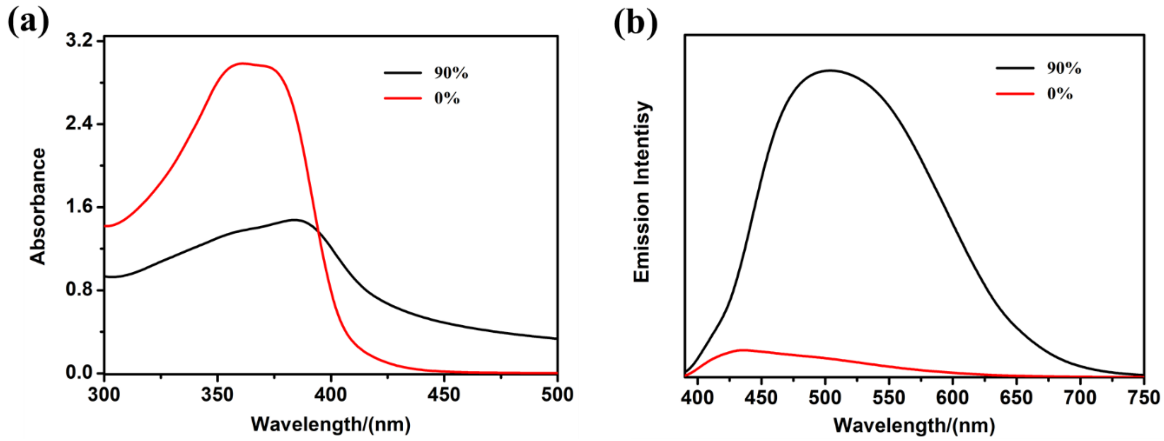

Supplementary Fig 25. UV absorption spectra and fluorescence emission spectra. UV absorption spectra of (a) **PCP-TPy1** (1×10^{-5}M) with the different Hexane fraction in the DCM solvent, Fluorescence emission spectra (b) of **PCP-TPy1** (1×10^{-5}M) with the different Hexane fraction in the DCM solvent (λ_{ex} =360 nm).

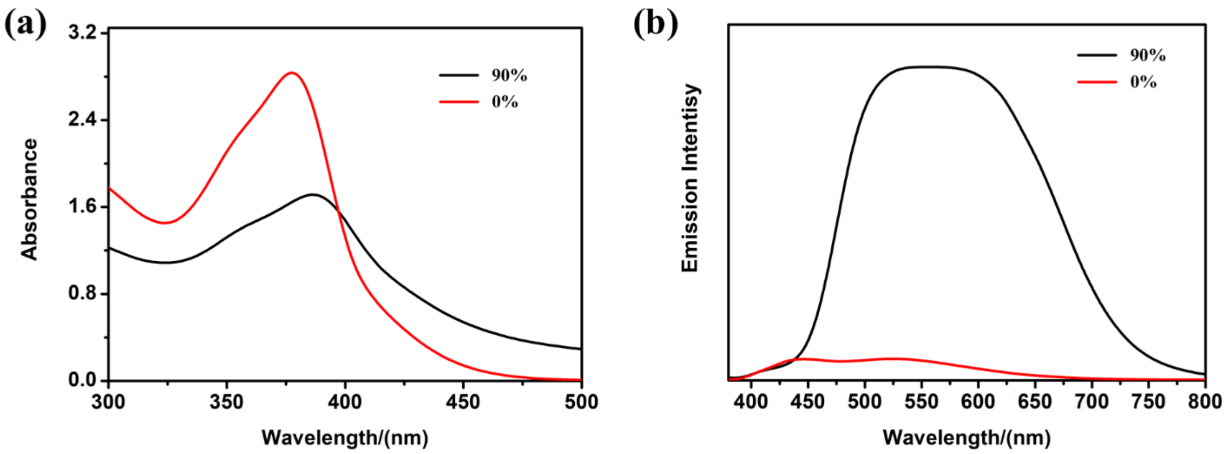

Supplementary Fig 26. UV absorption spectra and fluorescence emission spectra. UV absorption spectra of (a) **PCP-TPy2** (1×10^{-5}M) with the different Hexane fraction in the DCM solvent; Fluorescence emission spectra (b) of **PCP-TPy2** (1×10^{-5}M) with the different Hexane fraction in the DCM solvent (λ_{ex} =360 nm).

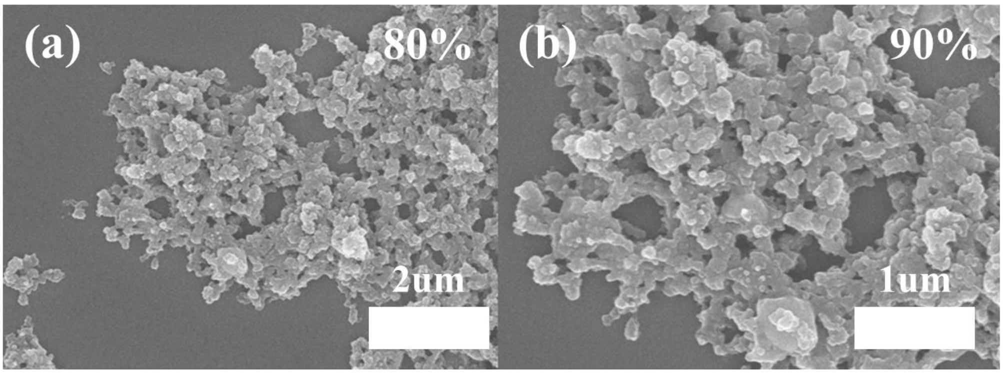

Supplementary Fig 27. The SEM image of PCP-TPy2 (a) in the mixture of THF/water (2:8, v/v). The SEM image of PCP-TPy2 (b) in the mixture of THF/water (1:9, v/v)

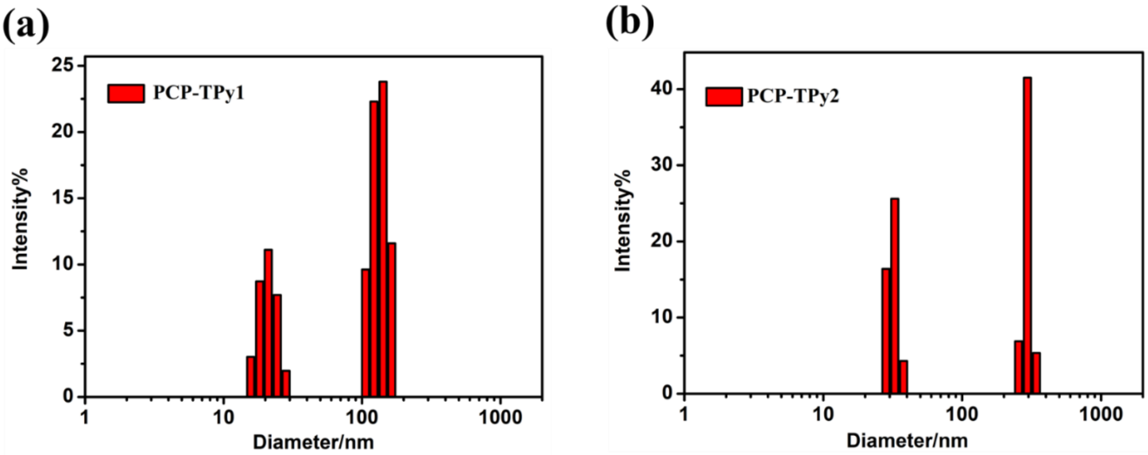

Supplementary Fig 28. DLS experiment (a) DLS profiles of the double helicate of **PCP-TPy1** (1×10-5M) in the mixture of THF/water (1:9, v/v), (b) DLS profiles of the double helicate of **PCP-TPy2** (1×10-5M) in the mixture of THF/water (1:9, v/v).

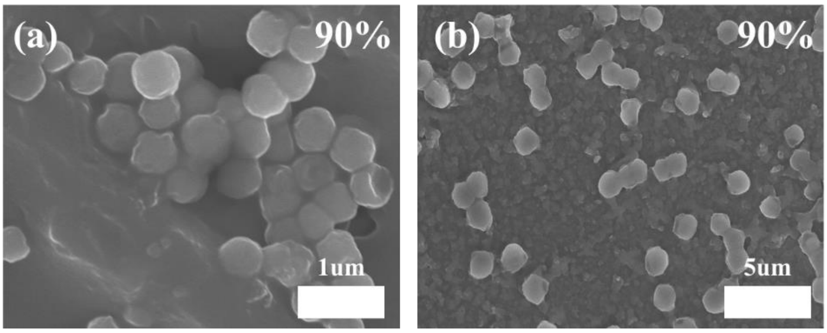

Supplementary Fig 29. The SEM images of *R*p,*R*p-PCP-TPy1 (a),(b) in the mixture of THF/water (1:9, v/v).

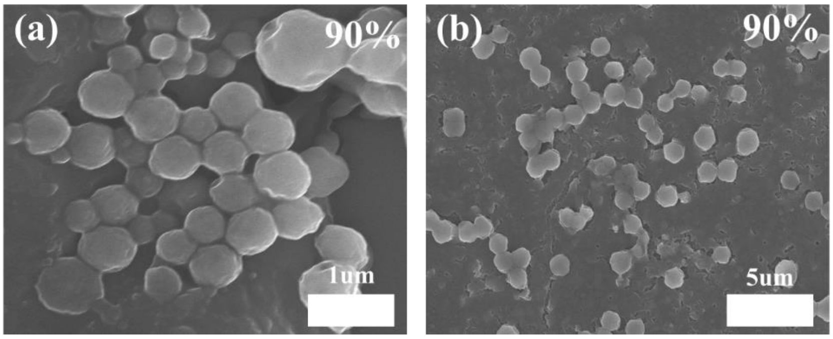

Supplementary Fig 30. The SEM images of *R*p,*R*p-PCP-Tpy2 (a),(b) in the mixture of THF/water (1:9, v/v).

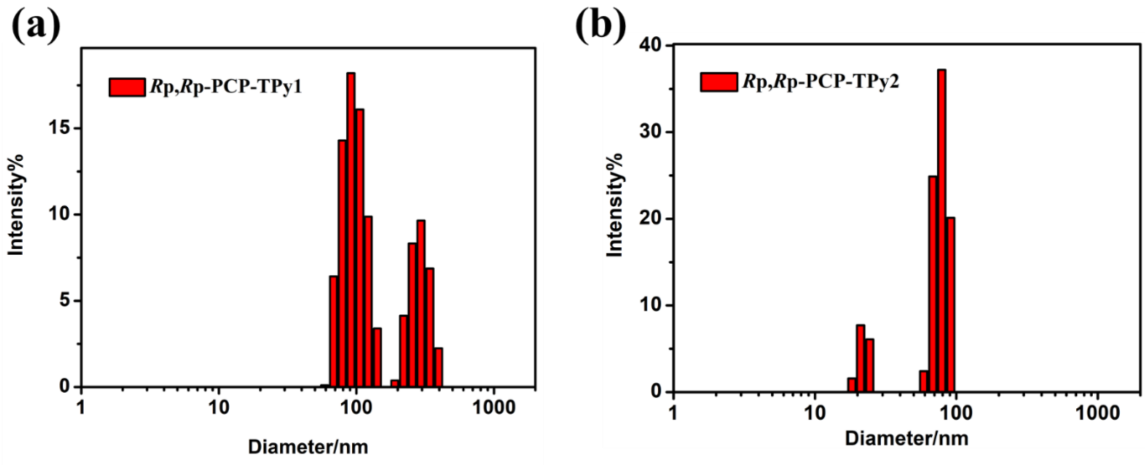

Supplementary Fig 31. DLS experiment (a) DLS profiles of the double helicate of ***R*p,*R*p-PCP-TPy1** (1×10^{-5}M) in the mixture of THF/water (1:9, v/v), (b) DLS profiles of the double helicate of ***R*p,*R*p-PCP-TPy2** (1×10^{-5}M) in the mixture of THF/water (1:9, v/v).

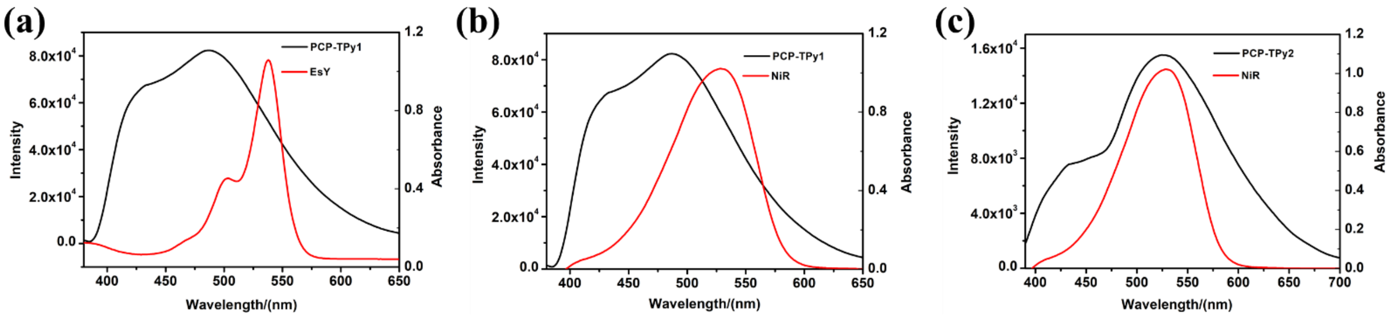

Supplementary Fig 32. UV absorption spectra and fluorescence emission spectra. (a) The absorption spectra of EsY (1×10^{-7}M) and fluorescence emission spectrum of **PCP-TPy1** (1×10^{-5}M) (λ_{ex} = 360 nm) in THF solvent, (b) The absorption spectra of NiR (1×10^{-7}M) and fluorescence emission spectra of **PCP-TPy1** (1×10^{-5}M) (λ_{ex} = 360 nm) in THF solvent, (c) The absorption spectra of NiR (10^{-7}M) and fluorescence emission spectra of **PCP-TPy2** (1×10^{-5}M) (λ_{ex} = 360 nm) in THF solvent.

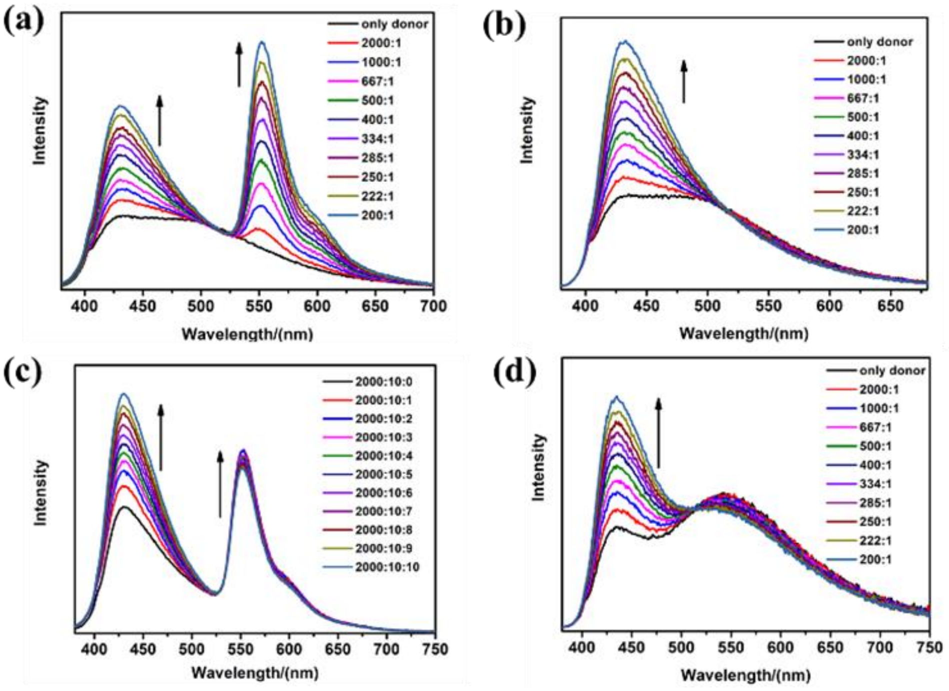

Supplementary Fig 33. Fluorescence emission spectra. Fluorescence emission spectra (a) (b) of **PCP-TPy1** (1×10^{-5}M) with different concentrations of EsY(1×10^{-7}M), NiR (1×10^{-7}M) in THF solvent (λ_{ex} = 360 nm, slit widths: ex = 5 nm, em = 5 nm), Fluorescence emission spectra (c) of **PCP-TPy1/EsY** (1×10^{-5}M) with different concentrations of NiR(1×10^{-7}M) in THF solvent (λ_{ex} = 360 nm, slit widths: ex = 5 nm, em = 5 nm), Fluorescence emission spectra (d) of **PCP-Tpy2** (1×10^{-5}M) with different concentrations of NiR(1×10^{-7}M) in THF solvent (λ_{ex} = 360 nm, slit widths: ex = 5 nm, em = 5 nm).

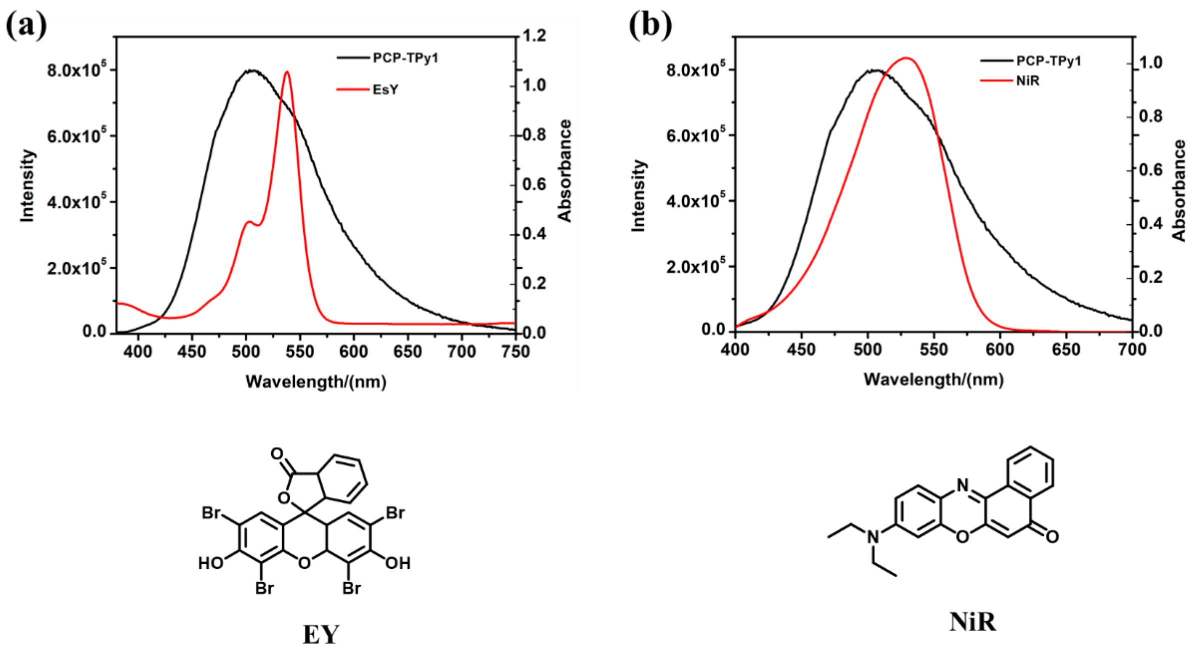

Supplementary Fig 34. UV absorption spectra and fluorescence emission spectra. (a) The absorption spectra of EsY (1×10^{-7}M) and fluorescence emission spectrum of **PCP-TPy1** (1×10^{-5}M) (λex = 360 nm) in THF/Water (1:9, v/v) solvent; (b) The absorption spectra of NiR (1×10^{-7}M) and fluorescence emission spectra of **PCP-TPy1** (1×10^{-5}M) (λ_{ex} = 360 nm) in THF/Water (1:9, v/v) solvent.

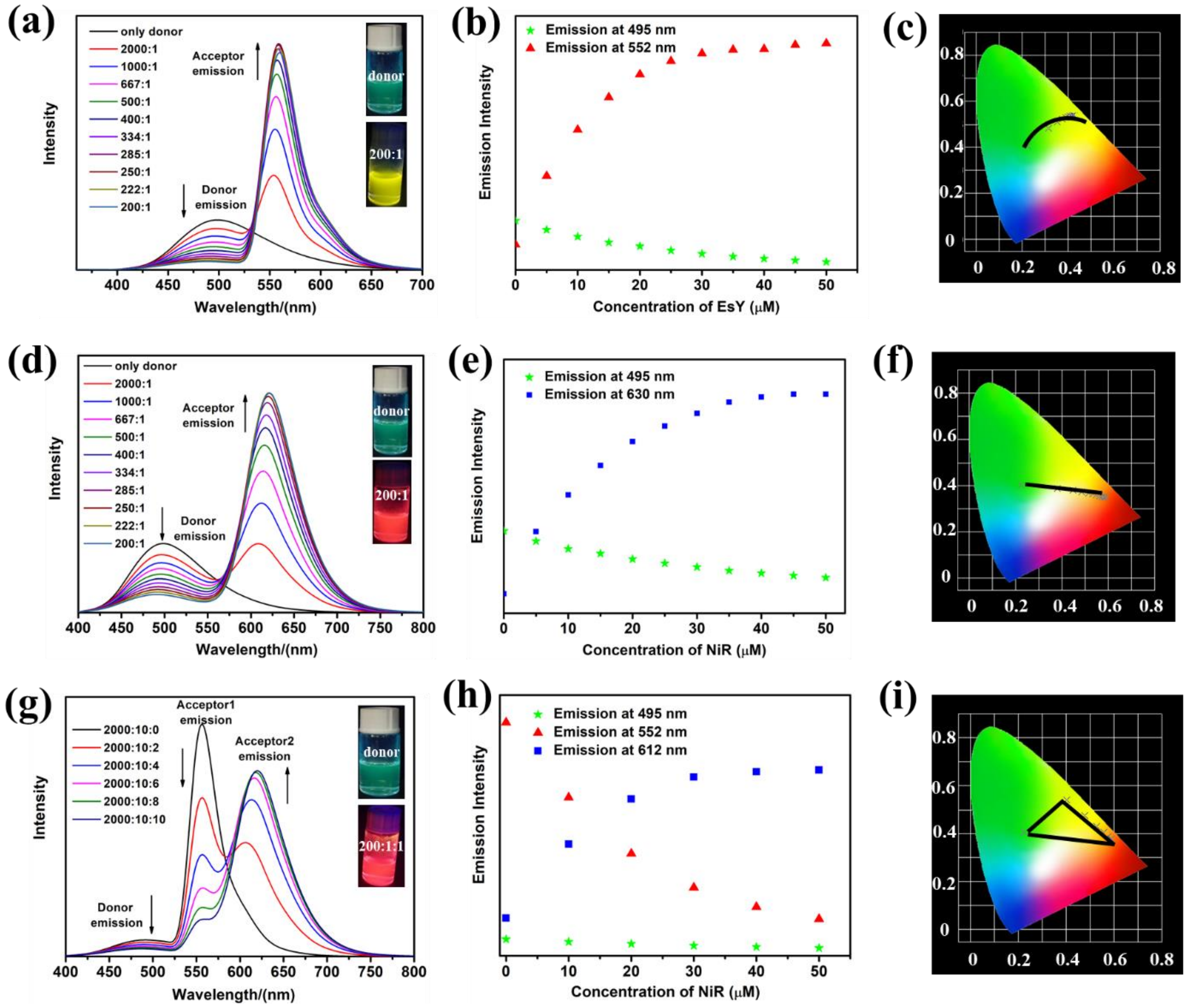

Supplementary Fig 35. ALHSs based on *R*p,*R*p-PCP-TPy1. Fluorescence emission spectra (a) of ***R*p,*R*p-PCP-TPy1** (1×10^{-5}M) with different concentrations of EsY(1×10^{-7}M) (λ_{ex} = 360 nm, slit widths: ex = 2.5 nm, em = 2.5 nm) in the mixture of THF/water (1:9, v/v), Fluorescent intensity changes (b) at 495 and 552 nm. Fluorescence emission of ***R*p,*R*p-PCP-TPy1/EsY** in the CIE (c), Fluorescence emission spectra (d) of ***R*p,*R*p-PCP-TPy1** (1×10^{-5}M) with different concentrations of NiR(1×10^{-7}M) (λ_{ex} = 360 nm, slit widths: ex = 2.5 nm, em = 2.5 nm) in the mixture of THF/water (1:9, v/v), Fluorescent intensity changes (e) at 495 and 630 nm. Fluorescence emission of ***R*p,*R*p-PCP-TPy1/NiR** in the CIE (f), Fluorescence emission spectra (g) of ***R*p,*R*p-PCP-TPy1/EsY** (1×10^{-5}M) with different concentrations of NiR (1×10^{-7}M) (λ_{ex} = 360 nm, slit widths: ex = 2.5 nm, em = 2.5 nm) in the mixture of THF/water (1:9, v/v), Fluorescent intensity changes (h) at 495, 552 and 612 nm.Fluorescence emission of ***R*p,*R*p-PCP-TPy1/EsY/NiR** in the CIE (i).

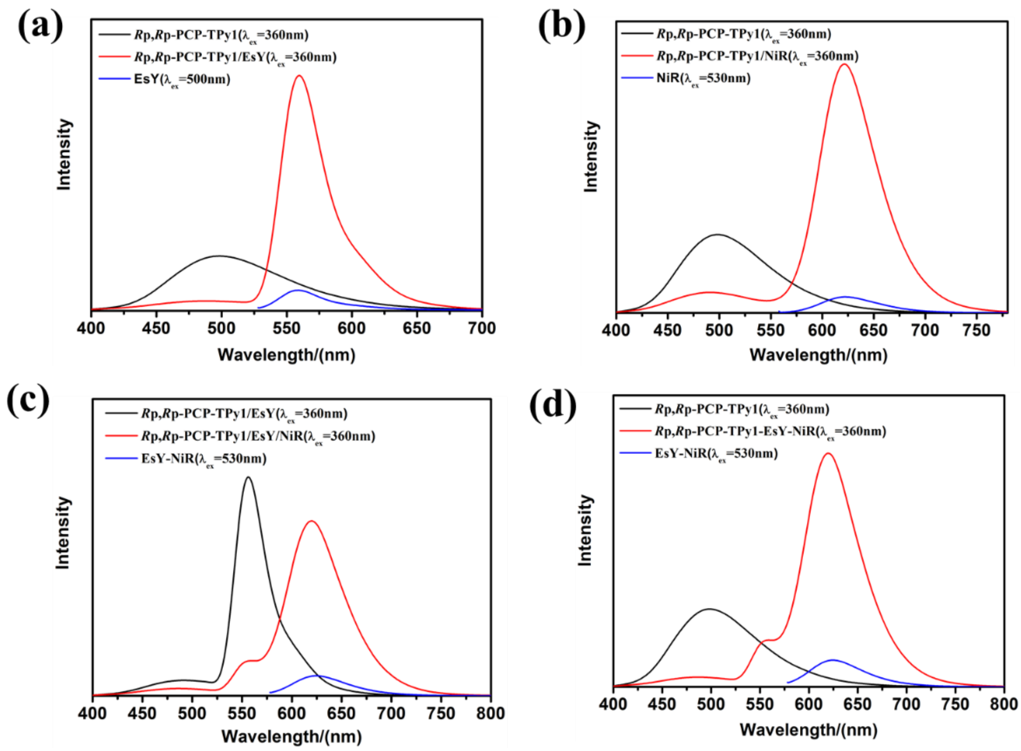

Supplementary Fig 36. energy transfer efficiency and antenna effect. (a) Fluorescence spectra of ***R*p,*R*p-PCP-TPy1/EsY** in THF/water, blue line (acceptor emission, λ_{ex} = 500 nm) (***R*p,*R*p-PCP-TPy1** = 1×10^{-5} M, EsY = 1×10^{-7} M, slit widths: ex = 2.5 nm, em = 2.5 nm), the black line represents the fluorescence spectrum of ***R*p,*R*p-PCP-TPy1**, which was normalized according to the fluorescence intensity at 552 nm of the red line, (b) Fluorescence spectra of ***R*p,*R*p-PCP-TPy1** in THF/water, blue line (acceptor emission, λ_{ex} = 530 nm) (***R*p,*R*p-PCP-TPy1** = 1×10^{-5} M, NiR = 1×10^{-7} M, slit widths: ex = 2.5 nm, em = 2.5 nm), the black line represents the fluorescence spectrum of ***R*p,*R*p-PCP-TPy1**, which was normalized according to the fluorescence intensity at 630 nm of the red line (c) Fluorescence spectra of ***R*p,*R*p-PCP-TPy1-EsY/NiR** in THF/Water, blue line (acceptor emission, λ_{ex} = 530 nm) (***R*p,*R*p-PCP-TPy1/EsY** = 1×10^{-5} M, NiR = 1×10^{-7} M, slit widths: ex = 2.5 nm, em = 2.5 nm), the black line represents the fluorescence spectrum of ***R*p,*R*p-PCP-TPy1/EsY**, which was normalized according to the fluorescence intensity at 630 nm of the red line (d) Fluorescence spectra of ***R*p,*R*p-PCP-TPy1/EsY/NiR** in THF/Water, blue line (acceptor emission, λ_{ex} = 530 nm) (***R*p,*R*p-PCP-TPy1/EsY** = 1×10^{-5} M, NiR = 1×10^{-7} M, slit widths: ex =2.5 nm, em = 2.5 nm), the black line represents the fluorescence spectrum of ***R*p,*R*p-PCP-TPy1**, which was normalized according to the fluorescence intensity at 630 nm of the red line.

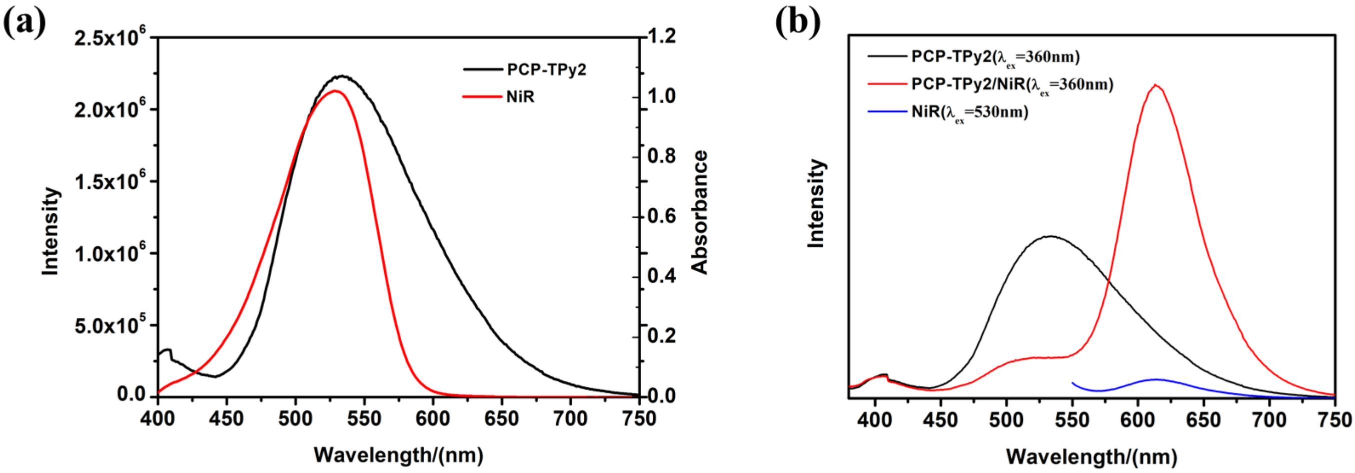

Supplementary Fig 37. UV absorption spectra and fluorescence emission spectra. (a) The absorption spectrum of NiR (1×10^{-7}M) and fluorescence emission spectrum of **PCP-TPy2** (1×10^{-5}M) (λ_{ex} = 360 nm) in THF/Water (1:9, v/v) solvent, (b) Fluorescence spectra of **PCP-TPy2/NiR** in THF/water, blue line (acceptor emission, λ_{ex} = 530 nm, slit widths: ex = 5 nm, em = 5 nm) (**PCP-TPy2** = 1×10^{-5} M, NiR = 1×10^{-7} M), the black line represents the fluorescence spectrum of **PCP-TPy2**, which was normalized according to the fluorescence intensity at 630 nm of the red line.

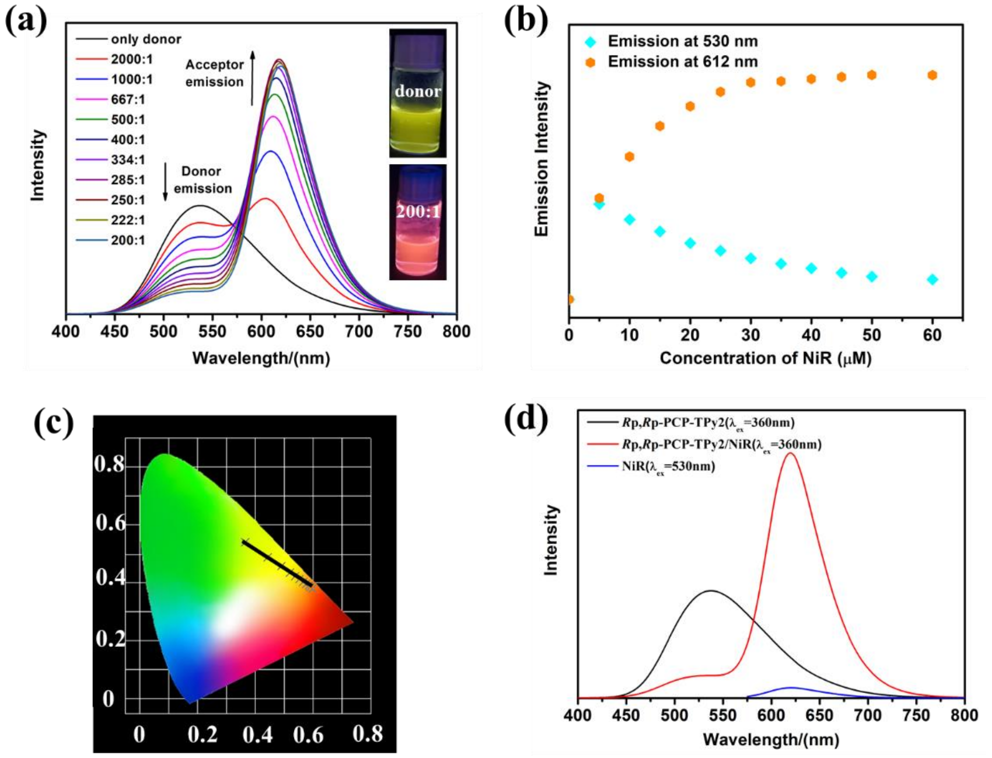

Supplementary Fig. 38 ALHSs based on *R*p,*R*p-PCP-TPy1. Fluorescence emission spectra (a) of ***R*p,*R*p-PCP-TPy2** (1×10^{-5}M) with different concentrations of NiR(1×10^{-7}M) (λex = 360 nm, slit widths: ex = 2.5 nm, em = 2.5 nm) in the mixture of THF/water (1:9, v/v), Fluorescent intensity changes (b) at 530 and 612 nm. Fluorescence emission of ***R*p,*R*p-PCP-TPy2/NiR** in the CIE (c), Fluorescence spectra (d) of ***R*p,*R*p-PCP-TPy2/NiR** in THF/water, blue line (acceptor emission, λ_{ex} = 530 nm) (***R*p,*R*p-PCP-TPy2** = 1×10^{-5} M, NiR = 1×10^{-7} M, slit widths: ex =2.5 nm, em = 2.5 nm), the black line represents the fluorescence spectrum of ***R*p,*R*p-PCP-TPy2**, which was normalized according to the fluorescence intensity at 630 nm of the red line.

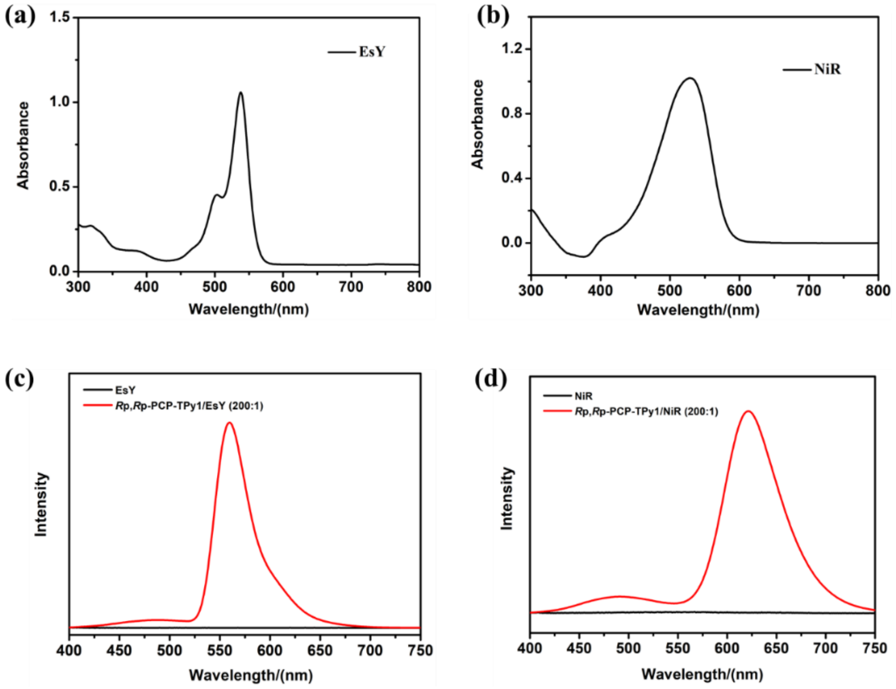

Supplementary Fig 39. UV absorption spectra and fluorescence emission spectra. (a) The absorption spectra of EsY (1×10^{-5}M), (b) The absorption spectra of NiR (1×10^{-5}M), (c) Fluorescence emission spectra of EsY (1×10^{-7}M) and ***R*p,*R*p-PCP-TPy1**(1×10^{-5}M)/**EsY** (1×10^{-7}M) (200:1), (d) Fluorescence emission spectra of NiR (1×10^{-7}M) and ***R*p,*R*p-PCP-TPy1**(1×10^{-5}M)/**NiR** (1×10^{-7}M) (200:1), All experiments were performed in the mixture of THF/water (1:9, v/v), λ_{ex} = 360 nm, slit widths: ex = 2.5 nm, em = 2.5 nm.

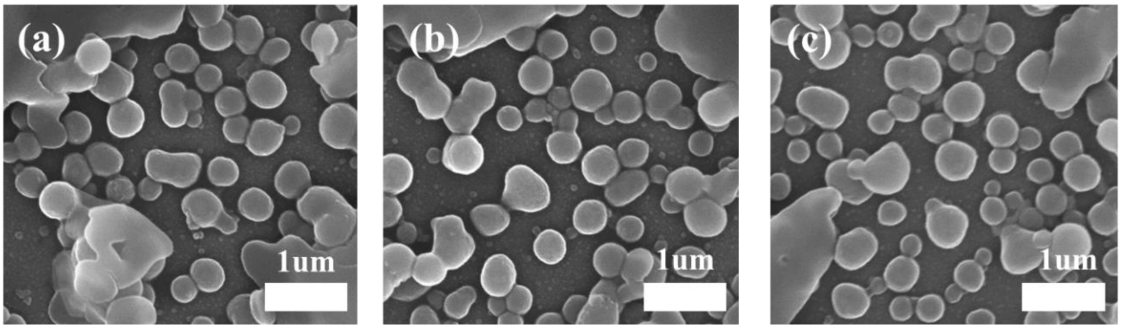

Supplementary Fig 40. SEM image (a) SEM image of the **PCP-TPy1**(1×10^{-5}M)/**EsY** (1×10^{-7}M) (200:1) in the mixture of THF/water (1:9, v/v), (b) SEM image of the **PCP-TPy1**(1×10^{-5}M)/**NiR** (1×10^{-7}M) (200:1) in the mixture of THF/water (1:9, v/v), (c) SEM image of the **PCP-TPy1**(1×10^{-5}M)/**EsY/NiR** (1×10^{-7}M) (2000:10:10) in the mixture of THF/water (1:9, v/v), (d) SEM image of the **PCP-TPy2**(1×10^{-5}M)/**NiR** (1×10^{-7}M) (200:1) in the mixture of THF/water (1:9, v/v).

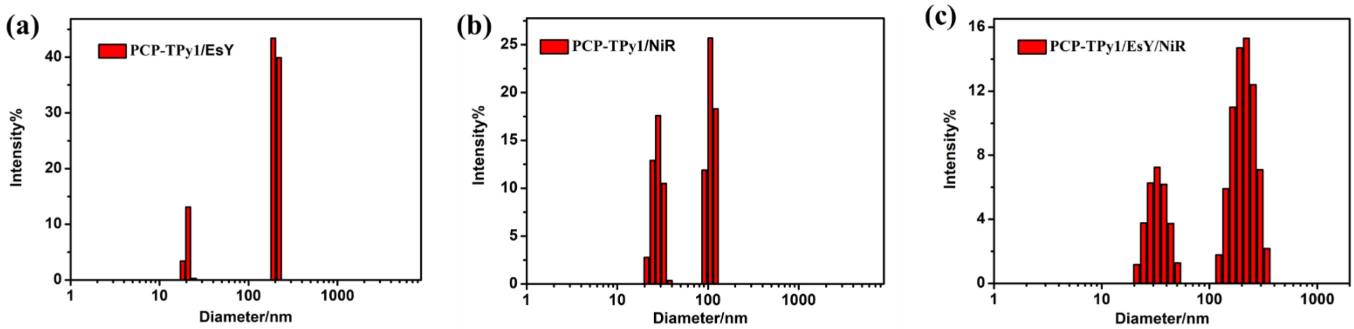

Supplementary Fig 41. DLS experiment (a) DLS profiles of the system of **PCP-TPy1**(1×10^{-5}M)/**EsY** (1×10^{-7}M) (200:1) in the mixture of THF/water (1:9, v/v), (b) DLS profiles of the system of **PCP-TPy1**(1×10^{-5}M)/**NiR** (1×10^{-7}M) (200:1) in the mixture of THF/water (1:9, v/v), (c) DLS profiles of the system of **PCP-TPy1**(1×10^{-5}M)/**EsY/NiR** (1×10^{-7}M) (2000:10:10) in the mixture of THF/water (1:9, v/v).

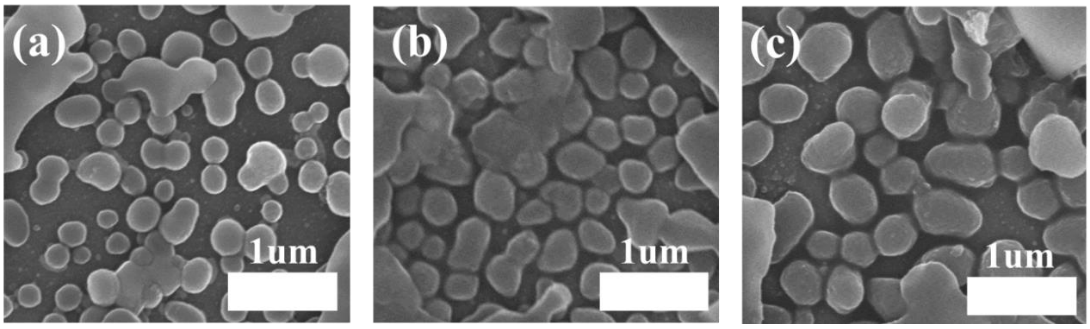

Supplementary Fig 42. SEM image (a) SEM image of the ***R*p,*R*p-PCP-TPy1**(1×10^{-5}M)/**EsY** (1×10^{-7}M) (200:1) in the mixture of THF/water (1:9, v/v), (b) SEM image of the ***R*p,*R*p-PCP-TPy1**(1×10^{-5}M)/**NiR** (1×10^{-7}M) (200:1) in the mixture of THF/water (1:9, v/v), (c) SEM image of the ***R*p,*R*p-PCP-TPy1**(1×10^{-5}M)/**EsY/NiR** (1×10^{-7}M) (2000:10:10) in the mixture of THF/water (1:9, v/v), (d) SEM image of the ***R*p,*R*p-PCP-TPy2**(1×10^{-5}M)/**NiR** (1×10^{-7}M) (200:1) in the mixture of THF/water (1:9, v/v).

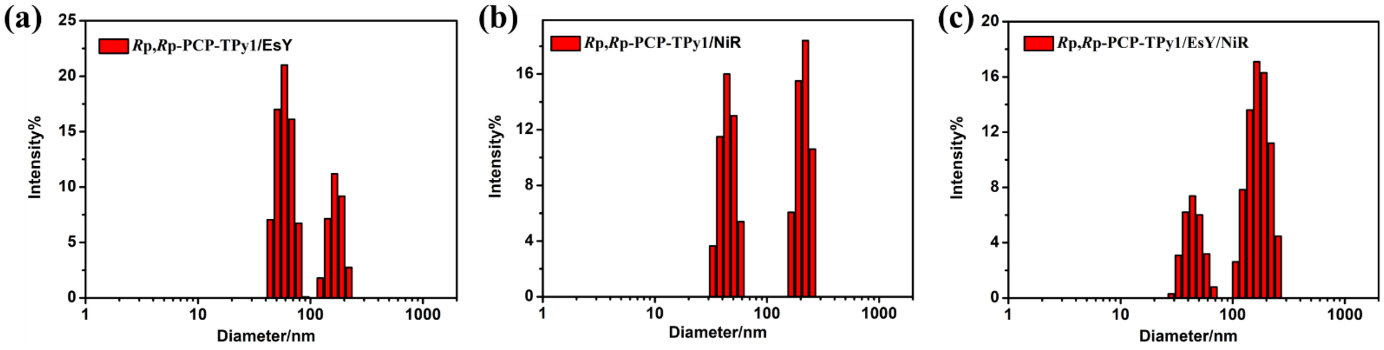

Supplementary Fig 43. DLS experiment (a) DLS profiles of the system of ***R*p,*R*p-PCP-TPy1**(1×10^{-5}M)/**EsY** (1×10^{-7}M) (200:1) in the mixture of THF/water (1:9, v/v), (b) DLS profiles of the system of ***R*p,*R*p-PCP-TPy1**(1×10^{-5}M)/**NiR** (1×10^{-7}M) (200:1) in the mixture of THF/water (1:9, v/v), (c) DLS profiles of the system of ***R*p,*R*p-PCP-TPy1**(1×10^{-5}M)/**EsY/NiR** (1×10^{-7}M) (2000:10:10) in the mixture of THF/water (1:9, v/v).

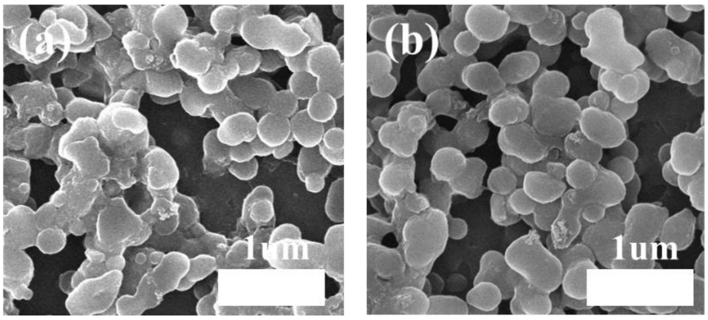

Supplementary Fig 44. SEM image (a) SEM image of the **PCP-TPy2** (1×10^{-5}M)/**NiR** (1×10^{-7}M) (200:1) in the mixture of THF/water (1:9, v/v), (b) SEM image of the ***R*p,*R*p-PCP-TPy1**(1×10^{-5}M)/**NiR** (1×10^{-7}M) (200:1) in the mixture of THF/water (1:9, v/v).

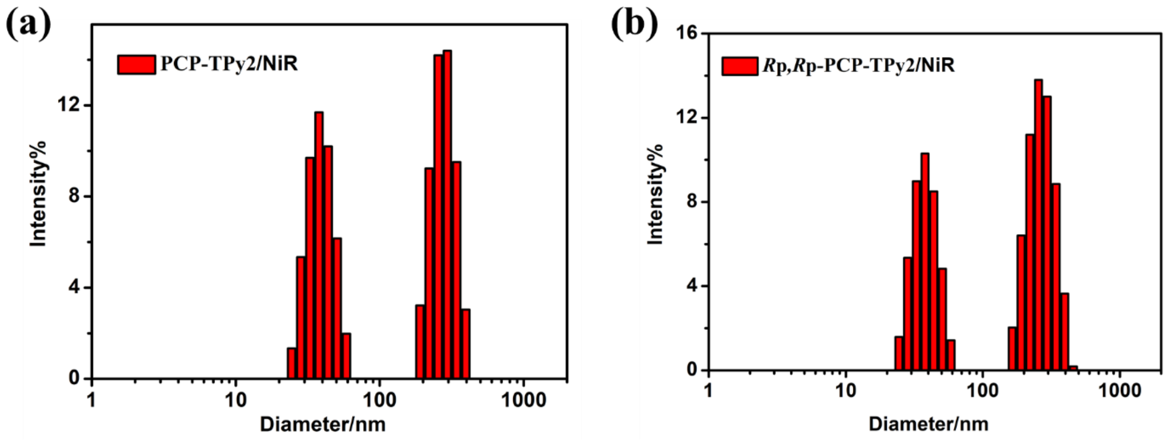

Supplementary Fig 45. DLS experiment (a) DLS profiles of the system of **PCP-TPy2**(1×10^{-5}M)/**NiR** (1×10^{-7}M) (200:1) in the mixture of THF/water (1:9, v/v), (b) DLS profiles of the system of ***R*p,*R*p-PCP-TPy2**(1×10^{-5}M)/**NiR** (1×10^{-7}M) (200:1) in the mixture of THF/water (1:9, v/v).

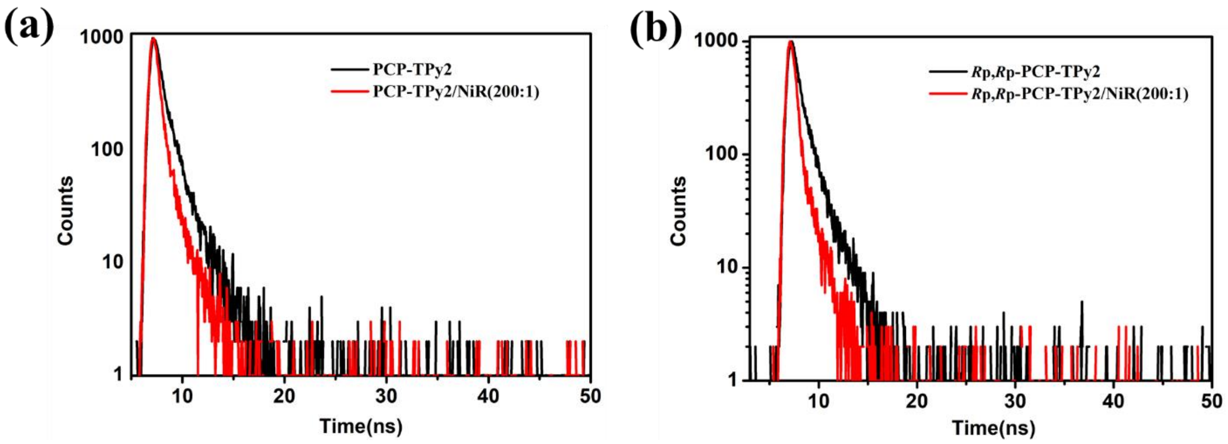

Supplementary Fig 46. Fluorescence decay experiments. (a) Change in the fluorescence decay profiles of the **PCP-TPy2, PCP-TPy2/NiR (200:1)** in THF/Water (1:9; v/v), (b) Change in the fluorescence decay profiles of the ***R*p-PCP-TPy2, *R*p-PCP-TPy2/NiR (200:1)** in THF/Water (1:9; v/v).

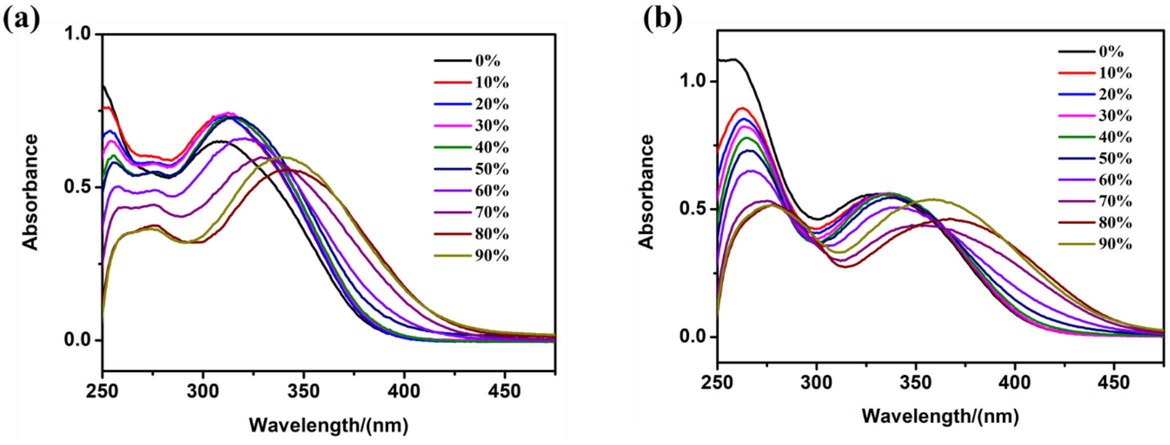

Supplementary Fig 47. UV absorption spectra. UV absorption spectra of (a) **TPy1** (1×10^{-5}M), (b) **TPy2** (1×10^{-5}M) with the different water fraction in the THF solvent.

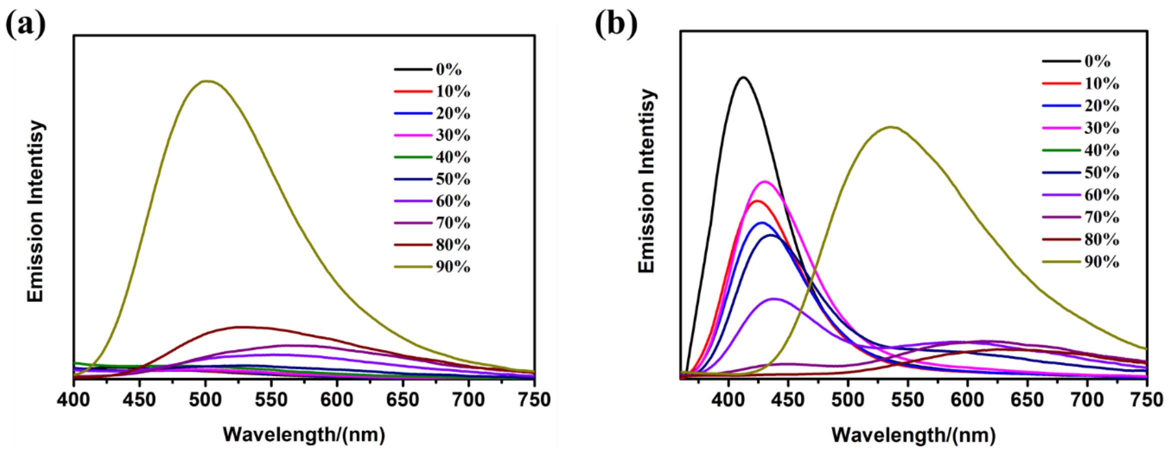

Supplementary Fig 48. Fluorescence emission spectra. Fluorescence emission spectra of (a) **TPy1** (1×10^{-5}M), (b) **TPy2** (1×10^{-5}M) with the different water fraction in the THF solvent (λ_{ex}=360 nm)

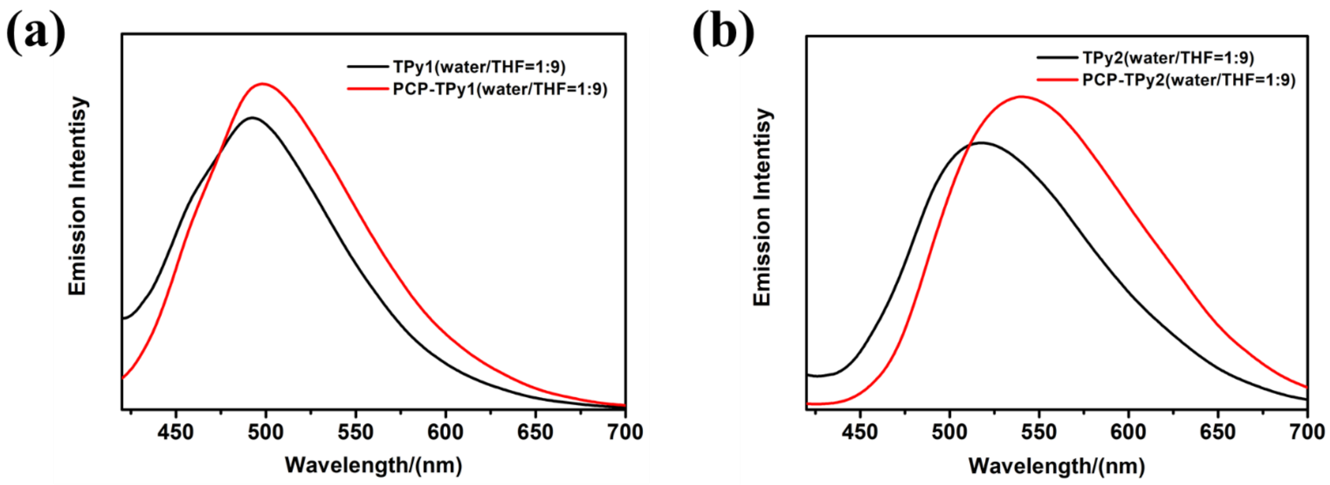

Supplementary Fig 49. Fluorescence emission spectra. Fluorescence emission spectra of (a) **TPy1** ($2×10^{-5}$M) and **PCP-TPy1** ($1×10^{-5}$M), (b) **TPy2** ($2×10^{-5}$M) and **PCP-TPy2** ($1×10^{-5}$M) in the THF/Water (1:9, v/v) solvent (λ_{ex}=360 nm).

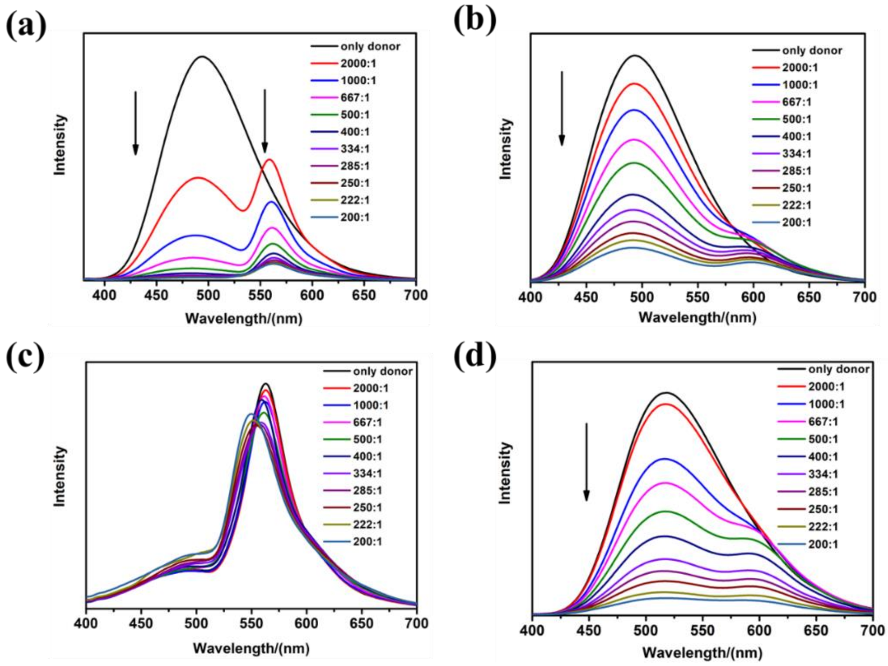

Supplementary Fig 50. Fluorescence emission spectra. Fluorescence emission spectra (a) (b) of **TPy1** ($1×10^{-5}$M) with different concentrations of EsY($1×10^{-7}$M),NiR ($1×10^{-7}$M) in the THF/Water (1:9, v/v) solvent (λ_{ex} = 360 nm, slit widths: ex = 2.5 nm, em = 2.5 nm), Fluorescence emission spectra (c) of **TPy1/EsY** ($1×10^{-5}$M) with different concentrations of NiR ($1×10^{-7}$M) in the THF/Water (1:9, v/v) solvent (λ_{ex} = 360 nm, slit widths: ex = 2.5 nm, em = 2.5 nm), Fluorescence emission spectra (d) of **Tpy2** ($1×10^{-5}$M) with different concentrations of NiR($1×10^{-7}$M) in the THF/Water (1:9, v/v) solvent (λ_{ex} = 360 nm, slit widths: ex = 2.5 nm, em = 2.5 nm).

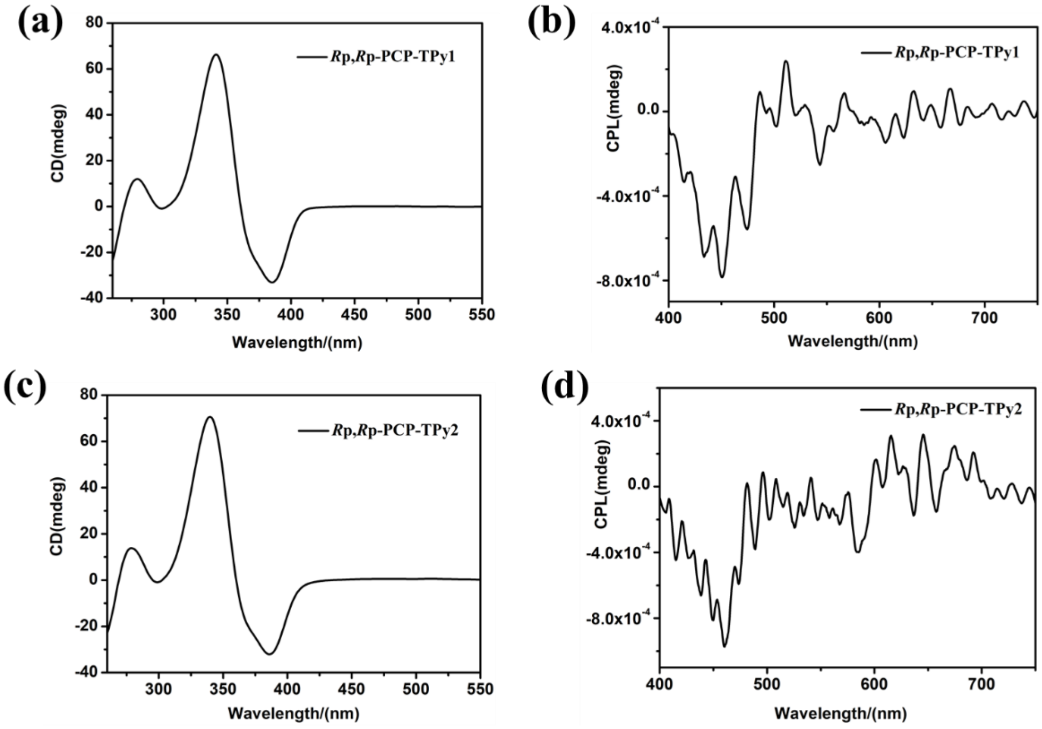

Supplementary Fig 51. CD spectra and CPL spectra. (a) The CD spectra of ***R*p,*R*p-PCP-TPy1** (1×10^{-5}M) in the THF solvent, (b) The CPL spectra of ***R*p,*R*p-PCP-TPy1** (1×10^{-5}M) in the THF solvent, (c) The CD spectra of ***R*p,*R*p-PCP-TPy2** (1×10^{-5}M) in the THF solvent, (d) The CPL spectra of ***R*p,*R*p-PCP-TPy2** (1×10^{-5}M) in the THF solvent.

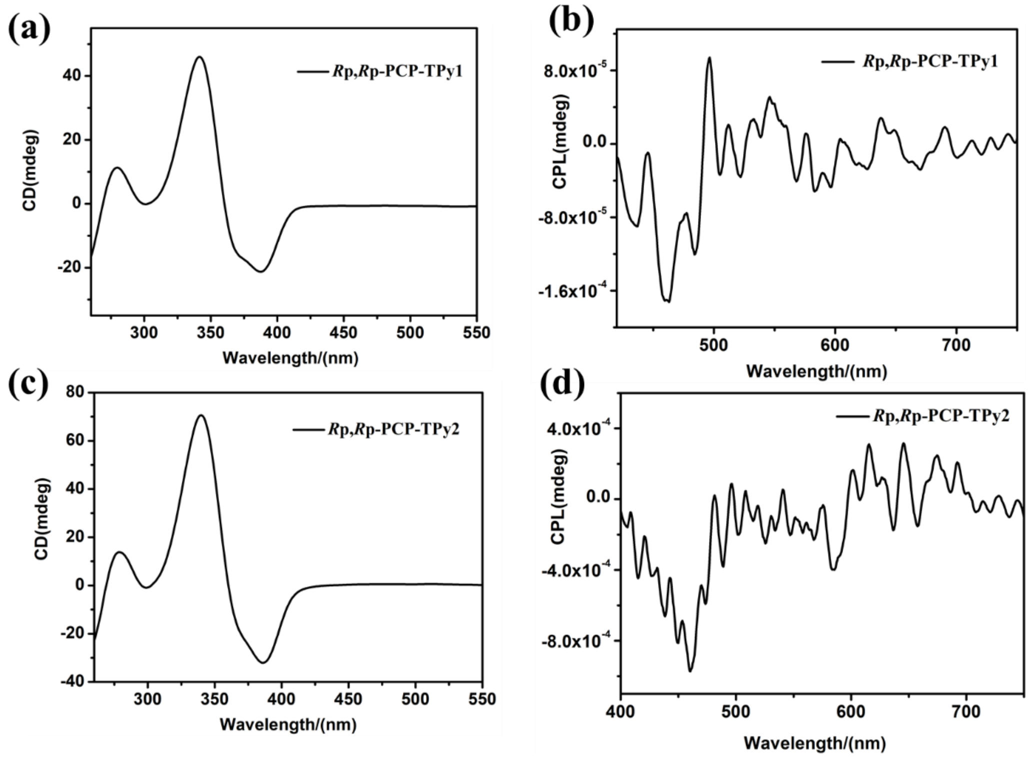

Supplementary Fig 52. CD spectra and CPL spectra. (a) The CD spectra of ***R*p,*R*p-PCP-TPy1** (1×10^{-5}M) in the mixture of THF/water (1:9, v/v), (b) The CPL spectra of ***R*p,*R*p-PCP-TPy1** (1×10^{-5}M) in the mixture of THF/water (1:9, v/v), (c) The CD spectra of ***R*p,*R*p-PCP-TPy2** (1×10^{-5}M) in the mixture of THF/water (1:9, v/v), (d) The CPL spectra of ***R*p,*R*p-PCP-TPy2** (1×10^{-5}M) in the mixture of THF/water (1:9, v/v).

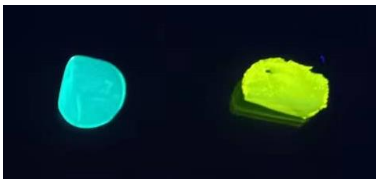

Supplementary Fig 53. Under 365nm UV lamp, the films of PCP-TPy1 (green) and PCP-TPy2 (yellow). (PMMA =10 mg/ml, **PCP-TPy1** = 1mg/ml, **PCP-TPy2** = 1mg/ml)

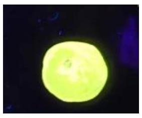

Supplementary Fig 54. Under 365nm UV lamp, the PCP-TPy2 film with a doping percentage of 0.075% NiR

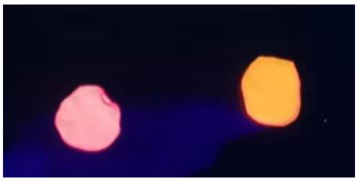

Supplementary Fig 55. Under 365nm UV lamp, the PCP-TPy1 film with a doping percentage of 0.5% NiR (pink); the PCP-TPy2 film with a doping percentage of 0.5% NiR (orange)

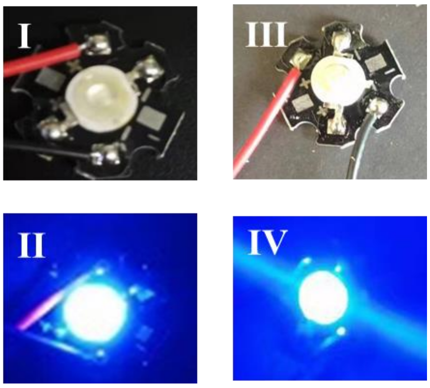

Supplementary Fig 56. (I), (II) Photo of a 460 nm LED chip integrated with PCP-TPy1; (III), (IV) Photo of a 460 nm LED chip integrated with *R*p,*R*p-PCP-TPy1

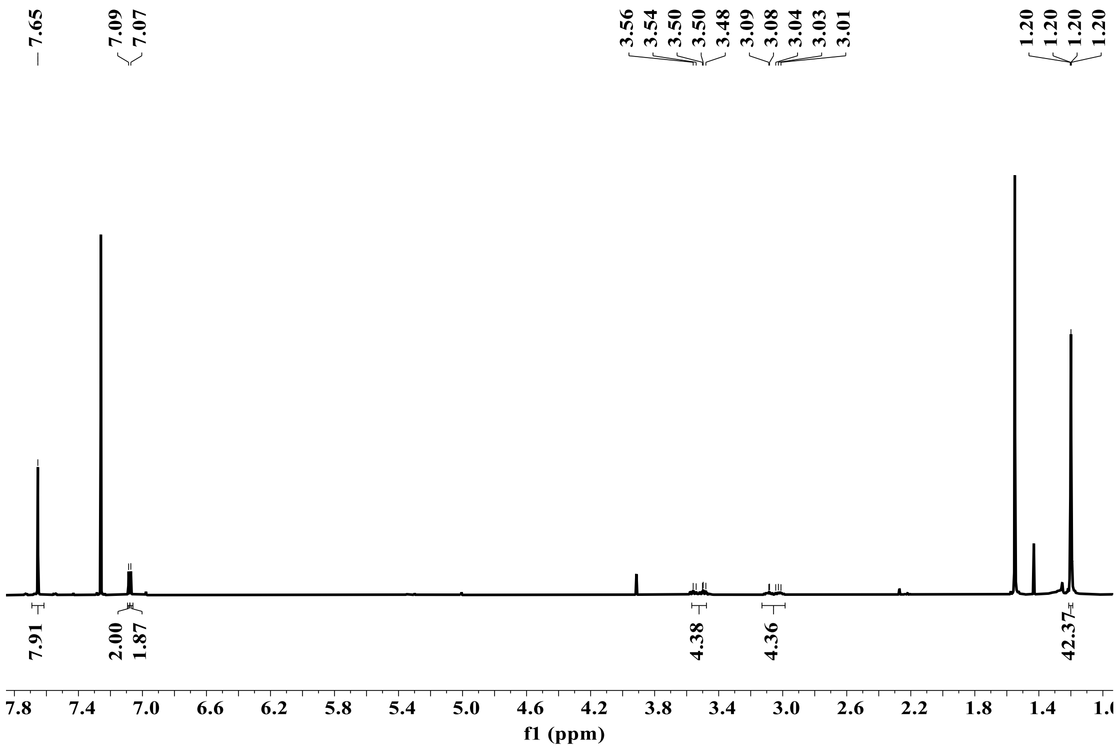

Supplementary Fig 57. ^{1}H NMR of Compound 1, $CDCl_3$, 298 K

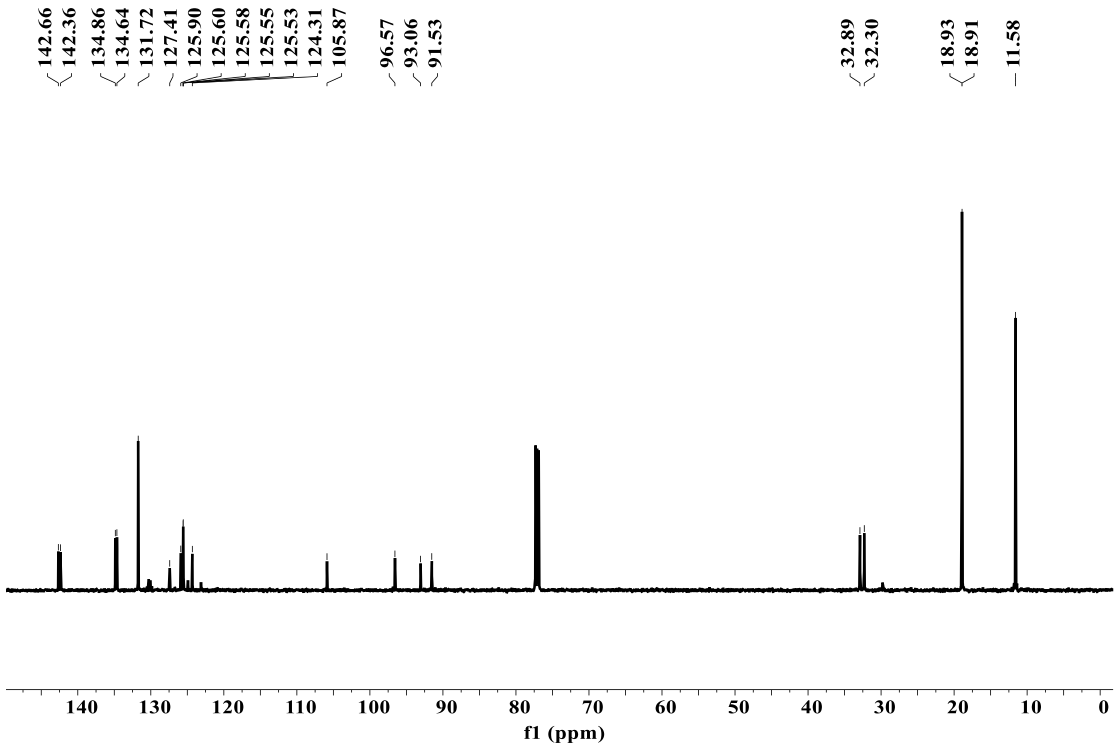

Supplementary Fig 58. ^{13}C NMR of Compound 1, $CDCl_3$, 298 K

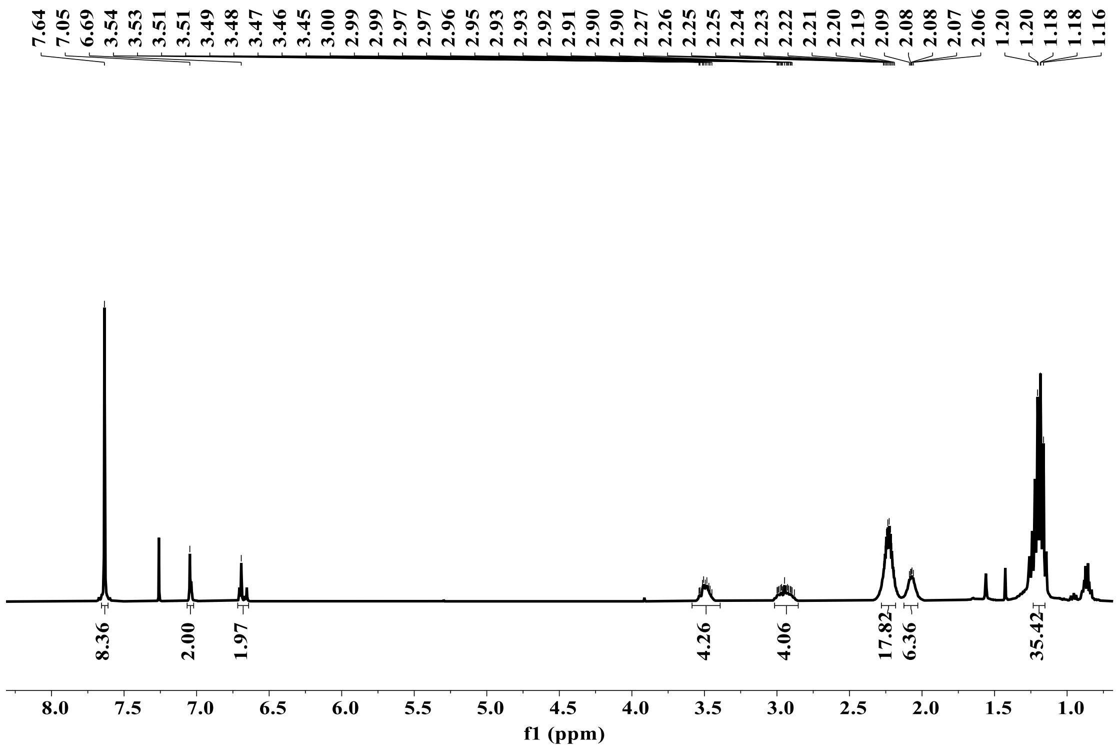

Supplementary Fig 59. ^1H **NMR of Compound 3,** $CDCl_3$**, 298 K**

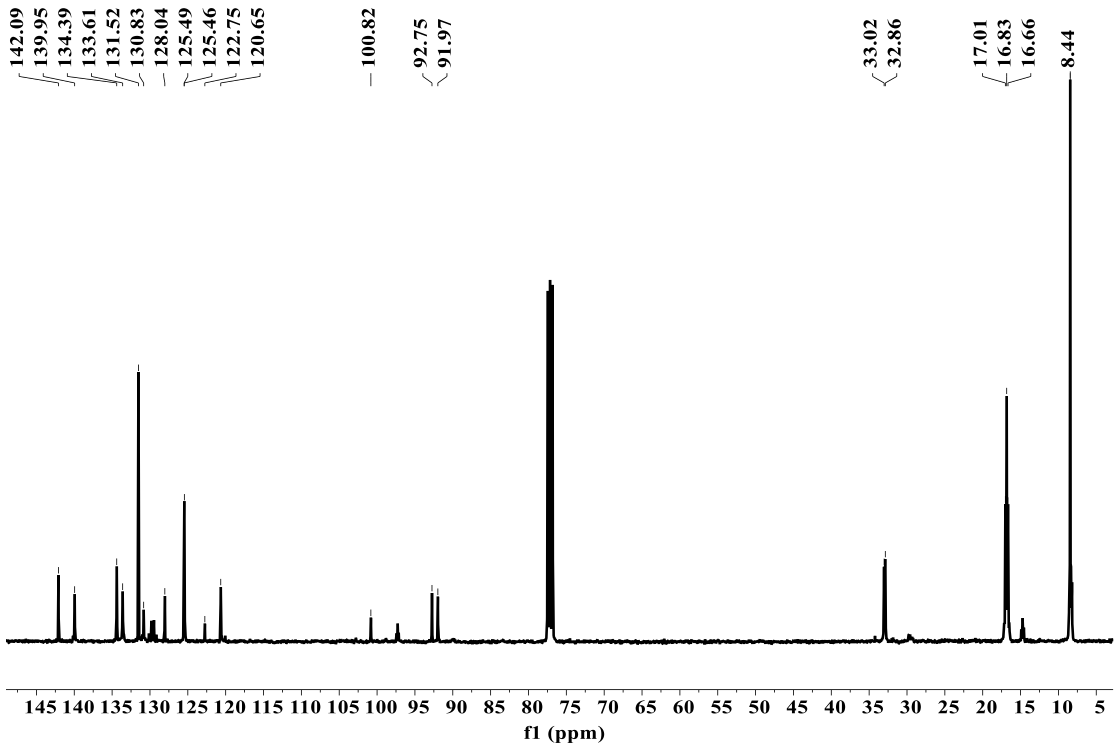

Supplementary Fig 60. ^{13}C **NMR of Compound 3,** $CDCl_3$**, 298 K**

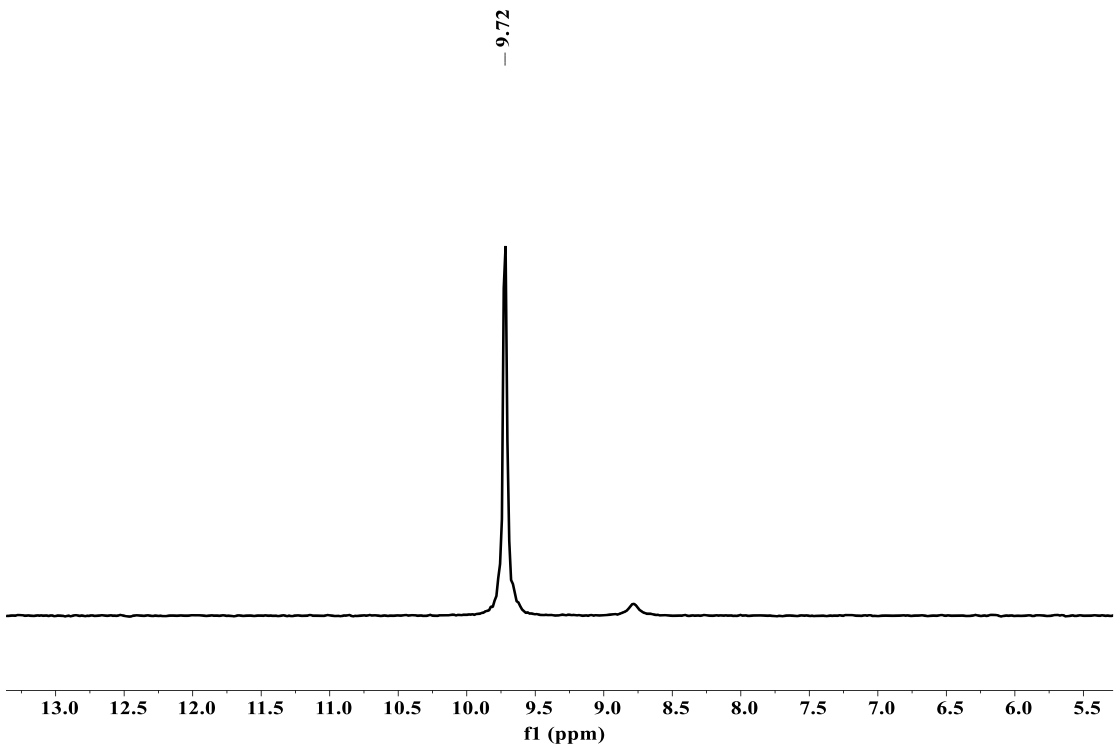

Supplementary Fig 61. ^{31}P **NMR of Compound 3,** $CDCl_3$**, 298 K**

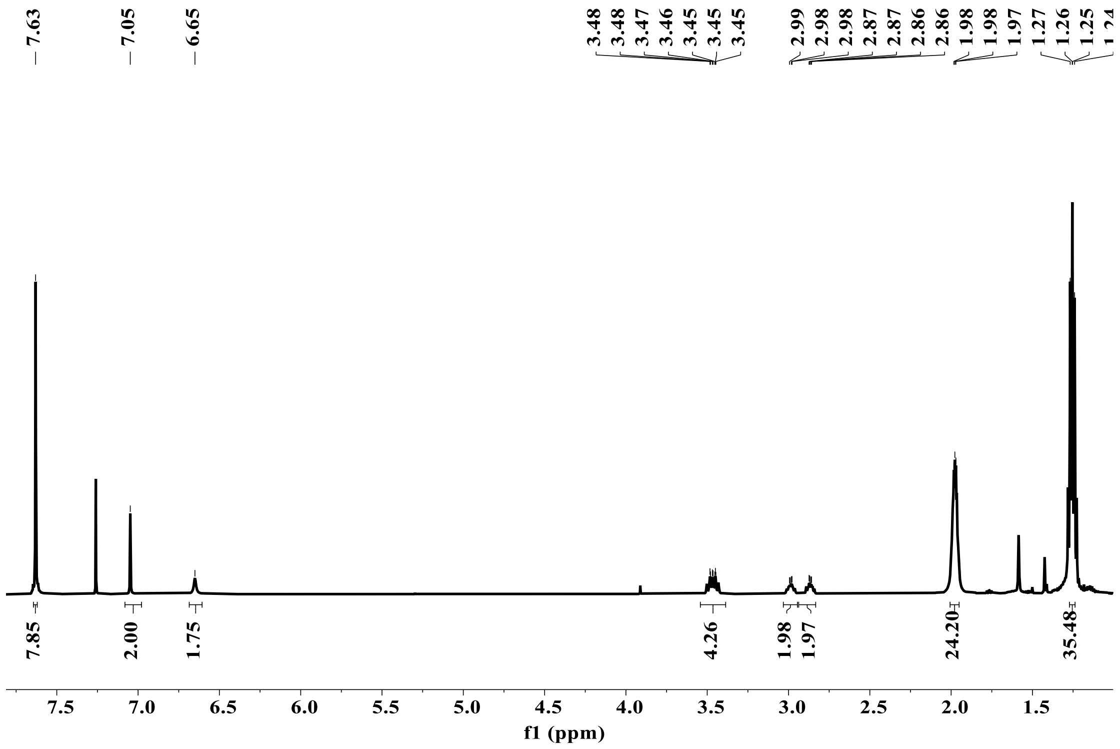

Supplementary Fig 62. ^{1}H **NMR of** ***rac*****-PCP,** $CDCl_3$**, 298 K**

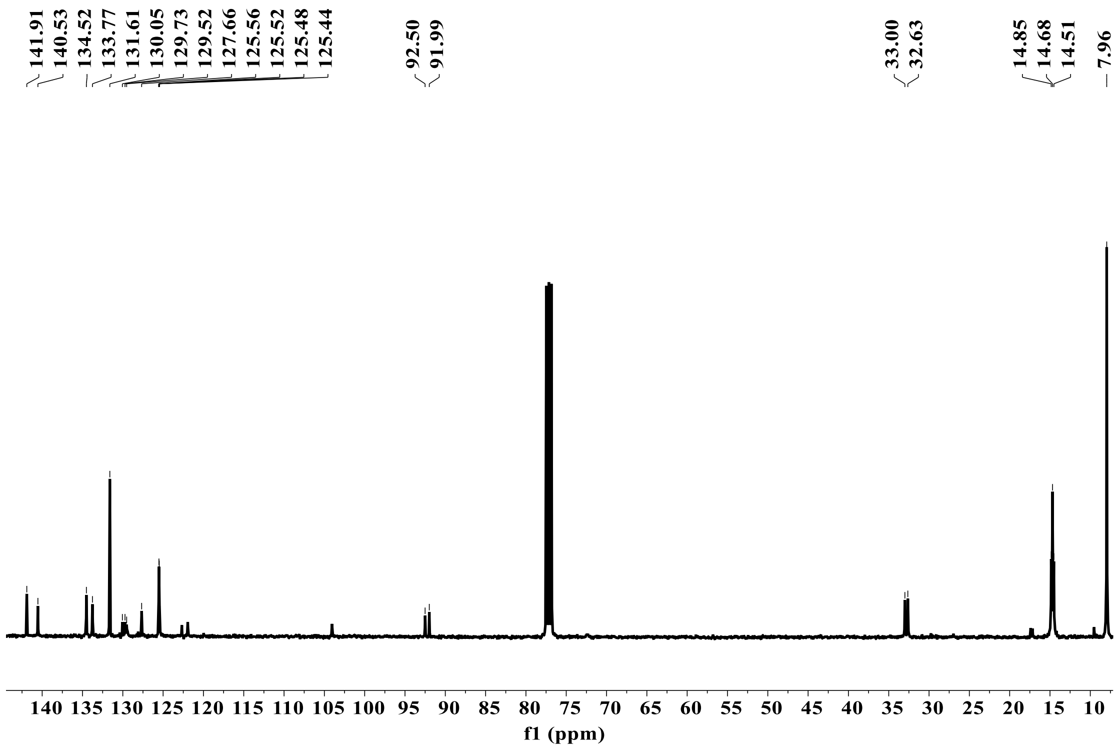

Supplementary Fig 63. ^{13}C **NMR of** ***rac*****-PCP,** $CDCl_3$**, 298 K**

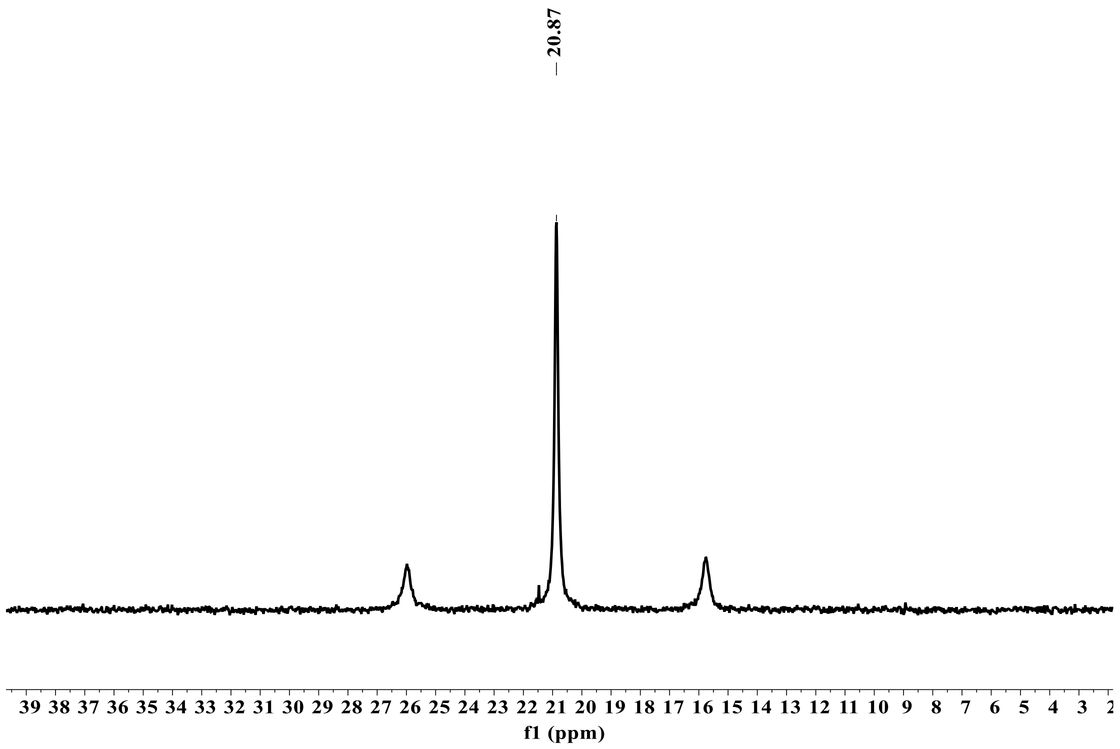

Supplementary Fig 64. ^{31}P **NMR of** ***rac*****-PCP,** $CDCl_3$**, 298 K**

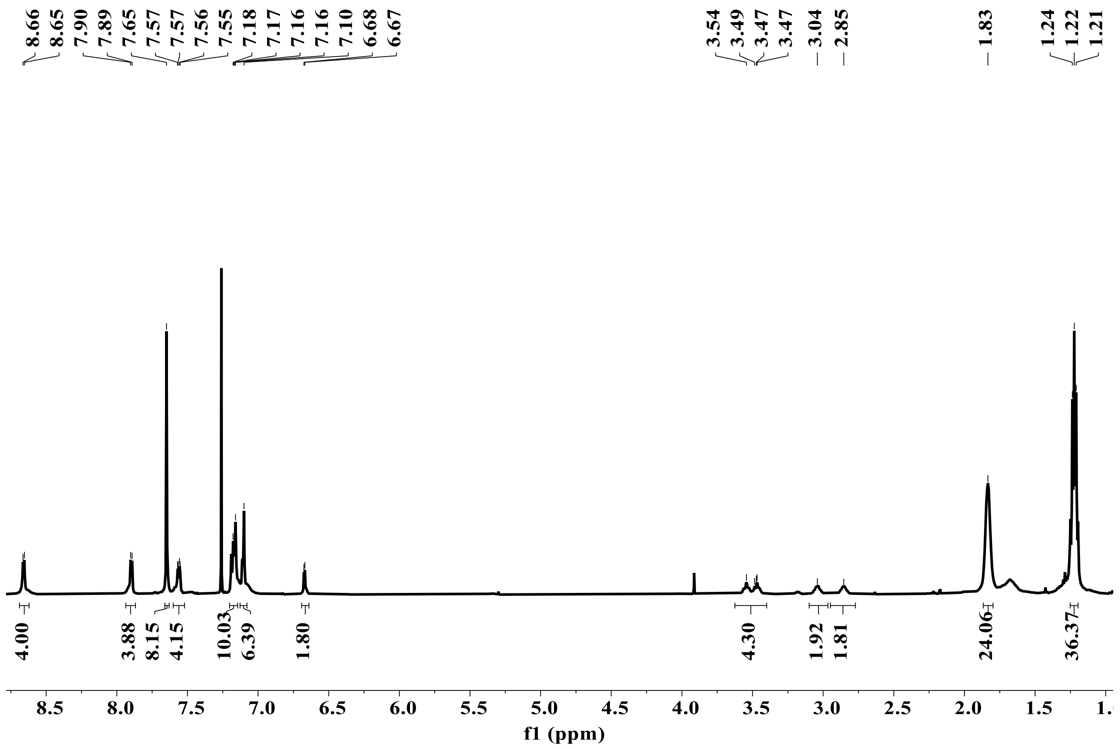

Supplementary Fig 65. **^{1}H NMR of PCP-TPy1, $CDCl_3$, 298 K**

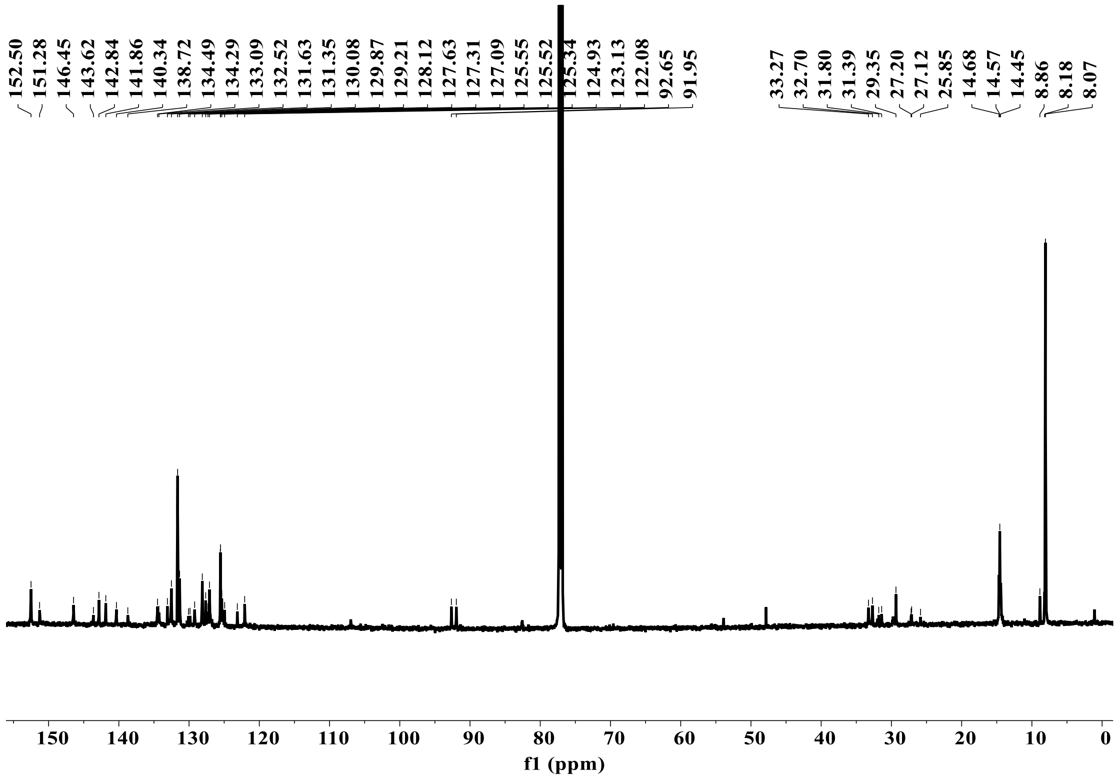

Supplementary Fig 66. **^{13}C NMR of PCP-TPy1, $CDCl_3$, 298 K**

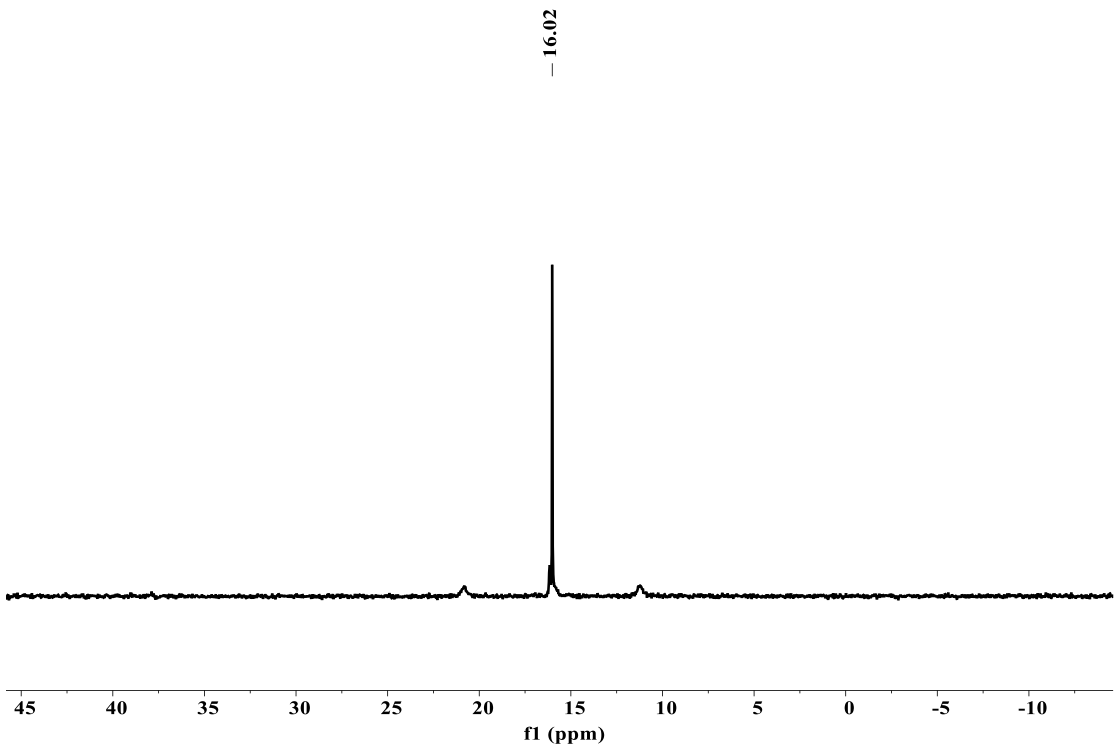

Supplementary Fig 67. ^{31}P NMR of PCP-TPy1, $CDCl_3$, 298 K

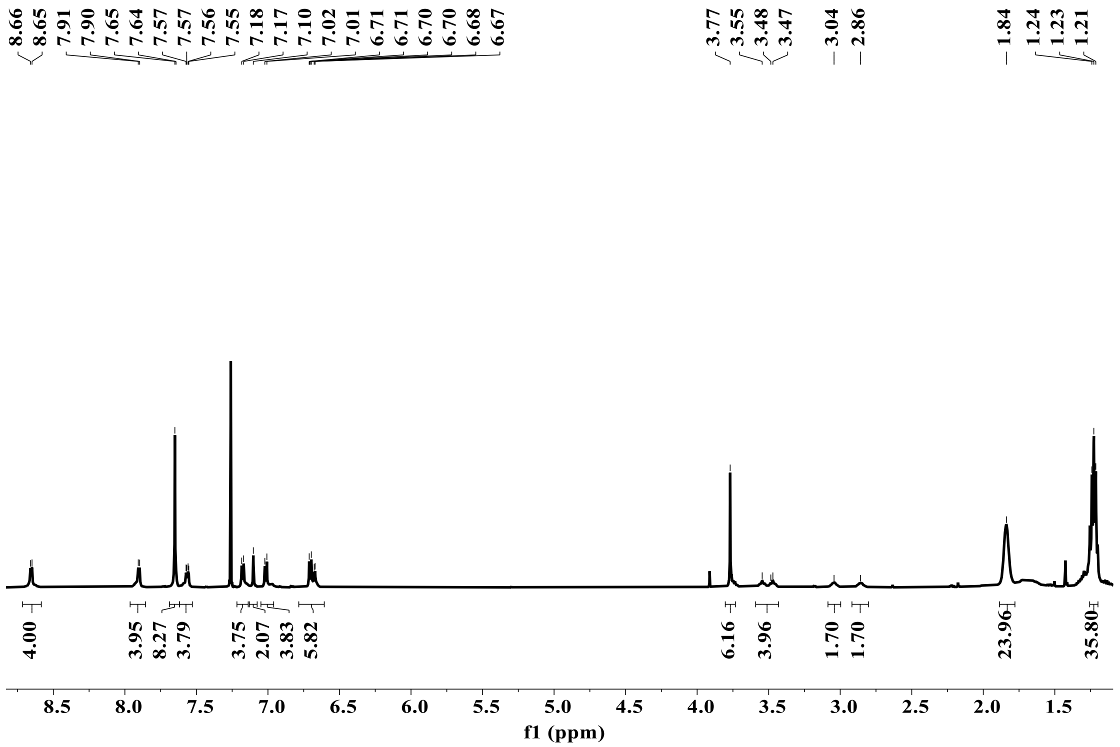

Supplementary Fig 68. ^{1}H NMR of PCP-TPy2, $CDCl_3$, 298 K

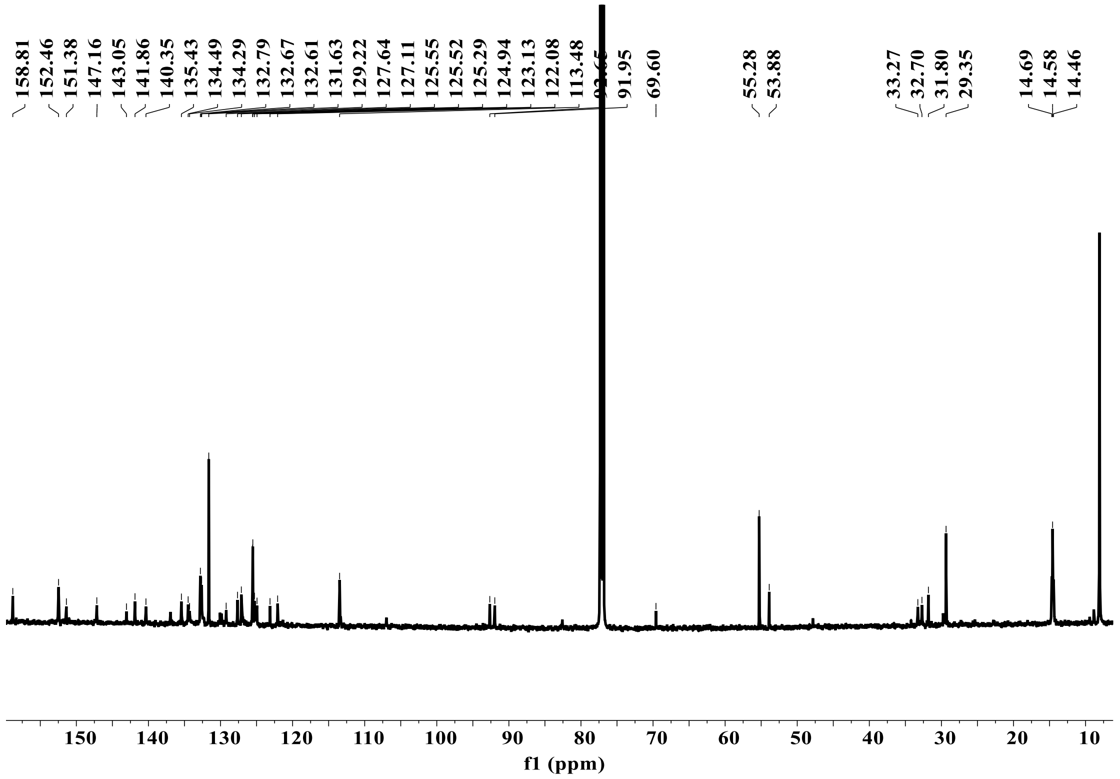

Supplementary Fig 69. ^{13}C NMR of PCP-TPy2, $CDCl_3$, 298 K

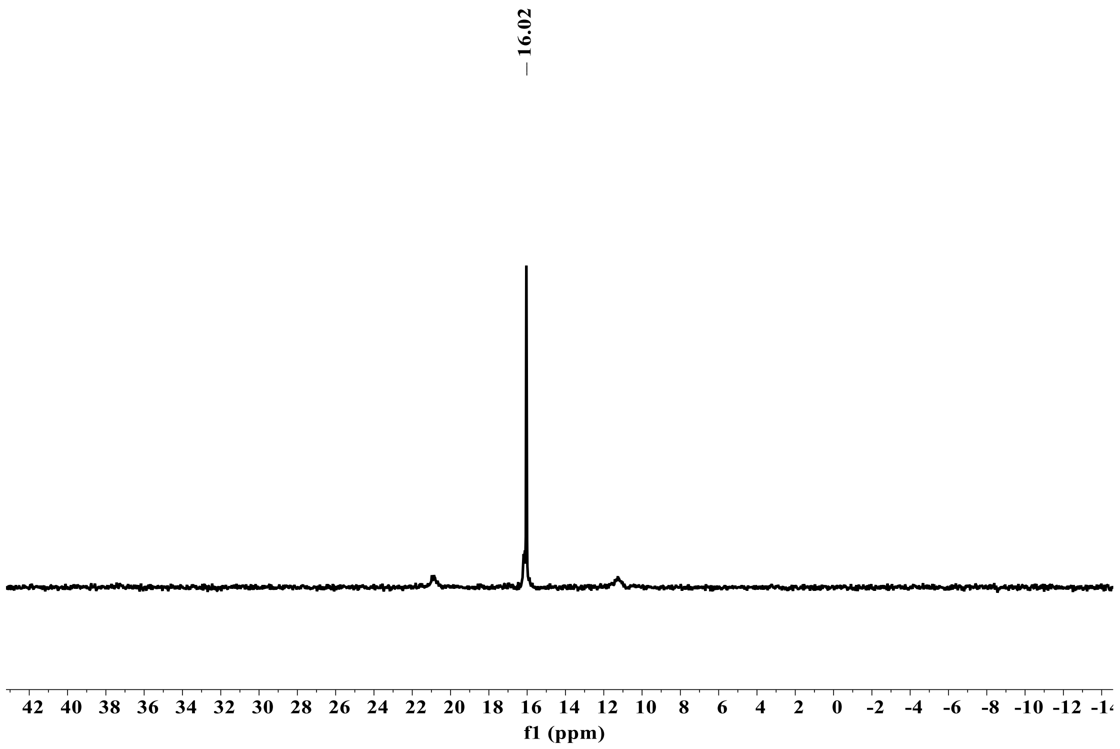

Supplementary Fig 70. ^{31}P NMR of PCP-TPy2, $CDCl_3$, 298 K

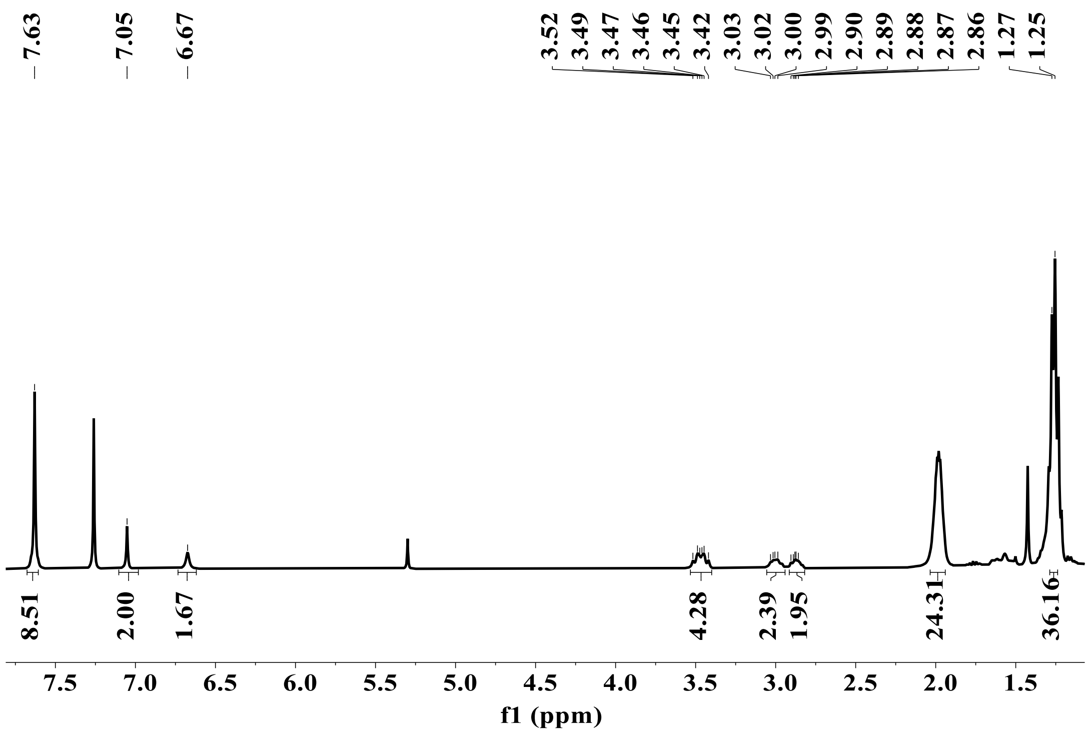

Supplementary Fig 71. ^{1}H **NMR of** ***R*****p-PCP, $CDCl_3$, 298 K**

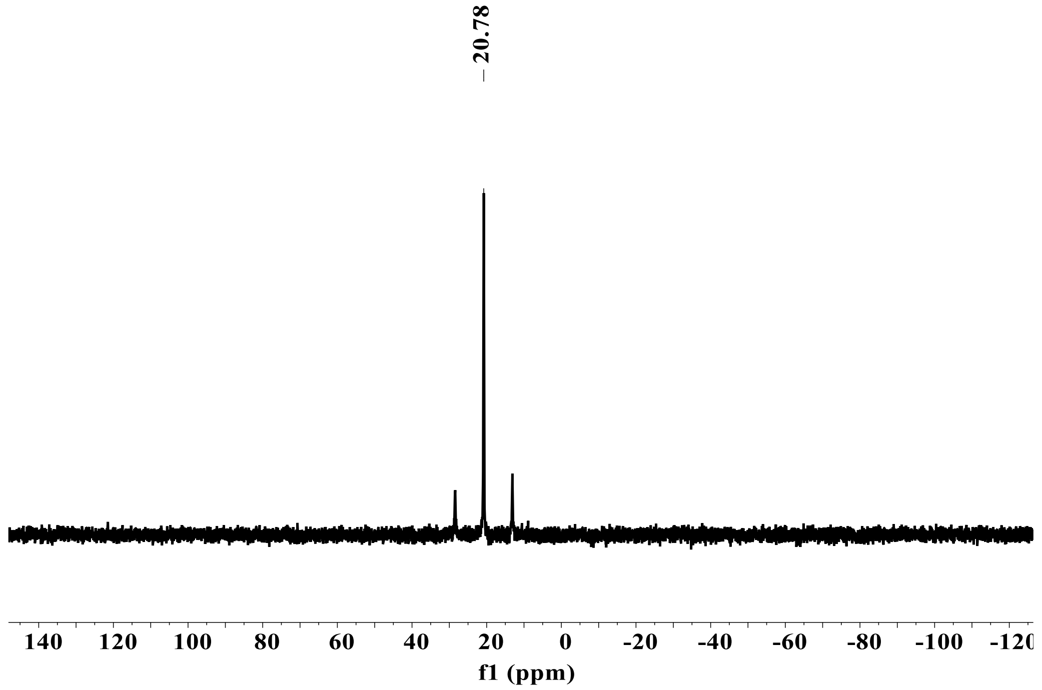

Supplementary Fig 72. ^{31}P **NMR of** ***R*****p-PCP, $CDCl_3$, 298 K**

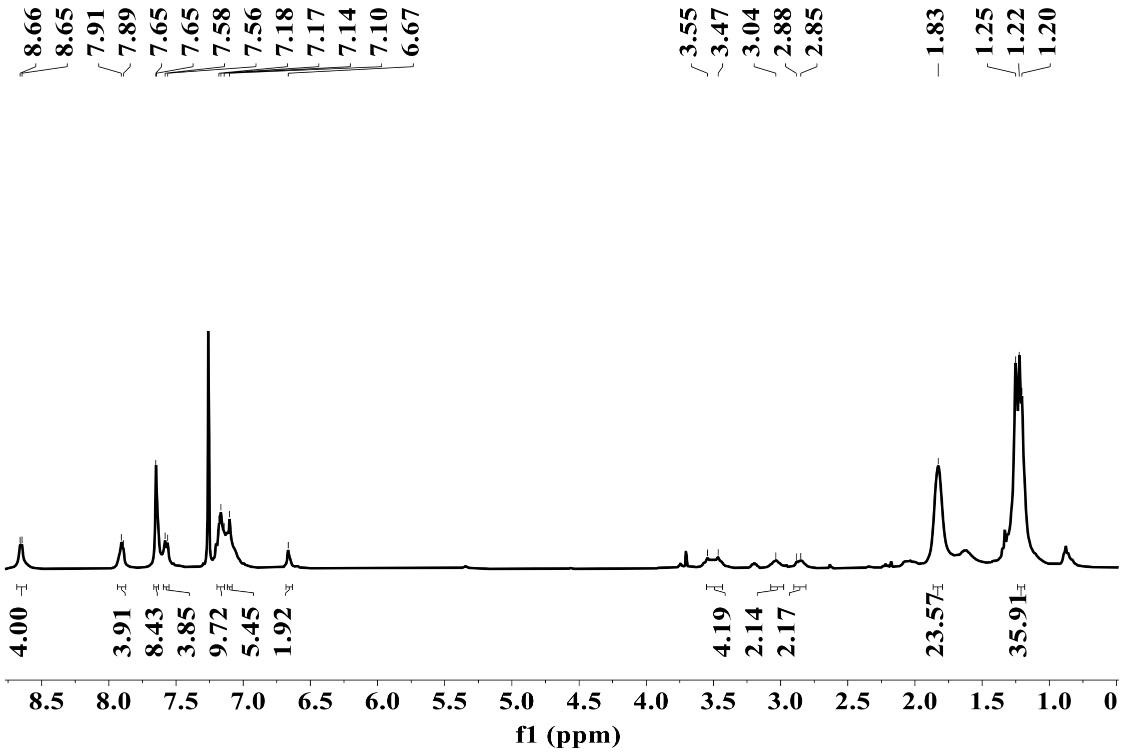

Supplementary Fig 73. **^{1}H NMR of *R*p,*R*p-PCP-TPy1, $CDCl_3$, 298 K**

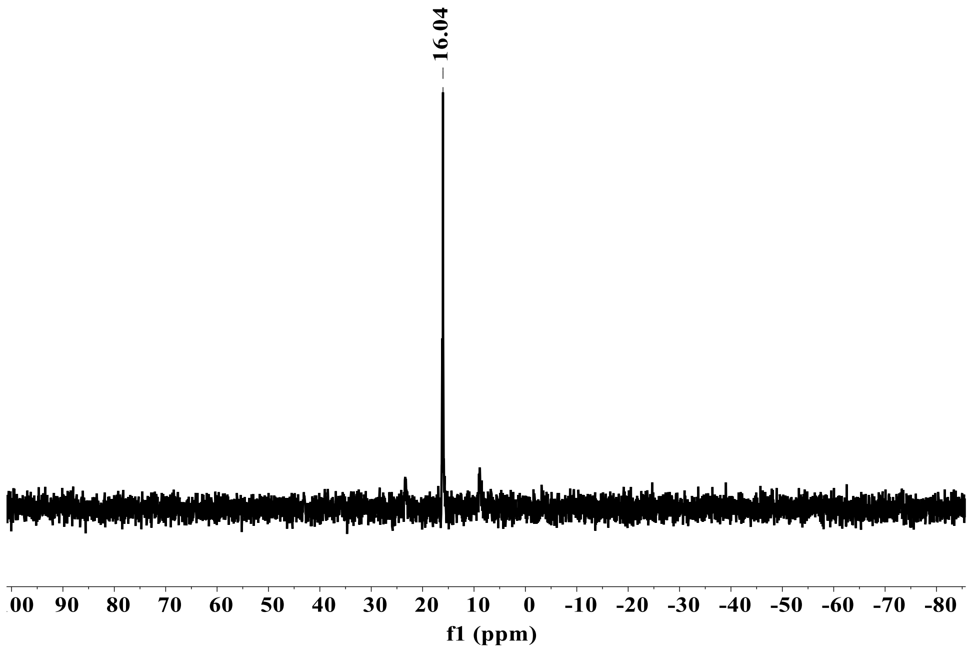

Supplementary Fig 74. **^{31}P NMR of *R*p,*R*p-PCP-TPy1, $CDCl_3$, 298 K**

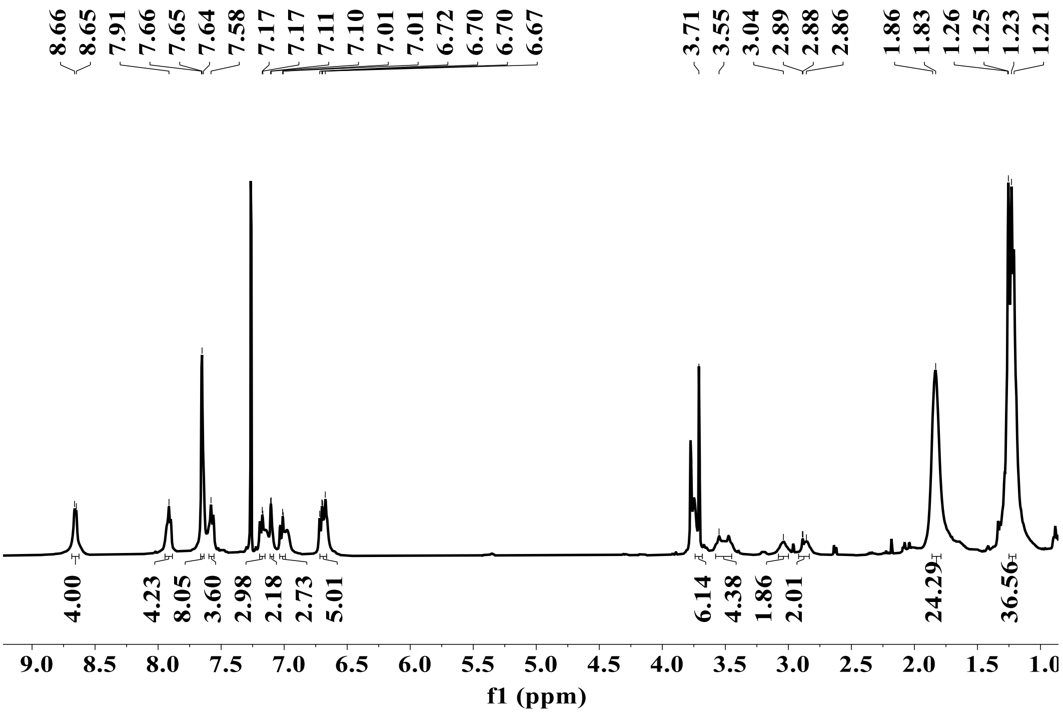

Supplementary Fig 75. ^{1}H NMR of *R*p,*R*p-PCP-TPy2, $CDCl_3$, 298 K

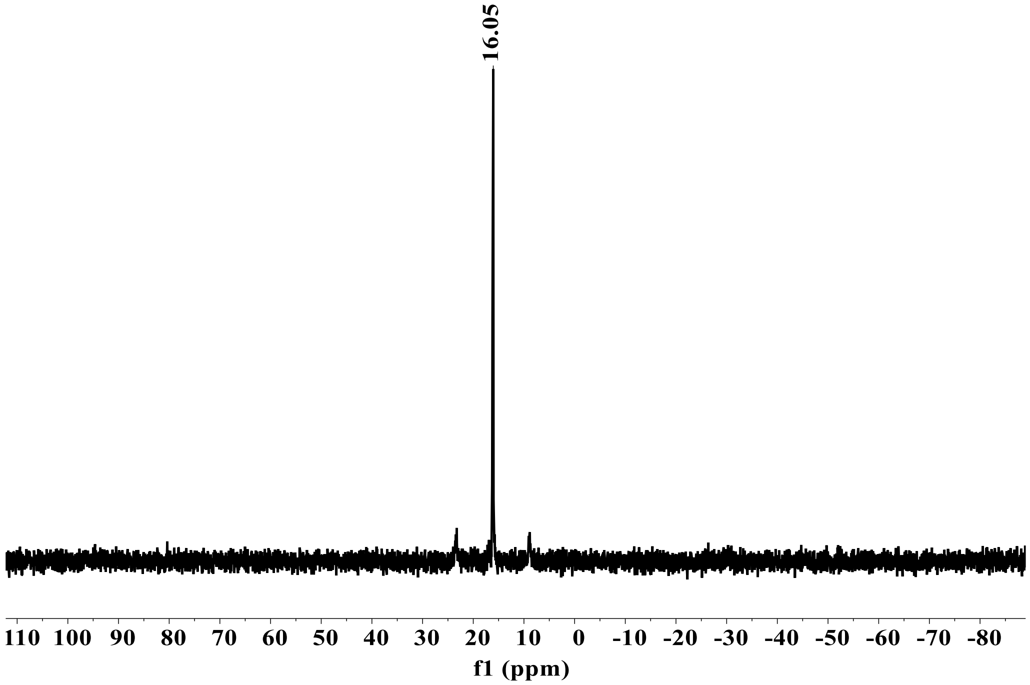

Supplementary Fig 76. ^{31}P NMR of *R*p,*R*p-PCP-TPy2, $CDCl_3$, 298 K

2. Supplementary Tables

Supplementary Table 1. Uncorrected and thermal-corrected(298K) energies of the *meso*-PCP-TPy1 and *R*p,*R*p-PCP-TPy1. (Hartree)[a]

Optimized structure	E(H)	G(H)	E_{pcm}(H)	G_{pcm}(H)
***meso*-PCP-TPy1**	-9930.55612340	-9927.937440	-9930.94985424	-9928.331171
***R*p,*R*p-PCP-TPy1**	-9930.55332060	-9927.934864	-9930.94932791	-9928.330871

a) *E*:electronic energy; *G*: sum of electronic and thermal free energies. E_{pcm}: electronic energy corrected by PCM; *G*: sum of electronic and thermal free energies corrected by PCM.

Supplementary Table 2. Optical properties of PCP-TPy1 and PCP-TPy2 in DCM/Hexane system

	λ_{abs} [a,b]（nm）	λ_{ex} [a,c]（nm）	solvent	Φ_F (%) [d]
**PCP-TPy1**	365	435	DCM	0.6
**PCP-TPy1**	386	505	DCM/Hexane (1/9,V/V)	5.2
**PCP-TPy2**	377	450/525	DCM	1.0
**PCP-TPy2**	387	560	DCM/Hexane (1/9,V/V)	13.2

a UV-Vis absorption and fluorescence spectra were measured in DCM (1×10^{-5}M) and DCM/Hexane (1/9,V/V) (1×10^{-5}M) at room temperature. b Absorption maximum at the longest wavelength. c Emission maximum (λ_{ex}=highest intensity of absorption plus 10 nm). d Fluorescence quantum yield.

Supplementary Table 3. Energy transfer efficiency comparison table of one-step systems.

One-Step Energy transfer systems	Energy transfer efficiency
**4[a]+EsY** system[6]	65.0%
**(3-4)–6** system[7]	53.1%
**6a / NAP** system[8]	83.0%
**DBT@CSU** system[9]	64.3%
**PCP-TPy1/EsY** system (this work)	63.5%
***R*p,*R*p-PCP-TPy1/EsY** system (this work)	83.8%
**PCP-TPy1/NiR** system (this work)	67.3%
***R*p,*R*p-PCP-TPy1/NiR** system (this work)	73.1%
**PCP-TPy2/NiR** system (this work)	74.8%
***R*p,*R*p-PCP-TPy2/NiR** system (this work)	79.9%

Supplementary Table 4. Energy transfer efficiency comparison table of Sequential systems.

Sequential Energy transfer systems	Energy transfer efficiency
**M1-EsY-SR101** system[10]	59.5%
**PPTA-BSC4+EsY+SR101**[11] system	40.8%
**PyTPE/WP5:SR101:AlPcS4** system[12]	84.2%
**WP5 ⊃ TPEDA - EsY –NiR** system[13]	74.7%
**3a[a]-ESY-NiR system**[14]	64.6%
**3a[b]-ESY-NiR system**[14]	29.7%
**PCP-TPy1/EsY/NiR** system (this work)	89.3%
***R*p,*R*p-PCP-TPy1/EsY/NiR** system (this work)	87.8%

Supplementary Table 5. Fluorescence lifetimes of PCP-TPy1 and *R*p,*R*p-PCP-TPy2 in aggregated states and their ALHSs.

systems	τ/ns
**PCP-TPy2**	1.25±0.13
***R*p,*R*p-PCP-TPy2**	1.20±0.15
**PCP-TPy2/NiR (200:1)**	0.64±0.10
***R*p,*R*p-PCP-TPy2/NiR (200:1)**	0.54±0.13

Supplementary Table 6. G_{abs} and G_{lum} of *R*p,*R*p-PCP-TPy1 and *R*p,*R*p-PCP-TPy2 in different solutions.

	solvent	$\lvert g_{abs} \rvert$	$\lvert g_{lum} \rvert$
***R*p,*R*p-PCP-TPy1**	THF	2.0×10^{-3}	1.7×10^{-3}
***R*p,*R*p-PCP-TPy1**	THF/Water (1/9,V/V)	1.7×10^{-3}	1.4×10^{-3}
***R*p,*R*p-PCP-TPy2**	THF	2.4×10^{-3}	2.4×10^{-3}
***R*p,*R*p-PCP-TPy2**	THF/Water (1/9,V/V)	2.2×10^{-3}	4.4×10^{-4}

Supplementary Table 7. Cartesian coordinates of optimized species. Optimized S0 geometry of heterochiral helicate (at the B3LYP-D3/6-31G(d))

Center Number	Atomic Number	Atomic Type	Coordinates (Angstroms)		
			X	Y	Z
1	C	0	-17.048462	-0.835044	1.683992
2	C	0	-18.174465	-1.296238	1.657794
3	C	0	-19.508497	-1.782577	1.605325
4	C	0	-19.783867	-3.161751	1.684913
5	C	0	-21.096638	-3.616932	1.613155
6	C	0	-22.148960	-2.709313	1.462597
7	C	0	-21.884530	-1.339329	1.384915

8	C	0	-20.575680	-0.873409	1.456512
9	H	0	-18.962461	-3.861375	1.803436
10	H	0	-21.302377	-4.681130	1.676191
11	H	0	-23.171858	-3.069242	1.408893
12	H	0	-22.699785	-0.631308	1.270796
13	H	0	-20.363222	0.187867	1.391798
14	C	0	-13.311260	1.083975	1.287402
15	C	0	-13.374731	-0.265434	1.682713
16	C	0	-14.574857	0.968931	-1.710888
17	C	0	-13.375086	0.265564	-1.682024
18	C	0	-13.311516	-1.083846	-1.286732
19	C	0	-14.508509	-1.757780	-0.936320
20	C	0	-15.774148	0.219177	-1.623088
21	C	0	-15.711380	-1.140693	-1.258474
22	C	0	-14.509179	-2.931441	0.014225
23	C	0	-14.587606	-2.470082	1.550061
24	C	0	-14.574480	-0.968829	1.711790
25	C	0	-14.508332	1.757884	0.937205
26	C	0	-15.711130	1.140768	1.259570
27	C	0	-15.773803	-0.219103	1.624197
28	C	0	-14.587978	2.470185	-1.549156
29	C	0	-14.509212	2.931545	-0.013338
30	C	0	-12.037287	1.697914	1.109619
31	H	0	-12.441129	-0.804018	1.811820
32	H	0	-12.441519	0.804172	-1.811286
33	C	0	-12.037499	-1.697763	-1.109182
34	H	0	-13.606241	-3.534055	-0.120474
35	H	0	-15.368528	-3.576234	-0.189326
36	H	0	-13.744169	-2.914763	2.088010
37	H	0	-15.501023	-2.883291	1.983770
38	H	0	-16.644701	1.650129	1.046446
39	H	0	-15.501502	2.883374	-1.982660
40	H	0	-13.744674	2.914884	-2.087298
41	H	0	-15.368519	3.576334	0.190402
42	H	0	-13.606248	3.534164	0.121163
43	C	0	-10.940432	2.227622	0.982606
44	C	0	-10.940619	-2.227464	-0.982354
45	C	0	-17.048832	0.835089	-1.682682
46	C	0	-18.174858	1.296219	-1.656330
47	C	0	-19.508934	1.782415	-1.603626
48	C	0	-20.576049	0.873099	-1.455234
49	C	0	-21.884935	1.338889	-1.383417
50	C	0	-22.149467	2.708890	-1.460449
51	C	0	-21.097213	3.616657	-1.610585
52	C	0	-19.784410	3.161606	-1.682568

53	H	0	-20.363513	-0.188193	-1.391016
54	H	0	-22.700142	0.630757	-1.269639
55	H	0	-23.172392	3.068717	-1.406571
56	H	0	-21.303029	4.680869	-1.673118
57	H	0	-18.963056	3.861345	-1.800766
58	H	0	-16.644900	-1.650078	-1.045186
59	P	0	-9.205120	2.633261	-1.384047
60	C	0	-10.678928	3.305464	-2.235316
61	C	0	-9.239228	0.834742	-1.726975
62	C	0	-7.777951	3.265103	-2.355089
63	H	0	-11.565209	2.970334	-1.693335
64	H	0	-10.651691	4.398762	-2.211046
65	H	0	-10.109787	0.379628	-1.249549
66	H	0	-8.344031	0.355399	-1.319370
67	H	0	-7.881806	3.000711	-3.412240
68	H	0	-6.844903	2.843360	-1.970295
69	H	0	-10.725606	2.966900	-3.275294
70	H	0	-9.285241	0.650445	-2.805205
71	H	0	-7.724226	4.353930	-2.265746
72	P	0	-9.598941	3.658460	3.151559
73	C	0	-11.131528	4.631261	3.388355
74	C	0	-8.283154	4.615240	4.003783
75	C	0	-9.835101	2.161158	4.180610
76	H	0	-11.048255	5.589722	2.868027
77	H	0	-11.963327	4.070604	2.954859
78	H	0	-8.142224	5.579338	3.507251
79	H	0	-7.338311	4.065218	3.964221
80	H	0	-10.091286	2.433274	5.209470
81	H	0	-8.917920	1.565040	4.181715
82	H	0	-11.317289	4.811876	4.451957
83	H	0	-8.551206	4.792617	5.050070
84	H	0	-10.643424	1.572628	3.740141
85	C	0	-4.943948	5.654534	0.529976
86	C	0	-4.979787	4.256100	0.690756
87	C	0	-6.191067	3.597479	0.817660
88	N	0	-7.377836	4.239811	0.790276
89	C	0	-7.363267	5.582813	0.645949
90	C	0	-6.190576	6.309002	0.519179
91	H	0	-4.059745	3.685028	0.742367
92	H	0	-6.248900	7.382019	0.376710
93	C	0	-3.682418	6.390914	0.368925
94	H	0	-6.241024	2.522153	0.949261
95	H	0	-8.333301	6.066939	0.620204
96	C	0	-2.589741	5.816027	-0.303089
97	C	0	-1.414646	6.532442	-0.494691

98	C	0	-1.278718	7.850769	-0.021807
99	C	0	-2.360089	8.403916	0.691716
100	C	0	-3.539442	7.698292	0.870435
101	C	0	-0.030536	8.620868	-0.248108
102	C	0	-0.045607	9.955089	-0.569649
103	C	0	-1.275535	10.650061	-1.021491
104	C	0	-2.104331	10.083594	-2.004588
105	C	0	-3.243068	10.753809	-2.442591
106	C	0	-3.577237	11.997494	-1.900244
107	C	0	-2.755737	12.575295	-0.929429
108	C	0	-1.605696	11.914255	-0.503926
109	C	0	4.954933	5.706004	0.222721
110	C	0	6.169028	6.255532	-0.233547
111	C	0	7.349448	5.540600	-0.118086
112	N	0	7.404281	4.305593	0.425924
113	C	0	6.252520	3.766603	0.878348
114	C	0	5.035607	4.421949	0.795704
115	H	0	6.200180	7.253588	-0.654902
116	H	0	4.143476	3.921124	1.153789
117	C	0	3.681975	6.430982	0.102478
118	H	0	8.294361	5.951151	-0.456723
119	H	0	6.333895	2.774578	1.309013
120	C	0	3.466594	7.332860	-0.956801
121	C	0	2.271546	8.025040	-1.071255
122	C	0	1.242229	7.865667	-0.124042
123	C	0	1.449857	6.945490	0.919429
124	C	0	2.644425	6.244478	1.033431
125	C	0	1.170422	10.800433	-0.493578
126	C	0	1.469344	11.705194	-1.526988
127	C	0	2.606765	12.506376	-1.459962
128	C	0	3.447245	12.438108	-0.346241
129	C	0	3.144639	11.562749	0.699960
130	C	0	2.018405	10.747997	0.625806
131	H	0	-0.595920	6.079512	-1.044477
132	H	0	-2.269901	9.403587	1.099519
133	H	0	-4.346083	8.152172	1.438143
134	H	0	-1.839280	9.121709	-2.431789
135	H	0	-3.863857	10.313799	-3.217679
136	H	0	-4.463828	12.521655	-2.244079
137	H	0	-3.004868	13.546308	-0.512598
138	H	0	-0.958743	12.370736	0.238555
139	H	0	4.227046	7.465514	-1.720150
140	H	0	2.125502	8.706444	-1.901150
141	H	0	0.672554	6.802935	1.663439
142	H	0	2.789644	5.582423	1.882069

143	H	0	0.807662	11.770533	-2.384952
144	H	0	2.831184	13.192943	-2.270529
145	H	0	4.323726	13.076250	-0.286605
146	H	0	3.779588	11.526738	1.580359
147	H	0	1.777389	10.076588	1.443583
148	H	0	-2.678813	4.819583	-0.726468
149	P	0	-9.599550	-3.658287	-3.151579
150	C	0	-9.835950	-2.160975	-4.180559
151	C	0	-11.132164	-4.631117	-3.388074
152	C	0	-8.283914	-4.615022	-4.004087
153	H	0	-10.644179	-1.572454	-3.739905
154	H	0	-8.918772	-1.564852	-4.181863
155	H	0	-11.963885	-4.070485	-2.954397
156	H	0	-11.048765	-5.589585	-2.867779
157	H	0	-8.552199	-4.792428	-5.050309
158	H	0	-8.142822	-5.579107	-3.507573
159	H	0	-10.092361	-2.433081	-5.209366
160	H	0	-11.318140	-4.811718	-4.451641
161	H	0	-7.339089	-4.064953	-3.964752
162	P	0	-9.204848	-2.633104	1.383961
163	C	0	-10.678493	-3.305279	2.235533
164	C	0	-9.238872	-0.834578	1.726860
165	C	0	-7.777498	-3.264940	2.354741
166	H	0	-11.564880	-2.970146	1.693728
167	H	0	-10.651275	-4.398578	2.211273
168	H	0	-10.109524	-0.379470	1.249602
169	H	0	-8.343752	-0.355258	1.319057
170	H	0	-7.881158	-3.000538	3.411909
171	H	0	-6.844519	-2.843201	1.969772
172	H	0	-10.724956	-2.966700	3.275515
173	H	0	-9.284660	-0.650249	2.805094
174	H	0	-7.723789	-4.353768	2.265398
175	C	0	-4.944094	-5.654411	-0.530621
176	C	0	-6.190721	-6.308881	-0.519785
177	C	0	-7.363421	-5.582686	-0.646439
178	N	0	-7.378000	-4.239675	-0.790691
179	C	0	-6.191231	-3.597344	-0.818132
180	C	0	-4.979944	-4.255971	-0.691339
181	H	0	-6.249036	-7.381905	-0.377368
182	H	0	-4.059906	-3.684895	-0.742985
183	C	0	-3.682557	-6.390799	-0.369664
184	H	0	-8.333452	-6.066815	-0.620653
185	H	0	-6.241197	-2.522012	-0.949678
186	C	0	-3.539598	-7.698145	-0.871261
187	C	0	-2.360245	-8.403786	-0.692610

188	C	0	-1.278860	-7.850694	0.020935
189	C	0	-1.414769	-6.532393	0.493898
190	C	0	-2.589863	-5.815958	0.302360
191	C	0	-0.030685	-8.620824	0.247186
192	C	0	-0.045769	-9.955061	0.568664
193	C	0	-1.275684	-10.650045	1.020526
194	C	0	-1.605902	-11.914189	0.502876
195	C	0	-2.755927	-12.575244	0.928399
196	C	0	-3.577351	-11.997512	1.899319
197	C	0	-3.243121	-10.753880	2.441752
198	C	0	-2.104402	-10.083648	2.003729
199	C	0	4.954854	-5.706061	-0.223540
200	C	0	6.168922	-6.255631	0.232752
201	C	0	7.349364	-5.540730	0.117337
202	N	0	7.404247	-4.305717	-0.426652
203	C	0	6.252517	-3.766691	-0.879110
204	C	0	5.035582	-4.422004	-0.796511
205	H	0	6.200034	-7.253696	0.654089
206	H	0	4.143477	-3.921152	-1.154621
207	C	0	3.681873	-6.431005	-0.103326
208	H	0	8.294254	-5.951310	0.456001
209	H	0	6.333933	-2.774665	-1.309764
210	C	0	3.466462	-7.332901	0.955933
211	C	0	2.271399	-8.025057	1.070361
212	C	0	1.242093	-7.865641	0.123143
213	C	0	1.449748	-6.945444	-0.920305
214	C	0	2.644334	-6.244458	-1.034282
215	C	0	1.170249	-10.800415	0.492508
216	C	0	1.469209	-11.705216	1.525872
217	C	0	2.606621	-12.506405	1.458767
218	C	0	3.447053	-12.438103	0.345013
219	C	0	3.144409	-11.562703	-0.701143
220	C	0	2.018185	-10.747945	-0.626910
221	H	0	-4.346254	-8.151984	-1.438981
222	H	0	-2.270071	-9.403430	-1.100480
223	H	0	-0.596032	-6.079502	1.043698
224	H	0	-2.678921	-4.819540	0.725803
225	H	0	-0.959008	-12.370617	-0.239688
226	H	0	-3.005104	-13.546216	0.511500
227	H	0	-4.463928	-12.521686	2.243169
228	H	0	-3.863848	-10.313926	3.216921
229	H	0	-1.839304	-9.121805	2.430998
230	H	0	4.226903	-7.465587	1.719287
231	H	0	2.125334	-8.706480	1.900237
232	H	0	0.672455	-6.802857	-1.664318

233	H	0	2.789575	-5.582388	-1.882905
234	H	0	0.807564	-11.770582	2.383863
235	H	0	2.831069	-13.193003	2.269299
236	H	0	4.323526	-13.076250	0.285315
237	H	0	3.779320	-11.526665	-1.581568
238	H	0	1.777138	-10.076504	-1.444653
239	P	0	9.139461	2.421465	-1.617863
240	C	0	7.649564	2.898199	-2.583314
241	C	0	9.206806	0.596187	-1.751193
242	C	0	10.554380	3.011552	-2.618727
243	H	0	6.749460	2.524078	-2.086263
244	H	0	7.578901	3.987798	-2.643977
245	H	0	8.336449	0.146858	-1.264139
246	H	0	10.102564	0.217073	-1.254788
247	H	0	10.552300	2.558261	-3.615195
248	H	0	11.475207	2.750197	-2.093135
249	H	0	7.696978	2.489845	-3.597855
250	H	0	9.225550	0.290647	-2.802593
251	H	0	10.510488	4.100194	-2.716223
252	P	0	9.725148	4.040599	2.720559
253	C	0	8.463936	5.151351	3.461237
254	C	0	11.288382	4.991080	2.772853
255	C	0	9.953929	2.695223	3.943099
256	H	0	8.326098	6.030022	2.824554
257	H	0	7.505854	4.629748	3.541876
258	H	0	11.211075	5.868342	2.124267
259	H	0	12.091687	4.350909	2.400620
260	H	0	10.244906	3.099641	4.917784
261	H	0	10.735731	2.029261	3.569887
262	H	0	8.776466	5.480401	4.457235
263	H	0	11.511336	5.314464	3.794647
264	H	0	9.023680	2.129383	4.049657
265	C	0	12.064029	1.800301	0.921313
266	C	0	12.064143	-1.800555	-0.921435
267	C	0	10.971278	2.317030	0.725248
268	C	0	10.971339	-2.317263	-0.725608
269	C	0	13.331081	-1.199902	-1.175241
270	C	0	14.536645	1.831192	0.780296
271	C	0	13.330934	1.199701	1.175406
272	C	0	13.378769	-0.109685	1.689662
273	C	0	14.571373	-0.818286	1.790265
274	C	0	15.731337	1.233007	1.163651
275	C	0	15.778444	-0.091065	1.644091
276	C	0	14.566945	-2.327780	1.759663
277	C	0	14.555906	-2.919233	0.267437

278	C	0	14.536725	-1.831317	-0.779803
279	C	0	14.571562	0.818160	-1.789773
280	C	0	15.778640	0.091020	-1.643248
281	C	0	15.731485	-1.233049	-1.162816
282	C	0	14.556029	2.919105	-0.266945
283	C	0	14.567047	2.327654	-1.759168
284	H	0	13.687386	-2.708597	2.288707
285	H	0	15.448540	-2.718390	2.273389
286	H	0	13.684174	-3.572082	0.164381
287	H	0	15.448663	-3.537365	0.140583
288	H	0	15.448879	3.537090	-0.140037
289	H	0	13.684394	3.572095	-0.163949
290	H	0	15.448592	2.718343	-2.272925
291	H	0	13.687435	2.708396	-2.288175
292	C	0	13.378976	0.109485	-1.689500
293	H	0	12.439323	-0.628184	1.853711
294	H	0	12.439544	0.627926	-1.853809
295	C	0	17.046391	0.713302	-1.757783
296	C	0	18.168046	1.185640	-1.767778
297	C	0	19.498365	1.684412	-1.750205
298	C	0	20.569786	0.799529	-1.511393
299	C	0	21.875110	1.278857	-1.471610
300	C	0	22.131917	2.638046	-1.670802
301	C	0	21.075495	3.521070	-1.911433
302	C	0	19.766054	3.052459	-1.952100
303	H	0	20.363186	-0.253170	-1.353590
304	H	0	22.693833	0.589593	-1.288122
305	H	0	23.152116	3.008292	-1.641677
306	H	0	21.275390	4.576536	-2.069084
307	H	0	18.941527	3.732935	-2.140062
308	H	0	16.671143	1.713640	0.914494
309	H	0	16.671253	-1.713616	-0.913384
310	C	0	17.046212	-0.713249	1.759013
311	C	0	18.167900	-1.185499	1.769434
312	C	0	19.498243	-1.684241	1.752565
313	C	0	19.765775	-3.052417	1.953796
314	C	0	21.075244	-3.521009	1.913846
315	C	0	22.131857	-2.637835	1.674606
316	C	0	21.875209	-1.278519	1.476089
317	C	0	20.569854	-0.799210	1.515155
318	H	0	18.941099	-3.733011	2.140677
319	H	0	21.275012	-4.576577	2.070972
320	H	0	23.152077	-3.008066	1.646040
321	H	0	22.694083	-0.589141	1.293701
322	H	0	20.363381	0.253591	1.357866

323	P	0	9.725246	-4.040656	-2.721125
324	C	0	8.464023	-5.151297	-3.461948
325	C	0	11.288438	-4.991205	-2.773377
326	C	0	9.954161	-2.695222	-3.943575
327	H	0	8.326071	-6.029988	-2.825317
328	H	0	7.505983	-4.629625	-3.542645
329	H	0	11.211030	-5.868518	-2.124870
330	H	0	12.091744	-4.351108	-2.401020
331	H	0	10.245218	-3.099601	-4.918253
332	H	0	10.735945	-2.029291	-3.570269
333	H	0	8.776611	-5.480323	-4.457937
334	H	0	11.511460	-5.314518	-3.795179
335	H	0	9.023933	-2.129358	-4.050190
336	P	0	9.139370	-2.421749	1.617346
337	C	0	7.649447	-2.898572	2.582714
338	C	0	9.206658	-0.596475	1.750764
339	C	0	10.554265	-3.011855	2.618234
340	H	0	6.749357	-2.524384	2.085689
341	H	0	7.578771	-3.988176	2.643254
342	H	0	8.336310	-0.147152	1.263689
343	H	0	10.102428	-0.217315	1.254415
344	H	0	10.552166	-2.558581	3.614709
345	H	0	11.475103	-2.750498	2.092664
346	H	0	7.696844	-2.490331	3.597301
347	H	0	9.225348	-0.290979	2.802178
348	H	0	10.510363	-4.100498	2.715712
349	Pt	0	-9.222344	3.173944	0.891523
350	Pt	0	9.253013	3.253001	0.566218
351	Pt	0	9.253021	-3.253166	-0.566774
352	Pt	0	-9.222517	-3.173793	-0.891610

3. Supplementary Methods

As shown in Supplementary Fig 1 and 2, compound **TPy1** and **TPy2** were synthesized through Suzuki-Miyaura reaction and Sonogashira coupling according to literature reports[1-4].

Synthesis of *R*p-PCP

A 35 mL round-bottom Schlenk flask was charged with compound ***R*p-3** (40mg, 0.021 mmol) and 10 mL of dichloromethane, then the solution was added $AgNO_3$ (40 mg, 0.23 mmol) at once, resulting in a yellowish precipitate of AgI. After 12 h at room temperature, the suspension was filtered through a glass fiber and the volume of the solution reduced to 3mL. Subsequent addition of diethyl ether resulted in the precipitation of the compound ***R*p-PCP** as a slightly yellow crystalline powder (28mg, 84%). ^{1}H NMR (400 MHz, $CDCl_3$, 298K) δ 7.63 (s, 8H), 7.05 (s, 2H), 6.67 (s, 2H), 3.47 (dt, *J* = 16.3, 11.3 Hz, 4H), 3.06 – 2.94 (m, 2H), 2.92 – 2.82 (m, 2H), 1.98 (qt, *J* = 7.6, 3.5 Hz, 24H), 1.28-1.21 (m, 36H). ^{13}C NMR (100 MHz, $CDCl_3$, 298K) δ 141.9, 140.5, 134.5, 133.7, 131.6, 130.0, 129.7, 129.5, 127.6, 125.5, 125.5, 125.4, 125.4, 92.5, 91.9, 33.0, 32.6, 14.8, 14.6, 14.5, 7.9. ^{31}P NMR (162MHZ, $CDCl_3$, 298K) *δ* 20.79 ($^1J_{Pt\text{-}P}$ = 2482.08 Hz). MS (ESI-MS): m/z calcd for $[M – 2NO_3]^{2+}$: 726.2205, found: 726.0387.

Synthesis of *R*p,*R*p-PCP-TPy1

The Compound ***R*p-PCP** (30 mg, 0.019 mmol) and **TPy1** (9.6 mg, 0.019 mmol) were weighed accurately into a glass vial. To the vial were added 10 mL of acetone and 10 ml of dichloromethane, and the reaction solution was then stirred at 60 °C for 12 h to yield a homogeneous yellow solution. Then the addition of a saturated aqueous solution of KPF_6 into the bottle with continuous stirring (10 min) precipitated the product. The reaction mixture was centrifuged, washed several times with water, and dried. Grey solid product of helicate ***R*p,*R*p-PCP-TPy1** was obtained by removing the solvent under vacuum (35mg, 88%).^{1}H NMR (400 MHz, $CDCl_3$, 298K) δ 8.65 (d, *J* = 5.7 Hz, 4H), 7.90 (d, *J* = 6.6 Hz, 4H), 7.65 (d, *J* = 2.1 Hz, 8H), 7.57 (d, *J* = 8.0 Hz, 4H), 7.18-7.14 (m, 10H), 7.10 (s, 6H), 6.67 (s, 2H), 3.51 (d, *J* = 32.0 Hz, 4H), 3.04 (s, 2H), 2.87 (d, *J* = 13.6 Hz, 2H), 1.83 (s, 24H), 1.21 (d, *J* = 8.1 Hz, 36H). ^{13}C NMR (150 MHz, $CDCl_3$, 298K) *δ* 152.5, 151.2, 146.4, 142.8, 141.8, 140.3, 134.4, 134.2, 133.0, 132.5, 131.6, 131.3, 129.8, 129.2, 128.1, 127.6, 127.3, 127.0, 125.5, 125.5, 125.3, 122.0, 92.6, 91.9, 33.2, 32.7, 14.6, 14.5, 14.4, 8.8, 8.0. ^{31}P NMR (162 MHZ, $CDCl_3$, 298K) *δ* 16.04 ($^1J_{Pt\text{-}P}$ = 2356.22 Hz). MS (ESI-MS): m/z calcd for $[M – 2PF_6]^{2+}$: 2084.9582, found: 2084.6118; m/z calcd for $[M – 3PF_6]^{3+}$: 1341.6505, found: 1341.4189; m/z calcd for $[M – 4PF_6]^{4+}$: 969.9967, found: 969.8265.

Synthesis of *R*p,*R*p-PCP-TPy2

The Compound ***R*p-PCP** (30 mg, 0.018 mmol) and **TPy2** (10 mg, 0.018 mmol) were weighed accurately into a glass vial. To the vial were added 10 mL of acetone and 10 ml of dichloromethane, and the reaction solution was then stirred at 60 °C for 12 h to yield a homogeneous yellow solution. Then the addition of a saturated aqueous solution of KPF_6 into the bottle with continuous stirring (10 min) precipitated the product. The reaction mixture was centrifuged, washed several times with water, and dried. Yellow solid product of helicate ***R*p,*R*p-PCP-TPy2** was obtained by removing the solvent under vacuum (33mg, 82%). ^{1}H NMR (400 MHz, $CDCl_3$, 298K) δ 8.66 (d, *J* = 5.6 Hz, 4H), 7.91 (s, 4H), 7.65 (t, *J* = 3.1 Hz, 8H), 7.58 (s, 4H), 7.17 (d, *J* = 1.8 Hz, 3H), 7.11 (d, *J* = 1.8 Hz, 2H), 7.01 (d, *J* = 1.8 Hz, 3H), 6.69 (d, *J* = 10.8 Hz, 5H), 3.71 (s, 6H), 3.55 (s, 4H), 3.04 (s, 2H), 2.92 – 2.83 (m, 2H), 1.84 (d, *J* = 9.6 Hz, 24H), 1.22 (d, *J* = 8.3 Hz, 36H). ^{13}C NMR (150 MHz, $CDCl_3$, 298K) *δ* 158.8, 152.4, 151.3, 147.1, 143.0, 141.8, 140.3, 135.4, 134.4, 134.2, 132.7, 132.6, 132.6, 131.6, 129.2, 127.6, 127.1, 125.5, 125.5, 125.2, 124.9, 123.1, 122.0, 113.4, 92.6, 91.9, 69.6, 55.2, 53.8, 33.2, 32.7, 31.8, 29.3, 14.6, 14.5, 14.4. ^{1}P NMR (162 MHZ, $CDCl_3$, 298K) *δ* 16.05 ($^1J_{Pt\text{-}P}$ = 2351.46 Hz). MS (ESI-MS): m/z calcd for $[M – 2PF_6]^{2+}$: 2145.0102, found: 2144.6331; m/z calcd for $[M – 3PF_6]^{3+}$: 1381.6852, found: 1381.4335; m/z calcd for $[M – 4PF_6]^{4+}$: 1000.0227, found: 999.8368.

Energy transfer efficiency and antenna effect

Calculation of energy transfer efficiency (Φ_{ET}): The energy transfer efficiency (Φ_{ET}), which indicating the fraction of the absorbed energy transferred to the acceptor, is experimentally measured as a ratio of the fluorescence intensities of the donor in the absence and presence of the acceptor (I_D and I_{DA})

$$\Phi_{ET} = 1 - \frac{I_{DA}}{I_D} \qquad (1)$$

Calculation of antenna effect of **PCP-TPy1-EsY**: The antenna effect under certain concentrations of donor and acceptor equals the ratio of the emission intensity at 552 nm of the acceptor upon excitation of the donor. Where $I_{DA,360}$ (λ_{ex} = 360 nm) and $I_{DA,500}$ (λ_{ex} = 500

nm) are the fluorescence intensities of excitation of the donor at 360 nm and direct excitation of the acceptor at 500 nm, respectively.

$$\text{Antenna effect} = \frac{I_{DA,360} - I_{D,360}}{I_{DA,500}} \qquad (2)$$

Calculation of antenna effect of **PCP-TPy1-NiR**: The antenna effect under certain concentrations of donor and acceptor equals the ratio of the emission intensity at 630 nm of the acceptor upon excitation of the donor. Where $I_{DA,360}$ (λ_{ex} = 360 nm) and $I_{DA,\,500}$ (λ_{ex} = 530 nm) are the fluorescence intensities of excitation of the donor at 360 nm and direct excitation of the acceptor at 530 nm, respectively.

$$\text{Antenna effect} = \frac{I_{DA,360} - I_{D,360}}{I_{DA,530}} \qquad (3)$$

Calculation of antenna effect of ***R*p,*R*p-PCP-TPy1-EsY**: The antenna effect under certain concentrations of donor and acceptor equals the ratio of the emission intensity at 552 nm of the acceptor upon excitation of the donor. Where $I_{DA,360}$ (λ_{ex} = 360 nm) and $I_{DA,\,500}$ (λ_{ex} = 500 nm) are the fluorescence intensities of excitation of the donor at 360 nm and direct excitation of the acceptor at 500 nm, respectively.

$$\text{Antenna effect} = \frac{I_{DA,360} - I_{D,360}}{I_{DA,500}} \qquad (4)$$

Calculation of antenna effect of ***R*p,*R*p-PCP-TPy1-NiR**: The antenna effect under certain concentrations of donor and acceptor equals the ratio of the emission intensity at 630 nm of the acceptor upon excitation of the donor. Where $I_{DA,360}$ (λ_{ex} = 360 nm) and $I_{DA,\,500}$ (λ_{ex} = 530 nm) are the fluorescence intensities of excitation of the donor at 360 nm and direct excitation of the acceptor at 530 nm, respectively.

$$\text{Antenna effect} = \frac{I_{DA,360} - I_{D,360}}{I_{DA,530}} \qquad (5)$$

PMMA films

PMMA films were obtained by drop casting a solution (200 µl) containing 10^{-3} M fluorophore in chlorobenzene (50 µl) and 20% PMMA solution in chlorobenzene (150 µL).

4. Supplementary References

1. He, J.; Yu, M.; Pang, M.; Fan, Y.; Lian, Z. et al. Nanosized Carbon Macrocycles Based on a Planar Chiral Pseudo Meta [2.2]Paracyclophane. *Chem. Eur. J*. **28**, 1-8 (2022).

2. Liu, S.; Zhang, H.; Li, Y.; Liu, J.; Du, L. et al. Strategies to Enhance the Photosensitization: Polymerization and the Donor–Acceptor Even–Odd Effect. *Angew. Chem. Int. Ed.* **57**, 15189-15193 (2018).

3. Sun, N.; Su, K.; Zhou, Z.; Wang, D.; Fery, A. et al. "Colorless-to-Black" Electrochromic and AIE-Active Polyamides: An Effective Strategy for the Highest-Contrast Electrofluorochromism. *Macromolecules*, **53**, 10117-10127 (2020).

4. Yan, X.; Wang, H.; Hauke, C. E.; Cook, T. R.; Wang, M. et al. A Suite of Tetraphenylethylene-Based Discrete Organoplatinum(II) Metallacycles: Controllable Structure and Stoichiometry, Aggregation-Induced Emission, and Nitroaromatics Sensing. *J. Am. Chem. Soc*., **137**, 15276-15286 (2015).

5. Gaussian 16, Revision A.03,M. J. Frisch, G. W. Trucks, H. B. Schlegel, G. E. Scuseria, M. A. Robb, J. R. Cheeseman, G. Scalmani, V. Barone, G. A. Petersson, H. Nakatsuji, X. Li, M. Caricato, A. V. Marenich, J. Bloino, B. G. Janesko, R. Gomperts, B. Mennucci, H. P. Hratchian, J. V. Ortiz, A. F. Izmaylov, J. L. Sonnenberg, D. Williams-Young, F. Ding, F. Lipparini, F. Egidi, J. Goings, B. Peng, A. Petrone, T. Henderson, D. Ranasinghe, V. G. Zakrzewski, J. Gao, N. Rega, G. Zheng, W. Liang, M. Hada, M. Ehara, K. Toyota, R. Fukuda, J. Hasegawa, M. Ishida, T. Nakajima, Y. Honda, O. Kitao, H. Nakai, T. Vreven, K. Throssell, J. A. Montgomery, Jr., J. E. Peralta, F. Ogliaro, M. J. Bearpark, J. J. Heyd, E. N. Brothers, K. N. Kudin, V. N. Staroverov, T. A. Keith, R. Kobayashi, J. Normand, K. Raghavachari, A. P. Rendell, J. C. Burant, S. S. Iyengar, J. Tomasi, M. Cossi, J. M. Millam, M. Klene, C. Adamo, R. Cammi, J. W. Ochterski, R. L. Martin, K. Morokuma, O. Farkas, J. B. Foresman, and D. J. Fox, Gaussian, Inc., Wallingford CT, 2016.

6. Acharyya, K.; Bhattacharyya, S.; Sepehrpour, H.; Chakraborty, S.; Lu, S. et al. Self-Assembled Fluorescent Pt(II) Metallacycles as Artificial Light-Harvesting Systems. *J. Am. Chem. Soc.* **141**, 14565-14569 (2019).

7. Hu, Y.-X.; Jia, P.-P.; Zhang, C.-W.; Xu, X.-D.; Niu, Y. et al. A supramolecular dual-donor artificial light-harvesting system with efficient visible light-harvesting capacity. *Org. Chem. Front.* **8**, 5250-5257 (2021).

8. Liu, H.; Zhang, Z.; Mu, C.; Ma, L.; Yuan, H. et al. Hexaphenylbenzene‐Based Deep Blue‐Emissive Metallacages as Donors for Light‐Harvesting Systems. *Angew. Chem. Int. Ed* (2022).

9. Diao, K.; Whitaker, D. J.; Huang, Z.; Qian, H.; Ren, D. et al. An ultralow-acceptor-content supramolecular light-harvesting system for white-light emission. *Chem. Commun.* **58**, 2343-2346 (2022).

10. Zhang, D.; Yu, W.; Li, S.; Xia, Y.; Li, X. et al. Artificial Light-Harvesting Metallacycle System with Sequential Energy Transfer for Photochemical Catalysis. *J. Am. Chem. Soc.* **143**, 1313-1317 (2021).

11. Li, X.-L.; Wang, Y.; Song, A.; Zhang, M.-H.; Jiang, M. et al. The construction of an artificial light-harvesting system with two-step sequential energy transfer based on supramolecular polymers. *Soft Matter* **17**, 9871-9875 (2021).

12. Li, J.-J.; Zhang, H.-Y.; Dai, X.-Y.; Liu, Z.-X.; Liu, Y., A highly efficient light-harvesting system with sequential energy transfer based on a multicharged supramolecular assembly. *Chem. Commun.* **56**, 5949-5952 (2020).

13. Hao, M.; Sun, G.; Zuo, M.; Xu, Z.; Chen, Y. et al. A Supramolecular Artificial Light‐Harvesting System with Two‐Step Sequential Energy Transfer for Photochemical Catalysis. *Angew. Chem. Int. Ed.* **59**, 10095-10100 (2019).

14. Acharyya, K.; Bhattacharyya, S.; Lu, S.; Sun, Y.; Mukherjee, P. S. et al. Emissive Platinum(II) Macrocycles as Tunable Cascade Energy Transfer Scaffolds. *Angew. Chem. Int. Ed***. 61**, (2022).
